# Weak hydrogen bonding as the driver of aromatic hydration

**DOI:** 10.1038/s41467-026-76528-x

**Published:** 2026-08-11

**Authors:** Camilla Di Mino, Daniel Bowron, Michael A. Wilkinson, Tristan G. Youngs, Thomas F. Headen, Andrea Sella, Neal T. Skipper

**Affiliations:** 1 Department of Physics and Astronomy, UCL, London, UK; 2 Physical and Theoretical Chemistry Laboratory, Department of Chemistry, University of Oxford, Oxford, UK; 3 ISIS Neutron and Muon Source, RAL, Didcot, UK; 4 Department of Chemistry, UCL, London, UK

**Keywords:** Chemical physics, Chemical physics

## Abstract

Intermolecular forces are the fundamental architects of supramolecular structure, where subtle interplays of charge distribution, entropy, and sterics determine the outcome. In aromatic molecules, the distribution of delocalised *π* electrons is modulated by the substituents, affecting their intermolecular interactions and introducing “holes” in the *π* orbitals. While increasingly well-understood in the solid state, the influence of *π*-holes on solvation and miscibility in the liquid remains unknown. Here, total neutron scattering and modelling-based refinement reveal the solvation of phenol, aniline, and p-nitrophenol in water. The in-plane solvation is dominated by strong classical hydrogen bonds between water and the substituents. Out of the ring plane, perpendicular OH···*π* weak hydrogen bonds between water and phenol or aniline are cooperative and modulated by differences in electron density. By contrast, in p-nitrophenol, the presence of the electron-withdrawing nitro group enhances the overall molecular dipole, and introduces a pronounced *π*-hole that significantly disrupts the overall out-of-plane solvation. The latter is dominated by close water-O···N contacts at ≈ 3.35 Å resulting from localized charge depletion accompanied by O···π* motifs (≈ 3.96 Å). These interactions template the structure of the surrounding water, redefining the solubility of these aromatics in water and underscoring the complex solvation of organic molecules.

## Abstract (web summary)

The influence of *π*-holes on solvation and miscibility of aromatic molecules in water remains unclear. Here, the authors show that aromatic hydration is dominated by weak OH···*π* hydrogen bonds rather than by conventional hydrogen bonds via the substituents.

## Introduction

Aromatic rings are ubiquitous in chemical and biological sciences, as they provide a functionalisable and rigid platform on which to build larger assemblies. Nucleic acids for example owe the specificity of their molecular recognition to the rigidity of the aromatic base pairs; among other biomolecules, four amino acids contain aromatic groups, while planar prosthetic groups based around porphyrin (e.g., heme, chlorophyll) offer both rigidity and delocalization. At the same time numerous pharmaceuticals (e.g., nonsteroidal anti-inflammatory drugs like ibuprofen) have aromatic cores. The interaction of these units with other molecules, especially with water, is a key driver of the three-dimensional folding structure of macromolecules such as DNA, proteins, and the binding of substrates, inhibitors, and signalling molecules to proteins in cells. Moreover, the design and development of more effective pharmaceuticals will therefore depend considerably on our understanding of the way in which aromatics interact with each other and other molecules^1–7^. Beyond biologically related systems, water aromatic interactions dictate the structure of interfacial and nanoconfined water with impact on transport phenomena, interfacial self-assembly, and molecular adsorption^8–10^.

At the heart of aromatic structures sits the benzene ring, one of the central paradigms of chemistry. From the original proposal of a symmetrical cyclic structure by Loschmidt, and, possibly independently, by Kekulé, to the quantum description of aromaticity advanced by Hückel (the 4*n* + 2 rule, with *n* the number of *π* electrons), benzene has played a key role in advancing our understanding of chemical structure and bonding^11–13^. Aromaticity is characterised by a distinctive molecular structure arising from the cyclic arrangement of conjugated *π* orbitals, with delocalised electrons sitting over the ring, enforcing its planarity.

The chemistry of these molecules, including their solubility in water, is controlled by the energies and charge distributions of the highest and lowest occupied molecular orbitals (HOMO/LUMO) both of which have *π* symmetry with respect to the C-C bonds; these change significantly when a functional group is substituted onto the aromatic ring. For example, *π* electron-donating substituents such as OH and NH_2_ increase the electron density of the aromatic ring by totally or partially delocalising the oxygen and nitrogen lone pairs into it, which raises the energy of the HOMO and LUMO, making the ring more “electron-rich”. Conversely, *π*-electron withdrawing groups, such as NO_2_, lower the energy of the molecular orbitals. The changes in energy are accompanied by regions of increased or depleted charge that govern the interaction with other molecules. Electron distributions and molecular reactivities can be evaluated using a combination of theoretical methods and spectroscopic techniques^14–16^.

In the context of aromatic/water interactions, there is good evidence that the delocalised electrons can act as hydrogen bonding acceptors, whereas the regions of depleted electron density, the so-called *π*-holes, can interact with atoms of (partial) negative charge^17–26^. These contacts have been observed both in the gas phase, where they span distances from 2.45 to 3.2 Å, and in solid-state supramolecular chemistry, where they control crystal growth, packing, and stability^24–27^. However, such interactions have not yet been observed in the liquid state. The extent to which they contribute to solvation and preserve the geometries and directionalities seen in crystal structures and gas clusters is therefore unclear. Nuclear magnetic resonance is a powerful tool to identify and quantify the effects of non-covalent interactions both in the solid and in the liquid state. These include intra and intermolecular classical hydrogen bonding, X-H···*π* interactions, and non-conventional lone pair-π*^27–30^.

**Fig. 1:**
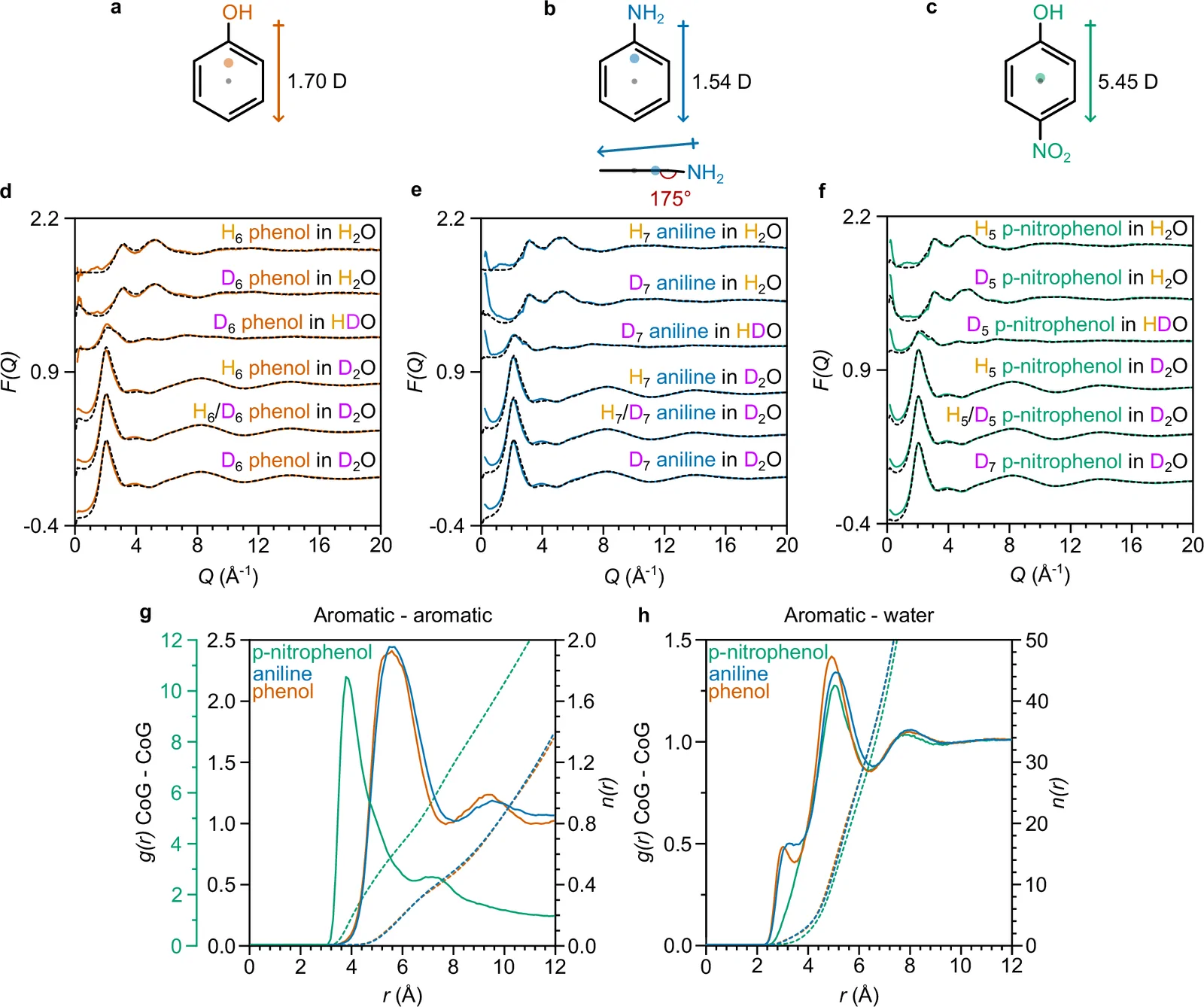
Total neutron scattering data for aqueous solutions of phenol, aniline, and p-nitrophenol and definition of the solvation shells. **a–c** Schematic of the three selected aromatics phenol (orange), aniline (blue), and p-nitrophenol (green). The arrows indicate the direction of the molecular dipole moment vector^48,74^. Note that the dipole moment of aniline is tilted out of the plane of the ring due to the tetrahedral geometry of the amino group^47^. The grey dot indicates the position of the centre-of-the-ring (CoR) while the coloured dot indicates the approximate position of the centre-of-geometry (CoG) where the molecular centre is obtained by averaging the atoms positions. **d–f** Experimental total structure factors, *F(Q)*, (solid line) plotted against the Dissolve model after structural refinement (dashed line) for each isotopically distinct sample. Hydrogen and deuterium substitution highlighted in pink and orange. **g** Partial radial distribution functions *g*_CoG-CoG_*(r)* relative to the aromatic CoG (solid line) plotted against the running coordination number *n*_CoG-CoG_*(r)* (dashed line) for phenol-phenol (orange), aniline-aniline (blue), and p-nitrophenol-p-nitrophenol (green); **h** phenol-water (orange), aniline-water (blue), and p-nitrophenol-water (green). The high intensity peak of p-nitrophenol indicates formation of antiparallel aromatic dimers, driven by the high dipole moment of p-nitrophenol (Suppl. Fig. 22). Source data are provided as a Source Data file.

Here, we combined the high sensitivity to hydrogens of total neutron scattering (TNS) with extensive hydrogen/deuterium isotopic substitution and simulation-based refinement, to reveal the hydration of the archetypal substituted aromatics phenol, aniline, and p-nitrophenol. The introduction of hydrogen bonding donor and acceptor sites in substituted aromatics greatly increase their solubility in water. For example, phenol (C_6_H_5_OH, Fig. 1a) and aniline (C_6_H_5_NH_2_, Fig. 1b) see their solubility in water increase by over an order of magnitude (0.895 and 0.39 M, respectively) compared to benzene (≈ 0.02 M)^31–34^. Yet, p-nitrophenol (O_2_NC_6_H_5_OH, Fig. 1c) is significantly less soluble than phenol in water (0.115 vs. 0.895 M) despite sharing an OH substituent, suggesting that the nitro group, while being a strong hydrogen bonding acceptor, causes significant disruption to the interactions with the surrounding water^35,36^.

Previous studies on phenol-water and aniline-water systems have identified three principal inter-molecular interactions: classical hydrogen bonding between the OH/NH_2_ functional groups and water, weak CH···O hydrogen bonding involving the CH groups of the aromatic ring, and OH···*π* interactions, where a water molecule approaches the ring out-of-plane bonding to the delocalised electrons^37^. Classical simulations of the OH···*π* interaction predict hydrogen to centroid distances of ≈ 2.2 Å for phenol and 2.3 Å for aniline, with the number of *π*-coordinated water molecules being on average 1.53 and 0.84, respectively, consistent with the electron donating power of hydroxyl and amine groups^38–40^. For both molecules, the estimated OH···*π* interaction is weak (10 and 2.5 kJ mol^−1^) for phenol and aniline, respectively, but present on average in a higher number of solute/solvent pairs than for benzene-methanol, where the corresponding average coordination number was 0.5^18,41,42^. Moreover, the OH···*π* interaction modelled in quantum calculations of phenol-water (2.33 Å) and aniline-water (2.86 Å) clusters is found at longer distances than classical models, indicating the importance of studying these bonds in the presence of many other molecules, where molecular environments, many-body terms, and cooperation are interdependent^41,42^.

The solvation of aromatics by water, despite its fundamental importance, remains an area that has been underexplored experimentally; a complete characterisation of the conventional and non-conventional interactions involved is still lacking for many simple systems. Looking at the hydration of phenol, aniline, and p-nitrophenol, we provide a view to quantifying both classical and unconventional aromatic/water molecular interactions in the liquid state, and to shed light on the intricacies of the dissolution mechanisms of substituted aromatics in water.

## Results

The experimental data on the six isotopically distinct phenol, aniline, and p-nitrophenol samples are presented in Fig. 1d–f plotted against the Dissolve-refined model (Suppl. Table 1, Supplementary Figs. 1–18). The total structure factors modelled by Dissolve reproduce both peak intensities and positions of the experimental data in the intermolecular range (0.2–1.5 Å^−1^). The small discrepancies between the model and the data are visible at *Q* values < 0.2 Å^−1^ are the result of residual contributions from multiple and inelastic scattering events characteristic of hydrogen-rich materials^43^. Further, the neutron data do not present any intense, low-*Q* scattering signal that could be ascribed to clustering at the nano/meso-scale. The absence of such aggregates is, in turn, indicative that phase separation at this concentration does not occur; a crucial requirement for the study of the aromatic solvation in absence of strong solute-solute contacts. Concurrently, a measurable contribution to the experimental neutron scattering signal is still provided (Suppl. Tables 1–4).

To exclude the presence of molecular clusters across shorter length scales (< 30 Å), we can plot the partial radial distribution functions for intermolecular solute-solute and solute-water centres-of-geometry (CoG-CoG)^44^. The first peak of the solute-solute *g*_CoG-CoG_*(r)* (Fig. 1g) is found at a separation of 5.6 and 5.7 Å for phenol (orange) and aniline (blue) respectively, with coordination numbers of 0.45 and 0.3 (Suppl. Table 5). A second aromatic molecule approaching phenol and aniline does not show any preferential relative orientation (Suppl. Fig. 19), suggesting negligible solute-solute interactions (Suppl. Figs. 20, 21). The nearest CoG-CoG contact for p-nitrophenol (green) is found at shorter distances (3.9 Å) and this peak is remarkably more intense than for phenol-phenol and aniline-aniline reaching an intensity of 10. In addition, the number of nearest neighbours of the same species for p-nitrophenol is ≈ 0.73. The high molecular dipole of p-nitrophenol strongly affects their relative arrangement with a preference for close antiparallel alignment, differently from the solid state where it exhibits parallel stacking (Suppl. Fig. 22)^45^. Solute-solute hydrogen bonding is not thought to have a strong effect on these contacts, given the almost negligible coordination numbers (Suppl. Table 6). The observation is confirmed by the increase of the percentage of 1 or 2 contacts, at the expense of independent monomers, and it is consistent with the lower solubility of p-nitrophenol that has effects even in this diluted regime (Suppl. Table 9). By contrast, for phenol and aniline the number of nearest neighbours is consistent with an average much looser association. These solute-solute coordination numbers implicitly contain information about the average self-association of these substituted aromatics in water.

In their first solvation shell, both phenol and aniline host ≈ 33 water molecules (Suppl. Table 5). Although p-nitrophenol has a larger molecular volume than the two other substituted aromatics, it is surrounded by fewer water molecules (≈ 29), as direct evidence of perturbed water-ring interactions that are tighter in the case of phenol and aniline. Possibly, the strongly templating influence of p-nitrophenol on a second aromatic molecule may also hinder the volume accessible on either side of the ring plane. Owing to their planar geometry, the aromatic-water *g*_CoG-CoG_*(r)* present a peak at 3.0 and 3.20 Å respectively (Fig. 1h), and distances consistent with an out-of-plane approach. For p-nitrophenol, the corresponding peak is absent, and features associated with the approach of water are displayed to much longer distances. This observation is consistent with the OH···*π* interaction being disrupted, and it is direct evidence of different ring-water interactions that may include the concomitant rising of the unconventional lone pair-*π*-hole contacts.

### Classical hydrogen bonds between water and the substituents

The different contributions to the solvation of these substituted benzenes in water have indeed multiple origins. To understand the role of these competitive interactions in the complex solvation behaviour of aromatics by water, we can look at the site-site partial distribution functions *g*_αβ_*(r)* associated with atomic sites on each solute and solvent molecule. To establish the ability of phenol, aniline, and p-nitrophenol  to form classical hydrogen bonds with the surrounding water, we plot the partial distribution functions *g*_H-Ow_*(r)*, *g*_O-Hw_*(r)*, and *g*_N-Hw_*(r)* related to the hydroxyl and amino groups. Note that to distinguish the atomic sites of water, they are denoted with a subscript W.

**Fig. 2:**
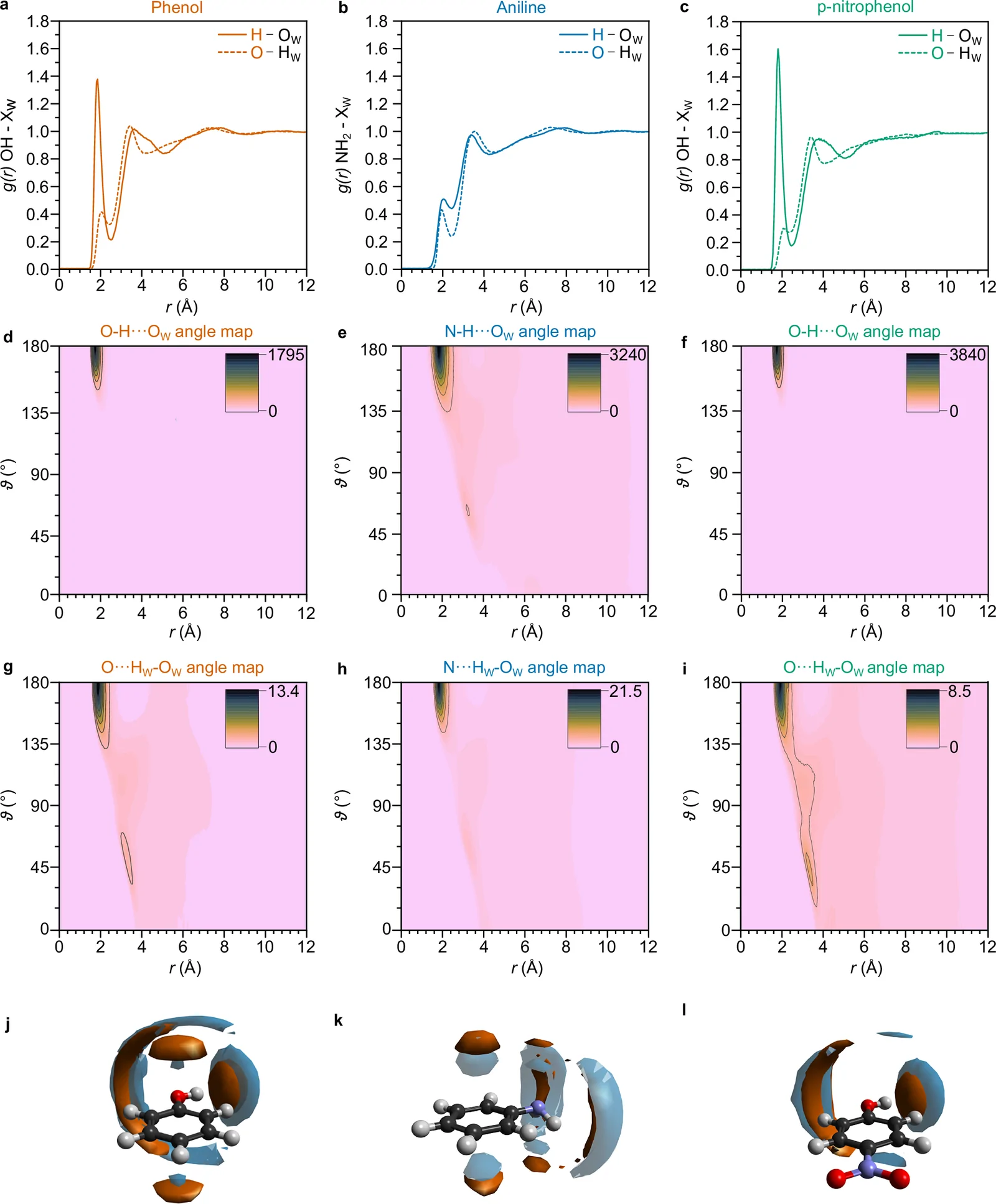
Hydration of the OH and NH_2_ substituents. **a** Partial radial distribution functions *g*_H-Ow_*(r)* (solid line) and *g*_O-Hw_ *(r)* (dashed line) of phenol hydroxyl group OH and water (orange). **b** *g*_H-Ow_*(r)* (solid line) and g_N-Hw_ (r) (dashed line) of aniline’s amino group NH_2_ and water (blue); and **c** *g*_H-Ow_*(r)* (solid line) and g_O-Hw_ (r) (dashed line) of p-nitrophenol hydroxyl group OH and water (green). Note that the amino group is a less strong hydrogen bonding donor than the OH group. **d** O-H···O_W_ angle map for phenol; **e** N-H···O_W_ angle map for aniline; and **f** O-H···O_W_ angle map for p-nitrophenol, highlighting their role as hydrogen bonding donors. The sharp peak at ≈ 180° indicates that the hydrogen bonding is linear; **g** O···H_W_-O_W_ angle map for phenol; **h** N···H_W_-O_W_ angle map for aniline; and **i** O···H_W_-O_W_ angle map for p-nitrophenol, highlighting their role as hydrogen bonding acceptors. **j**–**l** SDFs showing the 15% more likely position of water hydrogen (blue) and oxygen (orange) around the hydroxyl and amine groups. Note that the XH···X is linear in all three cases. Source data are provided as a Source Data file.

The first peaks of the phenol and p-nitrophenol *g*_H-Ow_*(r)* occur at 1.80 and 1.77 Å, respectively (Fig. 2a, c). These distances are comparable to what is observed in pure water (H_W_···O_W_ ≈ 1.85 Å)^46^. The O-H···O_W_ angle maps (Fig. 2d, f) indicate that the classical O-H···O_W_ hydrogen bonds are linear. The *g*_H-Ow_*(r)* for aniline-water presents a peak that is less sharp and slightly further at ≈ 1.95 Å than in the case of the hydroxyl groups (Fig. 2b), and that in three dimensions translates to a broader N-H···Ow angle distribution (Fig. 2e). The different hydrogen-donating ability of aniline is ascribed to the longer nature of N-H intermolecular bonds due to the lower electronegativity of nitrogen, and to the non-planar geometry of the amino group^47–49^. The hydroxyl groups of phenol and p-nitrophenol donate ≈ 1 hydrogen bond to water, while the H-O_W_ coordination number for aniline is ≈ 0.7, consistent with a reduced bonding ability due to the geometry of the NH_2_ group and the low energy barrier of NH_2_ inversion (Suppl. Table 7) ^35^.

Interestingly, the OH groups and the NH_2_ group of phenol, p-nitrophenol, and aniline show comparable hydrogen bond accepting behaviours (Fig. 2g–i). All three aromatics accept fewer hydrogen bonds via the substituents due to the total (for phenol and p-nitrophenol), and partial (for aniline) delocalisation of the oxygen and nitrogen lone pairs into the ring. The O-H_W_ coordination number in phenol is 1.1, and decreases to ≈ 0.8 in the case of p-nitrophenol where the oxygen lone-pair is further depleted by the presence of the nitro group (Suppl. Table 7). The N-H_W_ coordination number is ≈ 0.9, closer to the hydrogen-bonding accepting ability of nitrogen that is 1. The spatial density functions (SDFs) of Fig. 2j–l help us to visualise the three-dimensional arrangement of water hydrogen (blue), and water oxygen (orange) around the substituents. In phenol and p-nitrophenol, the water oxygen sits in line with the OH vector in a highly localised lobe, while the hydrogen atoms are always found at longer distances. The phenolic oxygen is surrounded by a blue crescent showing average position of the closest water hydrogens that reflects the free rotation of the OH group. In the case of aniline, the N-H···O_W_ is directional and reflects the low inversion barrier of the NH_2_ relative to the aromatic plane, while above (and below) the amine nitrogen we see a highly localised water hydrogen cloud^49^.

**Fig. 3:**
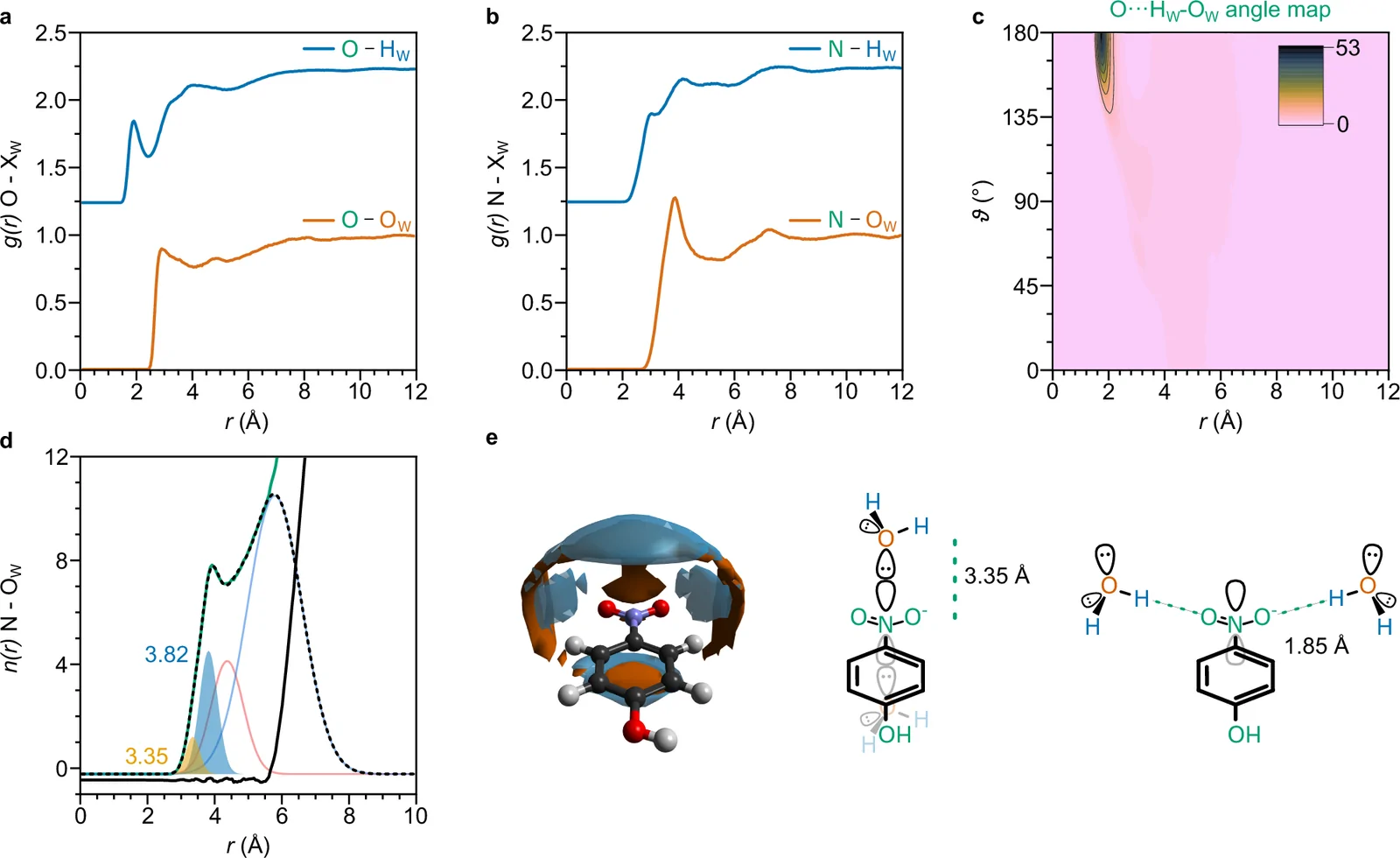
Hydration of the NO_2_ group. **a** Site-site partial radial distribution functions *g*_O-Hw_*(r)* (blue) and *g*_O-Ow_*(r)* (orange) between the nitro group NO_2_ and water, offset for clarity. Note the short O···H_W_ inter-molecular distance. **b** Site-site partial radial distribution functions *g*_N-Hw_*(r)* (blue) and *g*_N-Ow_*(r)* (orange) between the nitro group NO_2_ and water, offset for clarity. Note the short O···H_W_ inter-molecular distance. **c** O···H_W_-O_W_ angle map showing a highly directional hydrogen bonding interaction involving the nitro group. **d** Gaussian fitting of the running coordination number *n*_N-Ow_*(r)*. **e** SDFs showing the 15% most likely position of water hydrogen (blue) and water oxygen (orange) around the nitro group. Schematic of the proposed molecular arrangements. The symmetry of the lobes reflects the symmetry of the nitro group, and for the latter reason molecules are shaded. Note the highly localised lobes above and below the nitrogen atom, attributed to a highly directional lone pair-hole interaction. Source data are provided as a Source Data file.

The nitro group is involved as well in highly directional classical hydrogen bonds with water via the distinctly negative oxygen sites (Fig. 3a, c). The partial *g*_O-Hw_*(r)* of Fig. 3a indicates that the O···H_W_ bonding distance is ≈ 1.85 with average coordination number of 1.4 (Suppl. Table 7). It is interesting to note that the water structuring around the nitro group in solution is similar to that observed previously in the solid state (Fig. 3e)^50,51^. From our analysis, it is clear that phenol and p-nitrophenol share the same classical hydrogen bonding abilities via the substituents, and we argue that the nitro group in p-nitrophenol constitutes an additional site that should increase interaction with water and therefore solubility. However, the evidence suggests the opposite, we therefore turn to look at the out-of-plane solvation.

### Weak OH···*π* hydrogen bonding

**Fig. 4:**
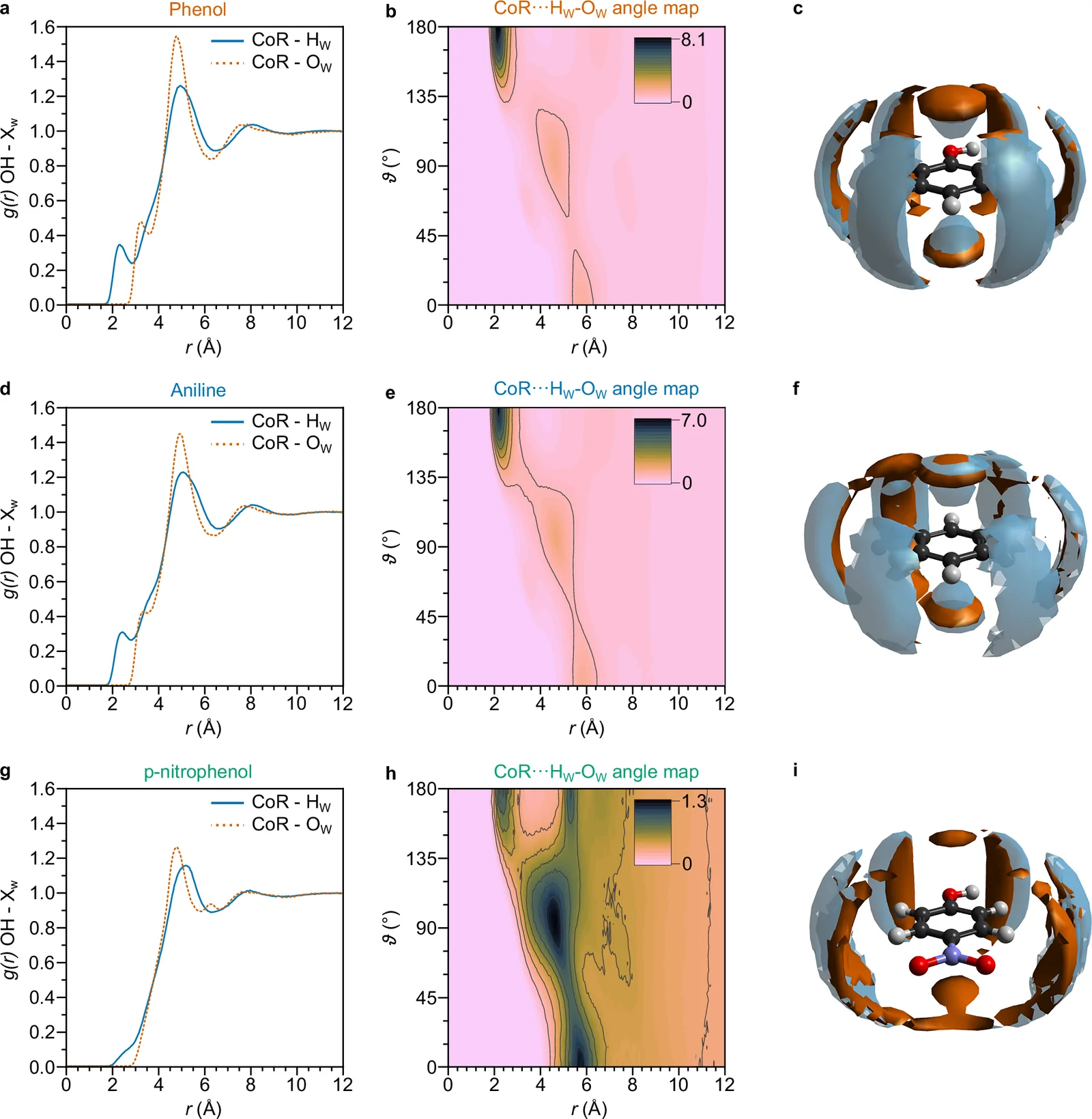
Non-conventional OH···*π* interaction. **a**, **d**, **g** Partial radial distribution functions *g*_CoR-Hw_*(r)* and *g*_CoR-Ow_*(r)* relative to the aromatic CoR and the water hydrogen (blue, solid) the water oxygen (orange, dashed) for phenol-water, aniline-water, and p-nitrophenol. **b**, **e**, **h** CoR···H_W_-O_W_ angle map showing the relative angle between the aromatic CoR, the hydrogen and the oxygen atoms for phenol-water, aniline-water, and p-nitrophenol; **c**, **f**, **i** SDFs showing the 5% most likely position of water hydrogen (blue) and water oxygen (orange) up to 7 Å from the aromatic CoR. The OH···*π* interaction out-of-plane decreases in intensity and directionality from phenol, to aniline to p-nitrophenol while in-plane the C-H groups form bifurcated hydrogen bonds with water oxygen. Source data are provided as a Source Data file.

**Fig. 5:**
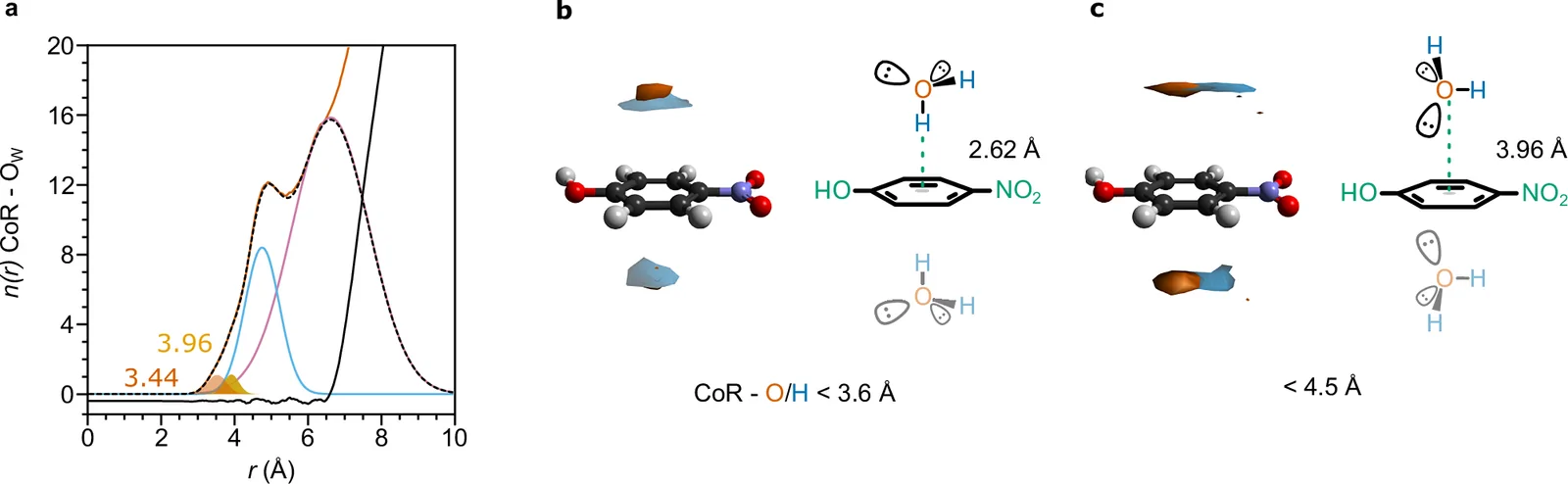
Analysis of the O···π* contacts. **a** Gaussian fitting of the running coordination number *n*_CoR-Ow_*(r)*. **b**, **c** SDFs showing the 15% most likely position of water hydrogen (blue) and water oxygen (orange) around the p-nitrophenol CoR integrated up to 3.6 and 4.5 Å respectively. Schematic of the proposed interaction types. The symmetry of the lobes reflects the symmetry of the aromatic, and for the latter reason are faded in the schematic. The average CoR···H_W_ distance is determined from Gaussian fitting (Suppl. Fig. 26). Source data are provided as a Source Data file.

It is now well-established from a variety of studies that the *π* cloud of an aromatic molecule can interact with OH groups, with the OH vector pointing towards the centre of the aromatic ring (CoR)^18,20,21^. To investigate this bonding motif for the three substituted aromatics, we can look at the partial radial distribution functions involving the water and the CoR *g*_CoR-Hw_*(r)* and *g*_CoR-Ow_*(r)*. The first peak in the *g*_CoR-Hw_*(r)* peak is found at 2.29 Å for phenol, 2.37 Å for aniline, and ≈ 2.5 Å for p-nitrophenol (Fig. 4a, d, g). Because these distances are averaged over three-dimensional space, the CoR-H_W_ distance corresponding to a directional interaction as a function of the relative CoR···H_W_-O_W_ angle is slightly shorter than the value obtained from the one-dimensional partial radial distribution functions *g(r)*s. The directionality of the O_W_-H_W_···*π* bond can be assessed by looking at the partial distribution functions *g*_CoR-Ow_*(r)* of Fig. 4. The peak positions follow the same trend seen for the CoR-H_W_ (phenol < aniline < p-nitrophenol), but the *g*_CoR-Ow_*(r)* peak for p-nitrophenol is not present at comparable distances with the other two aromatics. For phenol and aniline, the oxygen is found at distances of 3.2 and 3.3 Å from the CoR, and lies within the range of the intramolecular OH and NH distances (0.95 and 1.01 Å, respectively). These distances indicate that the OH···*π* is highly directional, as seen from the angle maps of Fig. 4b, e and the SDFs of Fig. 4c, f, where the hydrogen (blue) and oxygen (orange) lobes lie directly above the phenol and aniline aromatic planes. By contrast, for p-nitrophenol the peak lies beneath the broader second peak of the *g*_CoR-Ow_*(r)* at a distance of 3.44 Å as obtained by Gaussian fitting (Fig. 5a, Suppl. Table 10), leaving on average just a localised oxygen distribution above and below the ring (Fig. 4i).

The average length of the O-H···*π* interaction at the maximum of its directionality and its average geometry can be obtained from the CoR···H_W_-O_W_ angle maps for the three aromatics. These maps show a clear peak at 180° at 2.15, 2.20, and 2.35 Å distance between H_W_ and phenol, aniline, and p-nitrophenol CoR, respectively (Fig. 4b, e, h); however, the intensity for p-nitrophenol is strikingly lower than the other two aromatics, with the solvation at longer distances more pronounced. Although the simultaneous presence of an electron-donating and an electron-withdrawing group reduces the electron density of the ring, the O-H···*π* interaction is still present between p-nitrophenol and water, demonstrating the high level of stability of such interaction in the liquid state.

The occurrence of the O-H···*π* hydrogen bonding interaction can be evaluated from the cumulative coordination numbers CoR–H_W_ relative to the aromatic CoR and the water hydrogens. The number of bonds out of the plane decreases from phenol to aniline (1.2 and ≈1, respectively), and there is a dramatic drop for p-nitrophenol (0.24; Suppl. Table 10). This confirms that the OH·﻿·﻿·*π* interaction is weak, and that the number of contacts scales with the electron-donating character of OH and NH_2_. When nitro groups are present, we see a dramatic decrease in the occurrence of this interaction, consistent with the lower electron richness of the *π* system. Interestingly, the probability of 1 or more contacts decreases from 83% to 23%, from phenol to p-nitrophenol, showing that while phenol is almost always involved in at least one OH···*π* interaction, for p-nitrophenol it is a rare event.

Given the remarkably lower solubility of p-nitrophenol, we propose that it is the contribution of the OH···*π* that makes a difference to the macroscopic properties. This interaction on its own is clearly insufficient, otherwise simple small aromatics such as benzene and toluene would be more soluble in water. Therefore, we can conclude that it is the ability of such non-classical bonds to participate in cooperative mechanisms that is decisive^35^. A similar argument can be made for aniline, whose solubility is half the one of phenol^33,34^. The partial delocalisation of the nitrogen lone pair over the ring fails to enforce the planarity of the NH_2_ group (and vice versa) while reducing the ring’s electron richness compared to phenol. Consequently, the water structure around aniline will be further disrupted than in the case of phenol by the low flipping barrier that hiders the close approach of a water molecule, and concurrently reduces the number of hydrogen bonds (Suppl. Table 6).

### The rise of lone pair-hole O···N and O···π* interactions

The presence of the OH···*π* interaction in p-nitrophenol does not preclude the rise of additional contacts at different relative geometries between the aromatic ring and a water molecule. Such an interaction may be the result of the strong polarisation induced by the electron-withdrawing nitro group, which introduces a positive hole in the electron density which can get in close contact with a non-bonding electron pair of a water molecule. Gaussian analysis of the running coordination number *n*_CoR-Ow_*(r)* indicates a second peak at ≈ 3.96 Å (Fig. 5a). The SDFs for phenol and aniline show a highly localised water structuring above and below the aromatic ring, with the hydrogen (blue) and oxygen (orange) density located directly above the ring centroid (Fig. 5b, c). By contrast, at the same integration distance and probability threshold, the water distribution above and below the p-nitrophenol ring is reduced to the oxygen atoms only (Fig. 4i). Furthermore, for p-nitrophenol, several features arise as a function of the integration limit. Below 3.6 Å, the hydrogen and oxygen SDFs show the previously mentioned disrupted OH···*π* interaction (Fig. 5b), but the latter vanishes in the < 4.5 Å range, where there are more oxygen atoms with hydrogens parallel to, or pointing away from, the plane of the aromatic ring (Fig. 5c). Although this motif might arise simply from the structuring of the hydrogen bonded network of water around the nitro group, the distance and the directionality support the notion of an actual close contact between the non-bonding electrons of the oxygen and the region of positive charge (*π*-hole) that is present in the electron density distribution of this highly polarised molecule. In fact, the oxygen SDF sits slightly off-set towards the OH group, with the oxygen lone-pair in the direction of the LUMO^16^. Although we are not sensitive directly to electron distributions in our analysis, we can take our data as structural evidence for *π*-hole···O_W_ bonding in the liquid state, where the geometry of such an interaction is best described by the oxygen lone pair pointing towards the aromatic ring (Fig. 5c)^52^.

Similarly, the region of positive charge centred between the *para* carbon of the ring and the nitrogen atom causes a water molecule to be drawn, oxygen-first, towards it. This interaction can be seen in the partial *g*_N-Ow_*(r)* (Fig. 3b). The first strong peak at 3.82 Å corresponds to the network of hydrogen-bonded water molecules sitting in the plane interacting with the oxygens of the nitro group via (N)O···H_W_-O_W_. However, Gaussian fitting of the *n*_N-Ow_*(r)* indicates the clear presence of an additional overlapping component with a maximum at 3.35 Å (Fig. 3d, Suppl. Table 10). We ascribe this feature to the close approach of water molecules to the *π*-hole above/below the plane of the aromatic, in an inverted orientation compared to the OH···*π* interactions observed for the other molecules. This “inverted” geometry is clear from the SDFs of Fig. 3e, where the localised water oxygen cloud (orange) lies directly above/below the nitro group; a blue ring at a further distance is associated with the hydrogen atoms that link into the wider hydrogen bonding network and reflect what has been previously observed in crystalline systems^25,50,52^.

These non-conventional contacts have a clear effect on the overall hydration of p-nitrophenol, decreasing the water coordination from the theoretical value (from 36 to 29, Suppl. Table 5). The decrease can be ascribed to the concurrent lack of cooperation that does not facilitate water close approaches, with the consequent looser water structuring arising from the “linear” water dimers above and below the ring. In addition, there is a higher degree of self-association driven by dipole-dipole interactions that contribute to a decrease in solubility.

At much longer integration distances (< 7 Å), the in-plane solvation of the aromatics arises. The SDFs of Fig. 4 show a rich water structuring around the ring. All three aromatics form bifurcated CH···O_W_ hydrogen bonds with water, with the hydrogen atom pointing away from the ring in vertical crescents. The spatial density evolves along the *z* axis, perpendicular to the aromatic ring, and is disrupted in the *xy* plane in the proximity of the functional groups where they engage in classical hydrogen bonds (Fig. 2).

Our study gives a detailed understanding of the solvation of three substituted aromatics phenol, aniline, and p-nitrophenol, in water. Our analysis shows a rich solvation behaviour that changes systematically with the electron-donating and withdrawing power of the substituents. Thanks to the enhanced solubility of substituted aromatics in water, we have investigated the role of classical hydrogen bonding in the overall solvation, which clearly plays a central role in increasing the solubility of these molecules compared to benzene. The hydroxyl groups in phenol and p-nitrophenol, and the amino group in aniline, simultaneously donate and accept conventional hydrogen bonds of the type XH···X, at distances comparable to what is seen in bulk water and liquid ammonia^46,53^.

Our work highlights an underappreciated feature of aromatic solvation in water: the presence of non-classical interactions. OH···*π* contacts are seen in all three cases, but they are particularly weak in p-nitrophenol where we find evidence for the approach of water oxygen to the nitrogen of the nitro group (O_W_···N ≈ 3.35 Å) and the p-nitrophenol CoR (O_W_···CoR ≈ 3.96 Å) consistent with a lone pair*···π*-hole interaction. Thus, the disruption to the hydrogen bond network around the molecule arising from these changes suggests that they are at least in part responsible for the significantly reduced solubility of p-nitrophenol in spite of the otherwise-traditional hydrogen bonds^35^. While understanding the effects of electron-withdrawing groups in isolation is highly desirable, such as in the case of nitrobenzene, p-nitrophenol is at the edge of solubility to make these measurements accessible. Such a study on nitrobenzene would be possible, for example, by growing amorphous ice around the hydrophobic species^54^. Our analysis clearly shows the importance of solvation mechanisms in our understanding of weak competitive interactions, with the aim of informing the modelling of their effects on more complex behaviours, such as protein-ligand binding^14–16,55^.

## Methods

### Total neutron scattering

Total neutron scattering (TNS) is ideally suited to the study of intermolecular interactions in complex hydrogenous liquids, due to the high sensitivity to hydrogen/deuterium (H/D) isotopic exchange, and the high spatial resolution achievable from the wide range of momentum transferred, **Q**. TNS not only allows us to sample the real space at ≈ 0.1 Å, but has also proved to be key for advancing our understanding of complex solution behaviours of liquid aromatics (benzene and toluene), heteroaromatics, and aromatic mixtures^56–59^.

In a TNS experiment, for an isotropic liquid, we measure an experimental total structure factor *F(Q)*. The total structure factor is a linear combination of the Faber-Ziman partial structure factor ${S}_{{{\upalpha }}{{\upbeta }}}(Q),$ weighted with the coherent neutron scattering length ${b}_{{{\upalpha }}}$ and ${b}_{{{\upbeta }}}$ of the single species α and β, and their atomic concentration ${c}_{{{\upalpha }}}$ and ${c}_{{{\upbeta }}}$

1 $$F\left(Q\right)=\sum_{{{\upalpha }},{{\upbeta }}\ge {{\upalpha }}}\left(2-{\delta }_{{{\upalpha }}{{\upbeta }}}\right){b}_{{{\upalpha }}}{b}_{{{\upbeta }}}{c}_{{{\upalpha }}}{c}_{{{\upbeta }}}({S}_{{{\upalpha }}{{\upbeta }}}\left(Q\right)-1)$$

where $Q=4{{\rm{\pi }}}\,\frac{\sin \theta }{\lambda }$ is the magnitude of the neutron scattering vector, and ${\delta }_{{{\upalpha }}{{\upbeta }}}$ is the Kronecker delta^60^. From Eq. (1) we can define the neutron weightings as

2 $${w}_{{{\upalpha }}{{\upbeta }}}=\,\left(2-{\delta }_{{{\upalpha }}{{\upbeta }}}\right){b}_{{{\upalpha }}}{b}_{{{\upbeta }}}{c}_{{{\upalpha }}}{c}_{{{\upbeta }}}$$

t﻿hat will determine the neutron contrasts as a function of the isotopic substitution. The Fourier transformation of the total structure factors is the total radial distribution function *G(r)* defined as:

3 $$G(r)=\sum_{\upalpha,\upbeta \ge \upalpha }(2-{\delta }_{\upalpha \upbeta }){b}_{\upalpha }{b}_{\upbeta }{c}_{\upalpha }{c}_{\upbeta }({g}_{\upalpha \upbeta }(r)-1).$$

To distinguish between specific sites in a molecule, we exploited H/D isotopic substitution as their neutron scattering lengths differ both in magnitude and sign, being ${b}_{{{\rm{H}}}}=\,-3.739\,{{\rm{fm}}}$ and ${b}_{{{\rm{D}}}}=\,6.6671\,{{\rm{fm}}}$ for H and D respectively^61^. The partial radial distribution functions ${g}_{{{\upalpha }}{{\upbeta }}}\left(r\right)$ are related to the partial structure factors via Fourier transformation, as well as the running coordination number, ${n}_{{{\upalpha }}{{\upbeta }}}(r)$:

4 $${S}_{\upalpha \upbeta }(Q)=1+4\uppi \rho \int _{0}^{\infty }{r}^{2}[{g}_{\upalpha \upbeta }(r)-1]\frac{\sin (Qr)}{Qr}\,{{\rm{d}}}r,$$

5 $${n}_{\upalpha \upbeta }(r)=4\pi {\rho }_{\upbeta }\int _{{r}_{\min }}^{{r}_{\max }}{g}_{\upalpha \upbeta }(r)\,{r}^{2}{{\rm{d}}}r,$$

where *r*_min_ and *r*_max_ are the minimum and maximum integration distances between two species α and β, *ρ* is the atomic number density of the system, and *ρ*_β_ is the number density of species β. The ${g}_{{{\upalpha }}{{\upbeta }}}\left(r\right)$ represent the probability density of finding, by spherical averaging, an atom of species β at distance *r* (that is traditionally chosen as a minimum in the ${g}_{{{\upalpha }}{{\upbeta }}}\left(r\right)$) from an atom of species α, that is chosen as the origin of the reference system. The ${n}_{{{\upalpha }}{{\upbeta }}}(r)$, obtained by integrating the partial distribution functions ${g}_{{{\upalpha }}{{\upbeta }}}\left(r\right)$ over a sphere, is the average number of molecules present in a sphere of radius *r*.

The contributions to the integrand of Eq.(4) can be separated by Gaussian fitting, where the maximum is the average separation, and the area of the Gaussian is the number of molecules within that distance.

To go beyond the spherical average and extract important information about the different orientations, and therefore disentangle them from the spherical average, we can introduce the three-dimensional angular radial distribution functions (ARDFs) and SDFs, related to the probability density of finding a molecule at a given relative orientation from another one in the origin of the Cartesian system. The ARDF $g\left(r,\theta \right)$ is defined in the range θ ≤ π/2 as

6 $$g\left(r,\theta \right)=\,\frac{n(r,\theta )}{4{{\rm{\pi }}}{r}^{2}\,\rho \,\sin \theta \,\Delta r\,\Delta \theta }$$

where n(r,θ) is the number of molecules within a distance Δr and angle $\Delta \theta$^62^.

The SDFs are a harmonic representation of many-body correlation functions and allow us to picture in three dimensions the most probable location of a molecular species around another, as a function of distance and orientation^63–66^. To extrapolate these orientational maps, a set of Cartesian axes must be defined for each molecule (Suppl. Fig. 19).

D_2_O (99.9% D), H_7_/D_7_ aniline (98% D), H_6_/D_6_ phenol (99% D), and H_5_/D_5_ p-nitrophenol (98% D) were purchased from Sigma-Aldrich. Owing to the limited solubility of the chosen aromatics in water, the samples were made by mixing phenol, aniline and p-nitrophenol with water in air at the experimental molecular ratio of 1:200 ( ≈ 0.275 M) that correspond to a regime in which these aromatics are fully miscible in water at 70°C and that maximises the neutron scattering signal from the solute, allowing us to investigate any solute-solute association in solution^33,34,36^. Data for aniline and p-nitrophenol water solutions were acquired at the SANDALS diffractometer at the ISIS Neutron and Muon source (Didcot, UK) in a *Q* range of 0.08–50 Å^−1^, which translates to a real space sampling from the atomic (≈ 0.1 Å) to the nanoscale (≈ 80 Å)^67^. Samples were put in Ti_0.68_Zr_0.32_ null scattering cells and run for a minimum of ≈ 6 h each at 70 °C. Empty null-coherent scattering Ti_0.68_Zr_0.32_ cans and 3 mm vanadium flat plate were measured alongside the empty instrumental background. Data were corrected for multiple and inelastic scattering contributions, vanadium Bragg peaks subtraction, cell and empty instrument subtraction, and normalisation to absolute units (barn atom^−1^ Sr^−1^) using the GudrunN software^68^. Note that 1 barn = 10^−^^28^ m^2^. Data for phenol water solutions were acquired at the NIMROD diffractometer, in a *Q* range of 0.02–50 Å^−1^, and normalised in absolute units against 3 mm VNb null scattering plate.

### Empirical potential structure refinement simulations

Empirical potential structure refinement (EPSR) simulations allowed us to build a molecular ensemble of the solutions that is consistent with the experimental total neutron scattering data. As a traditional Monte Carlo method, it is a maximum entropy approach that avoids “over-structuring” of the liquids and solutions^68,69^. The EPSR method within the Dissolve software starts by using a combination of Monte Carlo and molecular dynamics steps, to model the total structure factors *F(Q)* and total pair distribution functions *G(r)* from initial Lennard-Jones plus Coulomb “seed” potentials (Fig. 1, Suppl. Fig. 23)^63,70^.

Once the initial simulation reaches equilibrium (≈ 2000 iterations) and energy stabilises with the reference potential (Suppl. Table 11), the refinement of the intermolecular potential is initiated to match the experimental interatomic distances. The method involves a small addition to the seed potential (the empirical potential) iteratively evaluated from the difference between measured and calculated structure factors to obtain the best match between the two patterns. When the best agreement is found, a large number (≈100,000) of configurations of the systems is acquired and the structural information extracted. In principle, the partial radial distribution functions could be extracted directly from the neutron data using a numerical Fourier transform inversion methodology. However, given that the number of independent pair correlations for a system of *i* non-equivalent atomic sites is $N=\frac{i(i+1)}{2}$, the inversion methodology is not uniquely constrained by the experimental data. Here, we further reduce the degrees of freedom by inputting molecular geometries and physicochemical properties of the systems, such as concentrations and densities.

The simulations were built by adding phenol, aniline, and p-nitrophenol to water in cubic simulation boxes of sides 106.8, 106.9, and 107.0 Å respectively, to reproduce the atomic number density of 0.1 atoms Å^-3^. The simulation boxes contain 200 molecules of substituted aromatic and 39,800 water molecules to reproduce the experimental molecular ratios of 1:200.

All the aromatic species were built in the LigParGen software provided by the Jorgensen group at Yale University using the OPLS-AA/1.14*CM1A force field (Suppl. Table 11), that is optimised to closely reproduce the free energies of hydration of organic molecules^71,72^. Water molecules were built in Dissolve (v1.8.0) and optimised using the SPC/F_W_ force field^73^. Water hydrogens, hydroxyl and amino hydrogens were set in Dissolve as exchangeable. The OH groups in phenol and p-nitrophenol, as well as the nitro group NO_2_ and the amino NH_2_ in p-nitrophenol and aniline are modelled free to rotate along the C-O and the C-N axes. Torsional parameters are listed in Supplementary Table 13. All the *g*_αβ_*(r)*, ARDFs, SDFs were extracted in the Dissolve GUI, and from the Dissolve trajectory file using the dlputils routines over *n* > 100,000 configurations, with uncertainties decreasing as ≈$\sqrt{\frac{1}{n}}$^62,63^.

### Reporting summary

Further information on research design is available in the Nature Portfolio Reporting Summary linked to this article.

## Supplementary information

Supplementary Information

Reporting Summary

Transparent Peer Review file

## Source data

Source Data

## Data Availability

The data that support the findings of this study, including unprocessed neutron data, are available from Figshare^74^ and from the corresponding authors upon request. Source data are provided with this paper.
