# Supplementary material for: Weak hydrogen bonding as the driver of aromatic hydration
Source: Nat Commun. 2026 Aug 11;17:9654. doi: 10.1038/s41467-026-76528-x (PMC13558607; doi:10.1038/s41467-026-76528-x)
Supplement: Supplementary file 1 [file 41467_2026_76528_MOESM1_ESM.pdf]

Supplementary Information for "Weak hydrogen bonding as the driver of aromatic hydration"

Camilla Di Mino,*[a,b] Daniel Bowron,[c] Michael A. Wilkinson,[a] Tristan G. Youngs, [c] Thomas F. Headen,[c] Andrea Sella,[d] and Neal T. Skipper*[a]

(a) Department of Physics and Astronomy, UCL, Gower Street, London WC1E 6BT, UK
(b) Physical and Theoretical Chemistry Laboratory, Department of Chemistry, University of Oxford, South Parks Road, Oxford OX1 3QZ, UK
(c) ISIS Neutron and Muon Source, RAL, Harwell Campus, Didcot OX11 0QX, UK
(d) Department of Chemistry, UCL, 20 Gordon Street, London WC1H 0AJ, UK

*Corresponding authors: camilla.dimino@chem.ox.ac.uk ; n.skipper@ucl.ac.uk

Supplementary Note 1: Experimental Details

Supplementary Table 1 Isotopically distinct mixtures. List of samples measured in the total neutron scattering experiment and relative concentrations. Note that pink (deuterium) and orange (hydrogen) identify the sites of isotopic exchange.

	**Phenol : water 1:200**	**Aniline : water 1:200**	**p-nitrophenol : water 1:200**
**1**	C_6D_5OD: D_2O	$C_6D_5ND_2$: D_2O	$C_6D_4NO_2OD$: D_2O
**2**	C_6D_5OD/C_6H_5OH : D_2O	$C_6D_5ND_2/C_6D_5ND_2$: D_2O	$C_6D_4NO_2OD/C_6H_4NO_2OH$: D_2O
**3**	C_6H_5OH : D_2O	$C_6H_5NH_2$: D_2O	$C_6H_4NO_2OH$: D_2O
**4**	C_6D_5OD : HDO	$C_6D_5ND_2$: HDO	$C_6D_4NO_2OD$: HDO
**5**	C_6D_5OD : H_2O	$C_6D_5ND_2$: H_2O	$C_6D_4NO_2OD$: H_2O
**6**	C_6H_5OH : H_2O	$C_6H_5NH_2$: H_2O	$C_6H_4NO_2OH$: H_2O

Supplementary Table 2 Phenol-water neutron weightings. Product of concentrations and scattering lengths for each pair correlation per isotopically distinct sample as defined from Eq. 2 for phenol/water at 1:200 molecular ratio. Red represents a low scattering weight, while green represents a high scattering weight.

**H** in **H**	**H** phenol	**O** phenol	**C** phenol	**H** water	**O** water
**H** phenol	0.00135	0.00070	0.00481	0.17959	0.13931
**O** phenol		0.00009	0.00124	0.02322	0.01801
**C** phenol			0.00427	0.15954	0.12375
**H** water				5.95647	4.62030
**O** water					3.58386

**D** in **D**	**D** phenol	**O** phenol	**C** phenol	**D** water	**O** water
**D** phenol	0.00431	0.00125	0.00858	0.57120	0.24844
**O** phenol		0.00009	0.00124	0.04141	0.01801
**C** phenol			0.00427	0.28453	0.12375
**D** water				18.94473	8.23986
**O** water					3.58386

H in D	H phenol	O phenol	C phenol	D water	O water
H phenol	0.00135	0.00070	0.00481	0.32029	0.13931
O phenol		0.00009	0.00124	0.04141	0.01801
C phenol			0.00427	0.28453	0.12375
D water				18.94473	8.23986
O water					3.58386

HD in D	HD phenol	O phenol	C phenol	D water	O water
HD phenol	0.00083	0.00055	0.00377	0.25091	0.10913
O phenol		0.00009	0.00124	0.04141	0.01801
C phenol			0.00427	0.28453	0.12375
D water				18.94473	8.23986
O water					3.58386

D in H	D phenol	O phenol	C phenol	H water	O water
D phenol	0.00431	0.00125	0.00858	0.32029	0.24844
O phenol		0.00009	0.00124	0.02322	0.01801
C phenol			0.00427	0.15954	0.12375
H water				5.95647	4.62030
O water					3.58386

D in HD	D phenol	O phenol	C phenol	HD water	O water
D phenol	0.00431	0.00125	0.00858	0.25091	0.10913
O phenol		0.00009	0.00124	0.01819	0.01801
C phenol			0.00427	0.12499	0.12375
HD water				3.65561	4.62030
O water					3.58386

Supplementary Table 3 Aniline-water neutron weightings. Product of concentrations and scattering lengths for each pair correlation per isotopically distinct sample as defined from Eq. 2 for aniline/water at 1:200 molecular ratio. Red represents a low scattering weight, while green represents a high scattering weight.

H in H	H aniline	N aniline	C aniline	H water	O water
H aniline	0.00184	0.00066	0.00280	0.10442	0.08100
N aniline		0.00024	0.00100	0.03737	0.02898
C aniline			0.00426	0.02650	0.12335
H water				5.93699	4.60519
O water					3.57214

D in D	D aniline	N aniline	C aniline	D water	O water
D aniline	0.00584	0.00117	0.00499	0.33211	0.14445
N aniline		0.00024	0.00100	0.06664	0.02898
C aniline			0.00426	0.04727	0.12335
D water				18.88277	4.60519
O water					3.57214

H in D	H aniline	N aniline	C aniline	D water	O water
H aniline	0.00184	0.00066	0.00280	0.18622	0.08100
N aniline		0.00024	0.00100	0.06664	0.02898
C aniline			0.00426	0.04727	0.12335
D water				18.88277	8.21291
O water					3.57214

HD in D	HD aniline	N aniline	C aniline	D water	O water
HD aniline	0.00028	0.00026	0.00110	0.07294	0.03173
N aniline		0.00024	0.00100	0.06664	0.02898
C aniline			0.00426	0.04727	0.12335
D water				18.88277	8.21291
O water					3.57214

D in H	D aniline	N aniline	C aniline	H water	O water
D aniline	0.00584	0.00117	0.00499	0.18622	0.14445
N aniline		0.00024	0.00100	0.03737	0.02898
C aniline			0.00426	0.02650	0.12335
H water				5.93699	4.60519
O water					3.57214

D in HD	D aniline	N aniline	C aniline	HD water	O water
D aniline	0.00584	0.00066	0.00499	0.07294	0.14445
N aniline		0.00024	0.00100	0.01464	0.02898
C aniline			0.00426	0.01038	0.12335
HD water				0.91091	1.80386
O water					3.57214

Supplementary Table 4 P-nitrophenol-water neutron weightings. Product of concentrations and scattering lengths for each pair correlation per isotopically distinct sample as defined from Eq. 2 for p-nitrophenol/water at 1:200 molecular ratio. Red represents a low scattering weight, while green represents a high scattering weight.

H in H	H nitro	O nitro	C nitro	N nitro	H water	O water
H nitro	0.00093	0.00174	0.00398	0.00094	0.14868	0.11533
O nitro		0.00081	0.00371	0.00044	0.06920	0.05368
C nitro			0.00425	0.00100	0.15850	0.12295
N nitro				0.00023	0.03724	0.02889
H water					5.91760	4.59015
O water						3.56048

D in D	D nitro	O nitro	C nitro	N nitro	D water	O water
D nitro	0.00297	0.00310	0.00710	0.00167	0.47289	0.20568
O nitro		0.00081	0.00371	0.00044	0.12341	0.05368
C nitro			0.00425	0.00100	0.28267	0.12295
N nitro				0.00023	0.06642	0.02889
D water					18.82111	8.18610
O water						3.56048

H in D	H nitro	O nitro	C nitro	N nitro	D water	O water
H nitro	0.00093	0.00174	0.00398	0.00094	0.26516	0.11533
O nitro		0.00081	0.00371	0.00044	0.12341	0.05368
C nitro			0.00425	0.00100	0.28267	0.12295
N nitro				0.00023	0.06642	0.02889
D water					18.82111	8.18610
O water						3.56048

HD in D	HD nitro	O nitro	C nitro	N nitro	D water	O water
HD nitro	0.00014	0.00068	0.00156	0.00037	0.10387	0.04518
O nitro		0.00081	0.00371	0.00044	0.12341	0.05368
C nitro			0.00425	0.00100	0.28267	0.12295
N nitro				0.00023	0.06642	0.02889
D water					18.82111	8.18610
O water						3.56048

D in H	D nitro	O nitro	C nitro	N nitro	H water	O water
D nitro	0.00297	0.00310	0.00710	0.00167	0.26516	0.20568
O nitro		0.00081	0.00371	0.00044	0.06920	0.05368
C nitro			0.00425	0.00100	0.15850	0.12295
N nitro				0.00023	0.06642	0.02889
H water					5.91760	4.59015
O water						3.56048

D in HD	D nitro	O nitro	C nitro	N nitro	HD water	O water
D nitro	0.00297	0.00310	0.00710	0.00167	0.10387	0.04518
O nitro		0.00081	0.00371	0.00044	0.02711	0.05368
C nitro			0.00425	0.00100	0.06209	0.12295
N nitro				0.00023	0.01459	0.02889
HD water					0.90794	4.59015
O water						3.56048

Supplementary Note 2: Neutron Scattering Data and Uncertainties

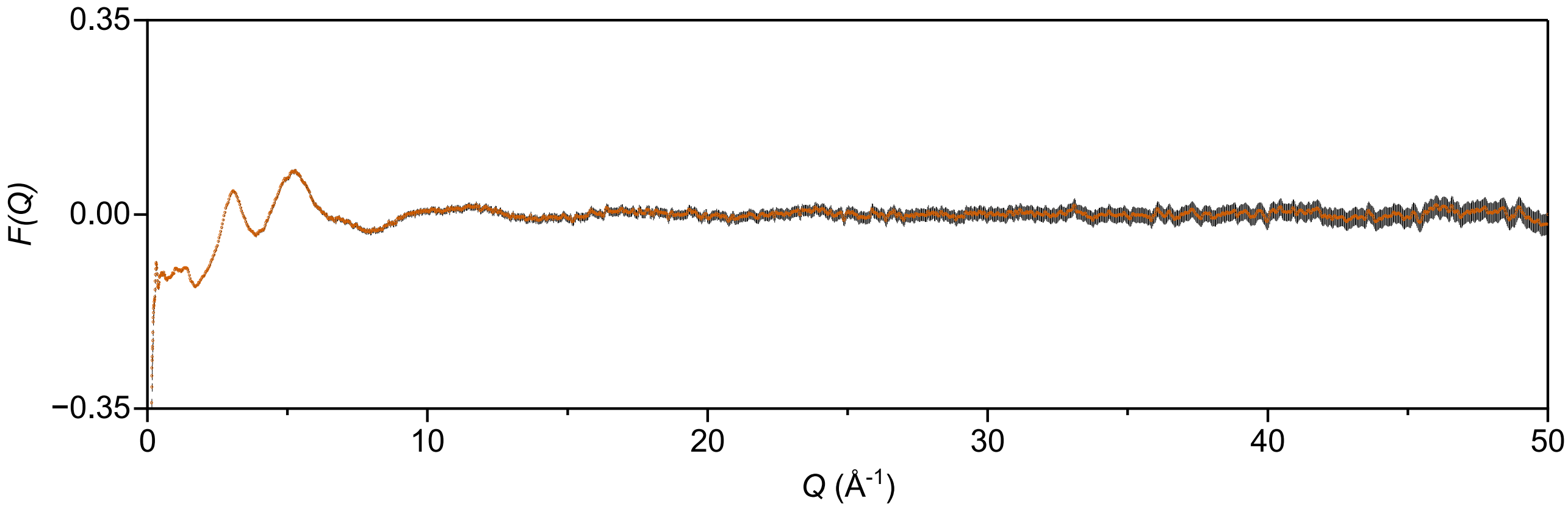

Supplementary Figure 1 Full range neutron data and uncertainties. Neutron total scattering pattern for H_6 Phenol in H_2O (orange circles) plotted with experimental uncertainties (black vertical bars).

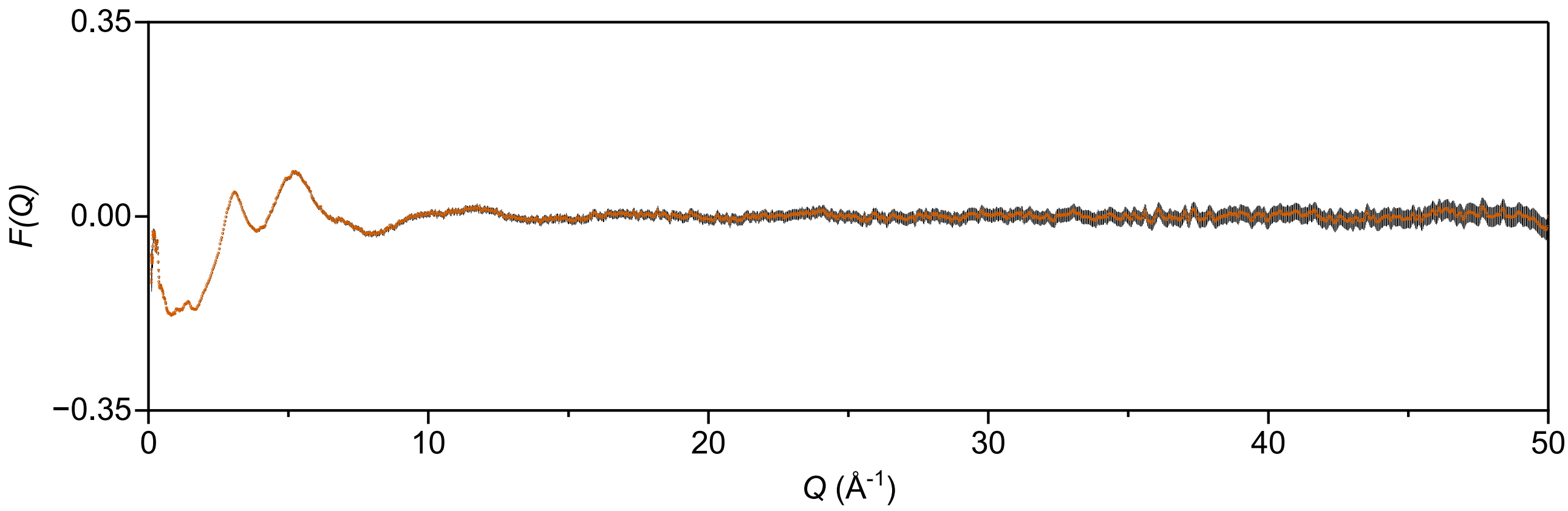

Supplementary Figure 2 Full range neutron data and uncertainties. Neutron total scattering pattern for D_6 Phenol in H_2O (orange circles) plotted with experimental uncertainties (black vertical bars).

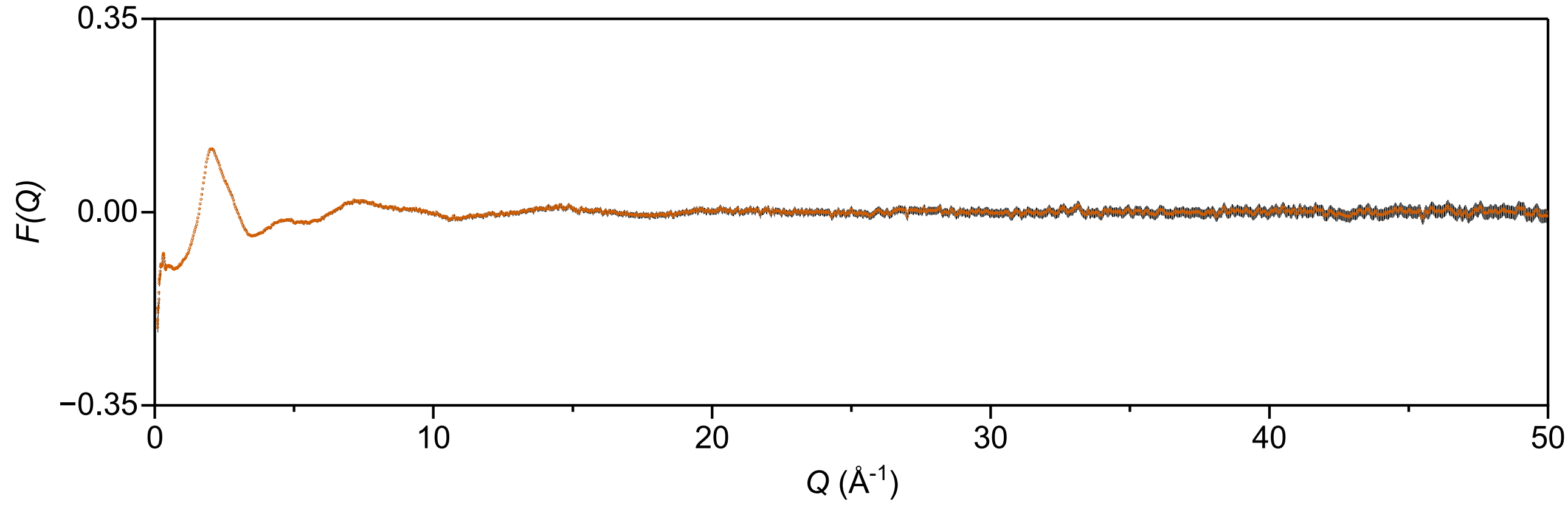

Supplementary Figure 3 Full range neutron data and uncertainties. Neutron total scattering pattern for D_6 Phenol in HDO (orange circles) plotted with experimental uncertainties (black vertical bars).

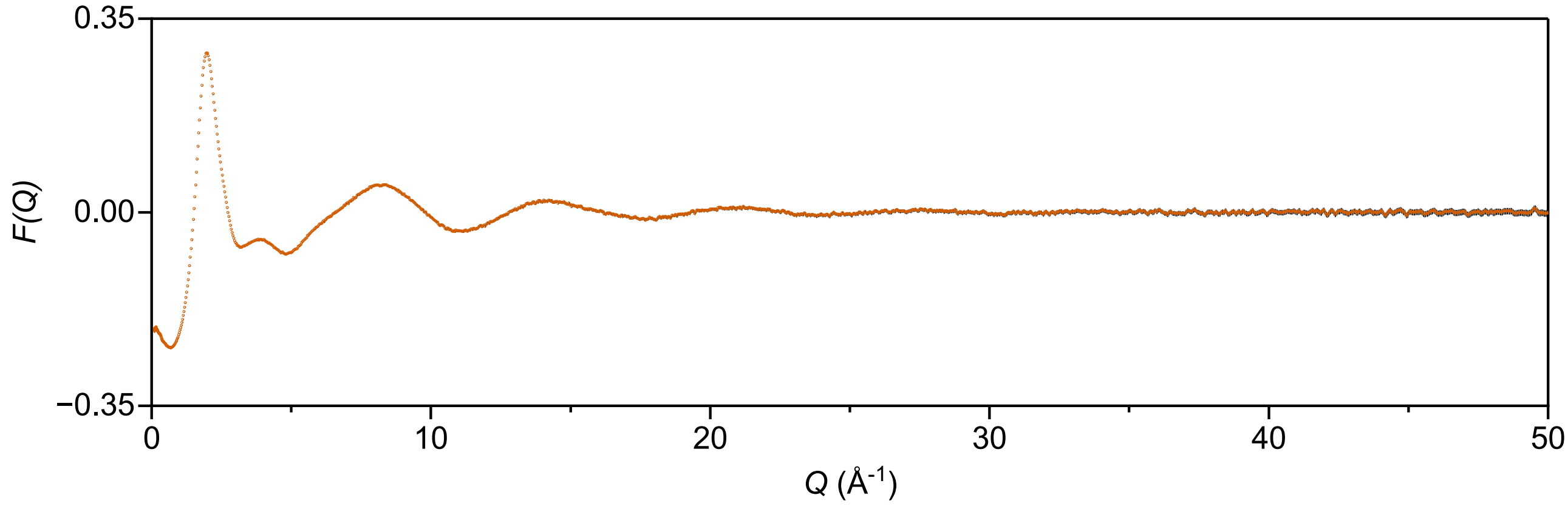

Supplementary Figure 4 Full range neutron data and uncertainties. Neutron total scattering pattern for H_6 Phenol in D_2O (orange circles) plotted with experimental uncertainties (black vertical bars).

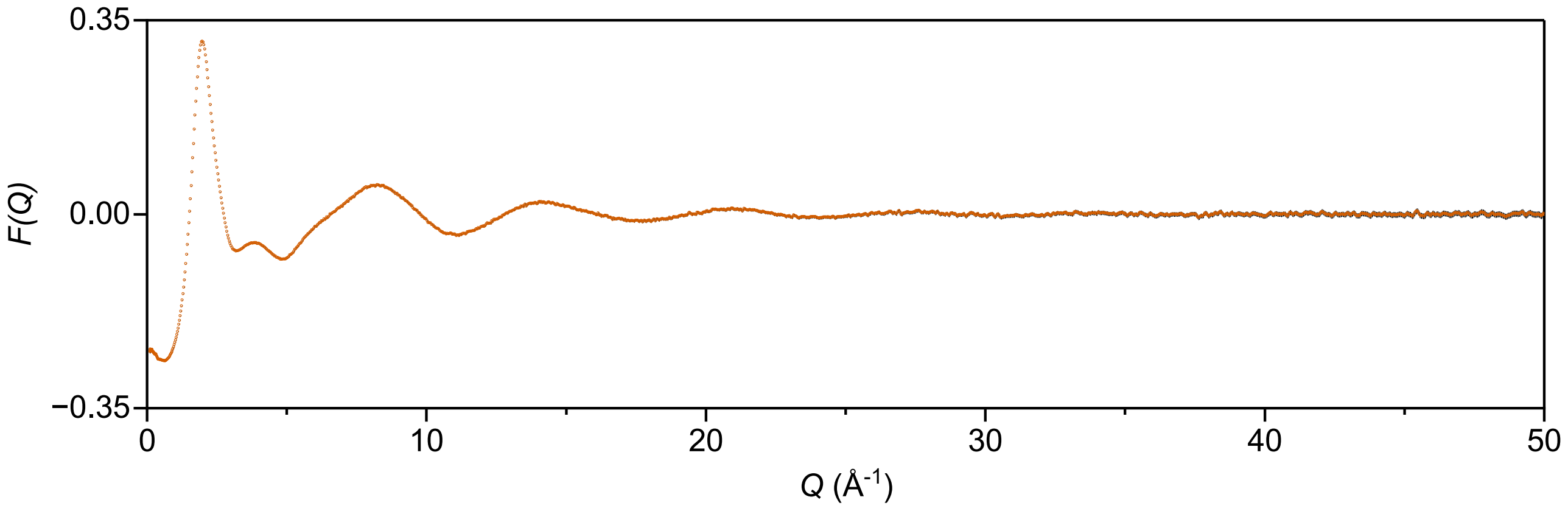

Supplementary Figure 5 Full range neutron data and uncertainties. Neutron total scattering pattern for (HD)$_6$ Phenol in D_2O (orange circles) plotted with experimental uncertainties (black vertical bars).

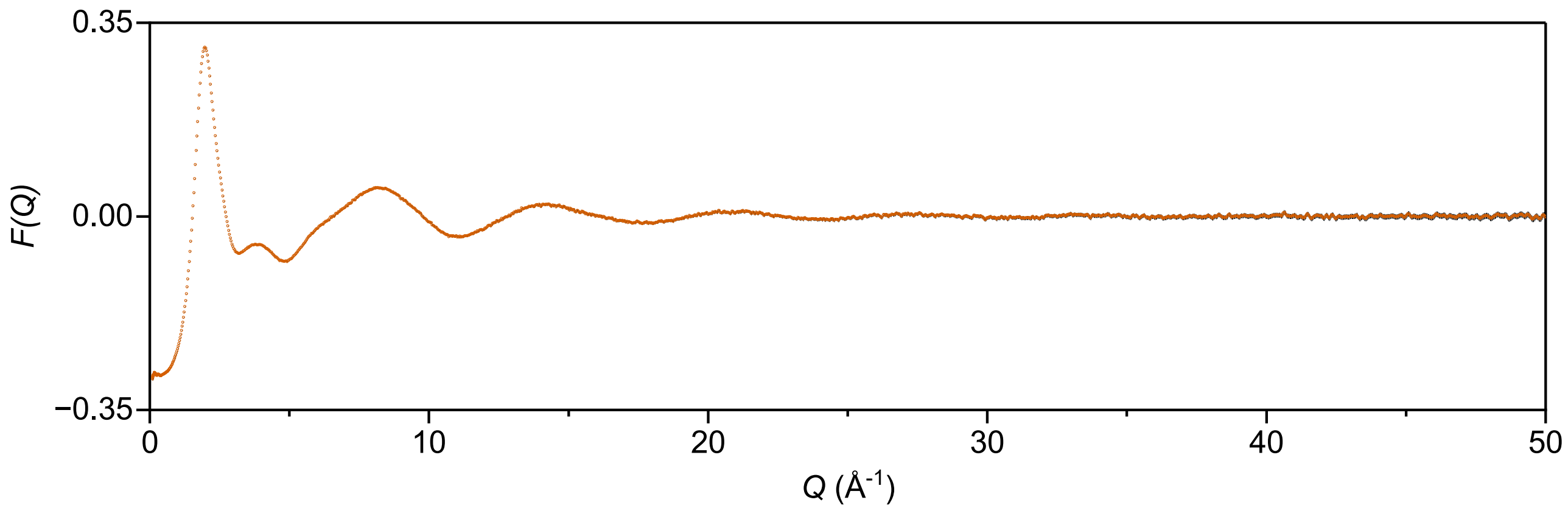

Supplementary Figure 6 Full range neutron data and uncertainties. Neutron total scattering pattern for D_6 Phenol in D_2O (orange circles) plotted with experimental uncertainties (black vertical bars).

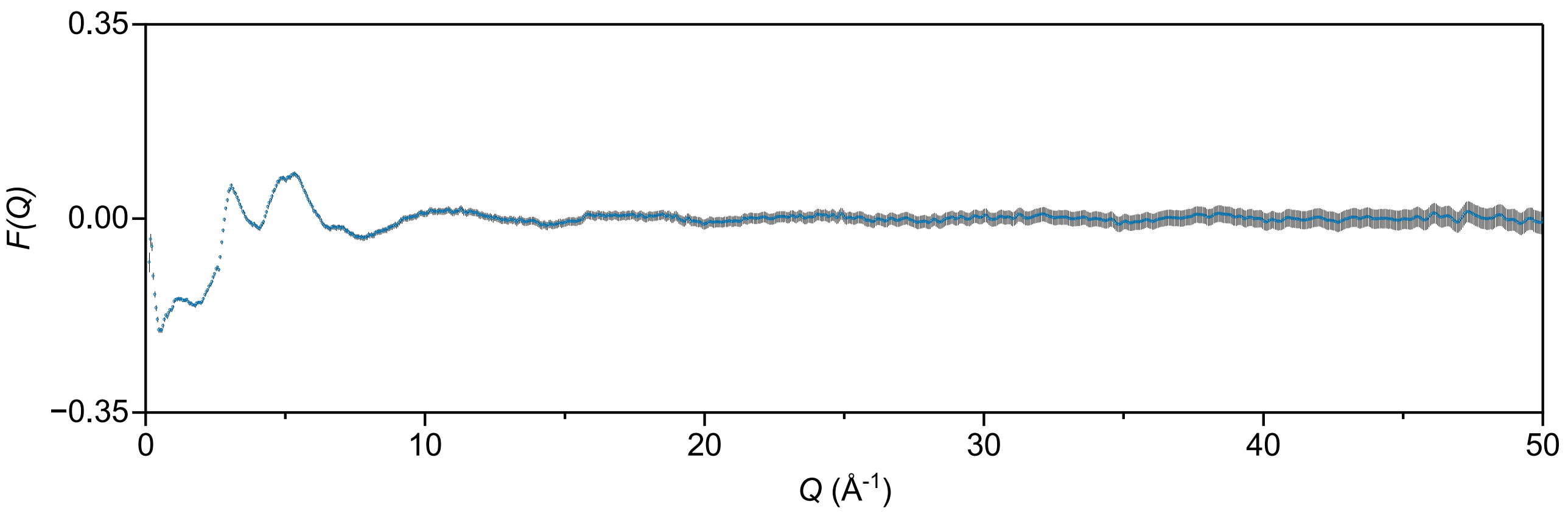

Supplementary Figure 7 Full range neutron data and uncertainties. Neutron total scattering pattern for H_7 Aniline in H_2O (blue circles) plotted with experimental uncertainties (black vertical bars).

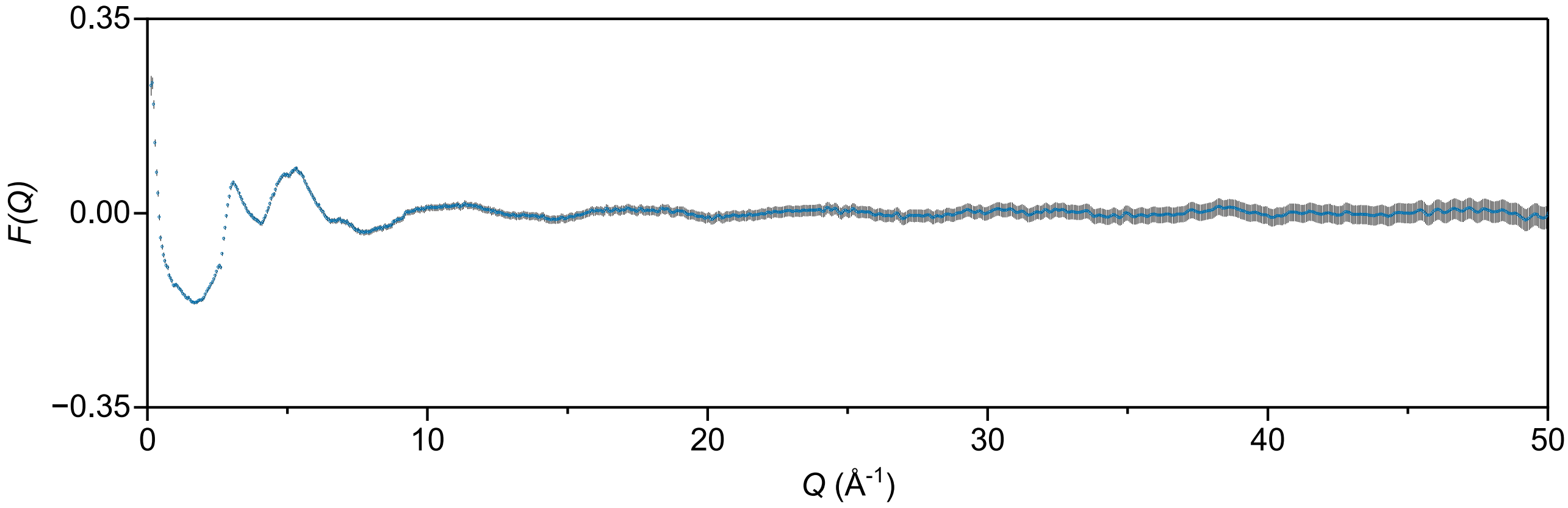

Supplementary Figure 8 Full range neutron data and uncertainties. Neutron total scattering pattern for D_7 Aniline in H_2O (blue circles) plotted with experimental uncertainties (black vertical bars).

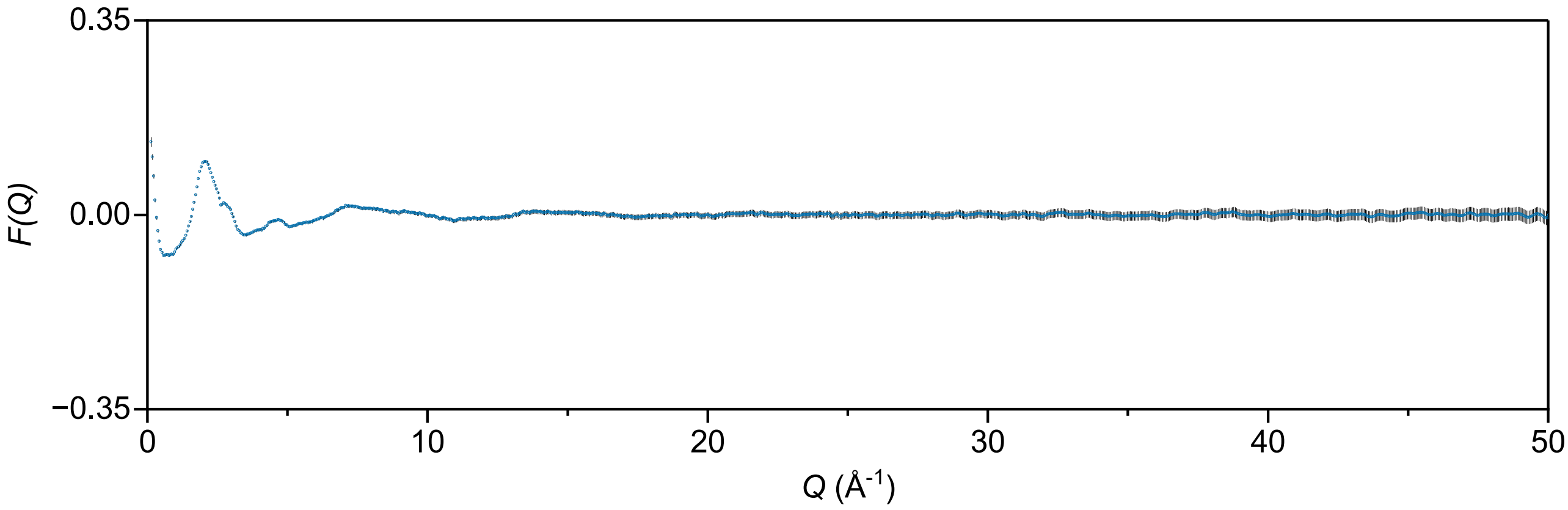

Supplementary Figure 9 Full range neutron data and uncertainties. Neutron total scattering pattern for D_7 Aniline in HDO (blue circles) plotted with experimental uncertainties (black vertical bars).

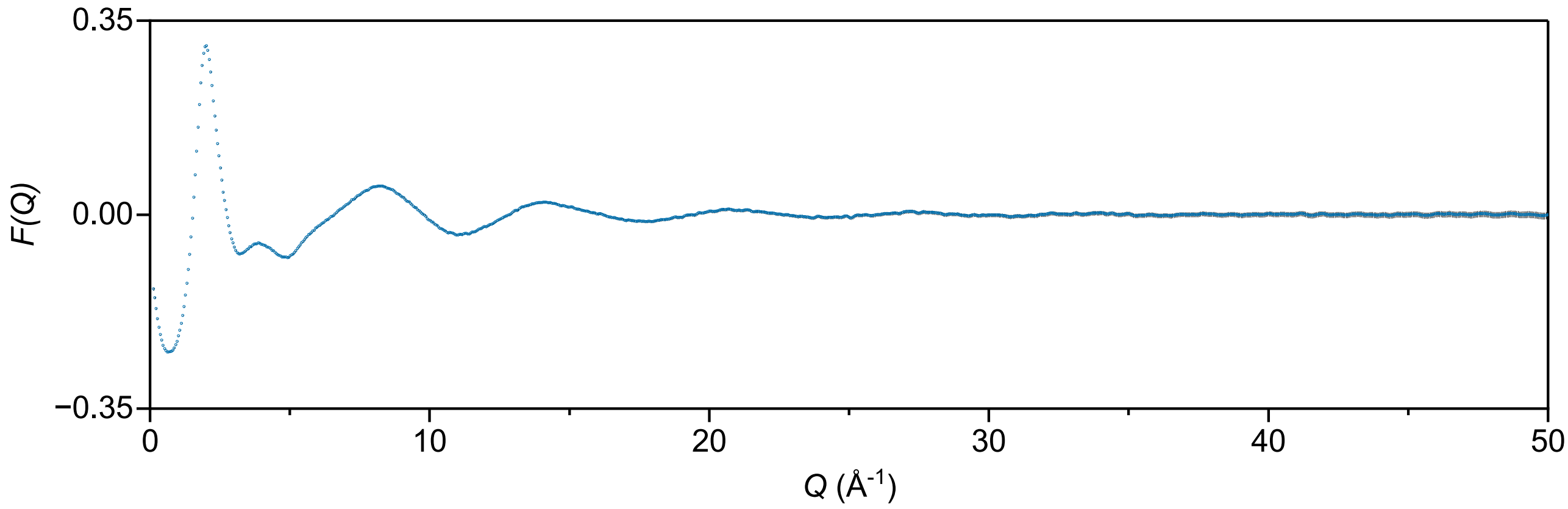

Supplementary Figure 10 Full range neutron data and uncertainties. Neutron total scattering pattern for H_7 Aniline in D_2O (blue circles) plotted with experimental uncertainties (black vertical bars).

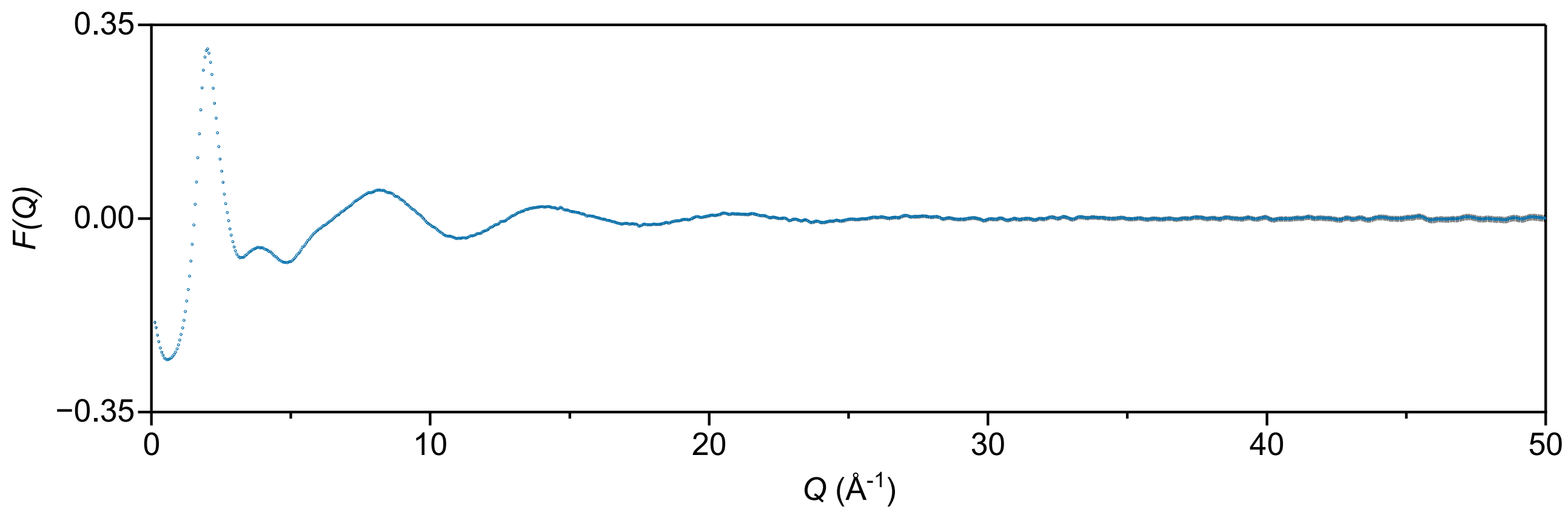

Supplementary Figure 11 Full range neutron data and uncertainties. Neutron total scattering pattern for (**HD**)$_7$ Aniline in **D**$_2$O (blue circles) plotted with experimental uncertainties (black vertical bars).

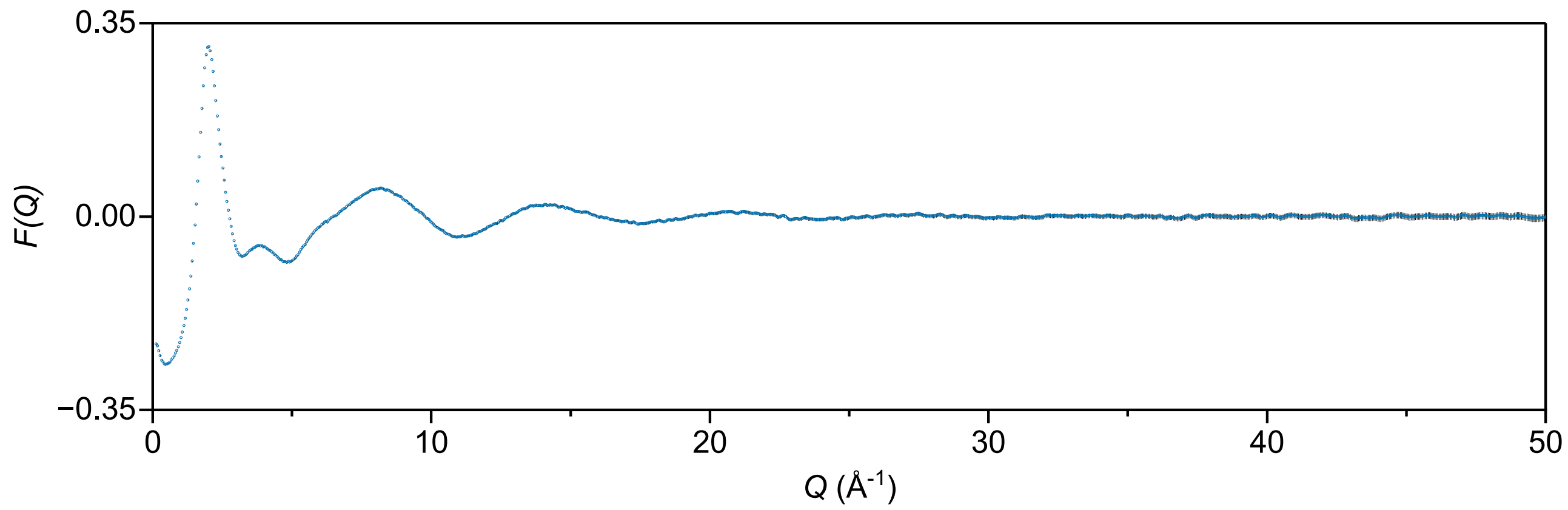

Supplementary Figure 12 Full range neutron data and uncertainties. Neutron total scattering pattern for **D**$_7$ Aniline in **H**$_2$O (blue circles) plotted with experimental uncertainties (black vertical bars).

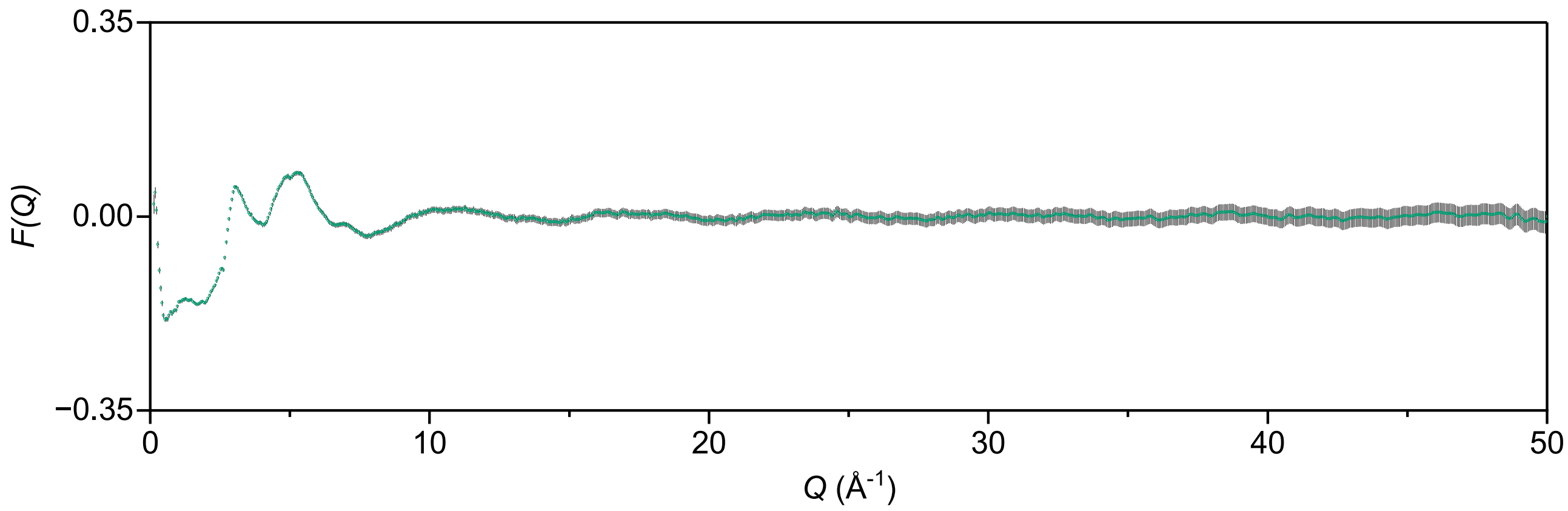

Supplementary Figure 13 Full range neutron data and uncertainties. Neutron total scattering pattern for **H**$_5$ P-nitrophenol in **H**$_2$O (green circles) plotted with experimental uncertainties (black vertical bars).

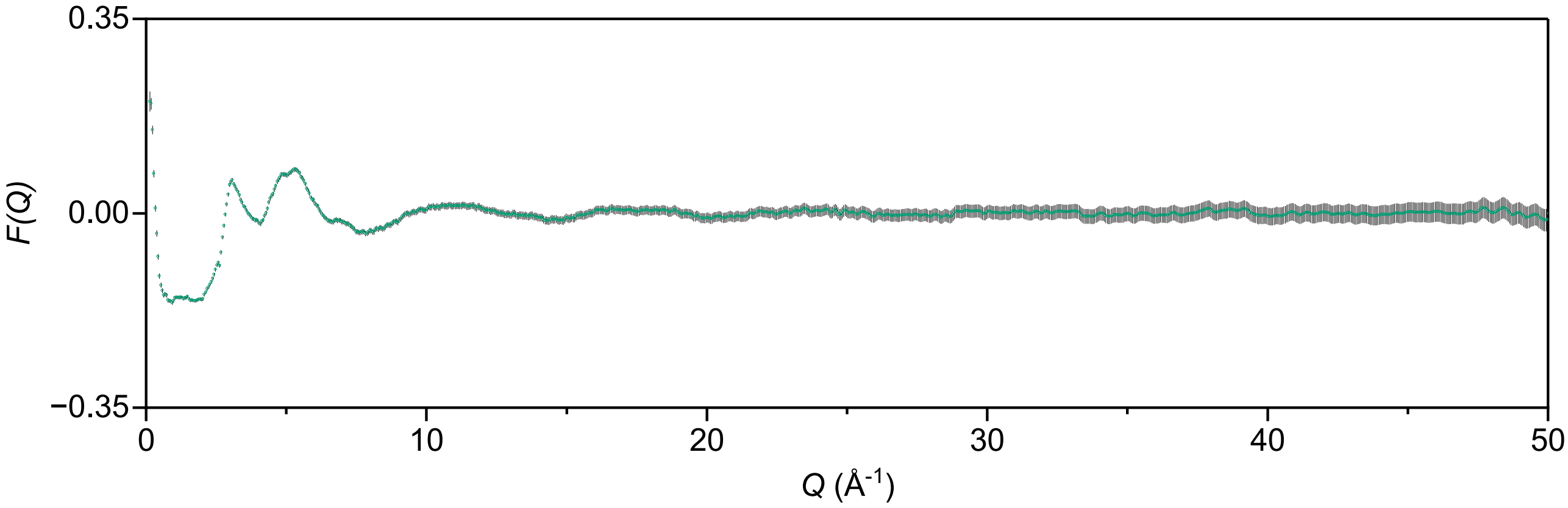

Supplementary Figure 14 Full range neutron data and uncertainties. Neutron total scattering pattern for D_5 P-nitrophenol in H_2O (green circles) plotted with experimental uncertainties (black vertical bars).

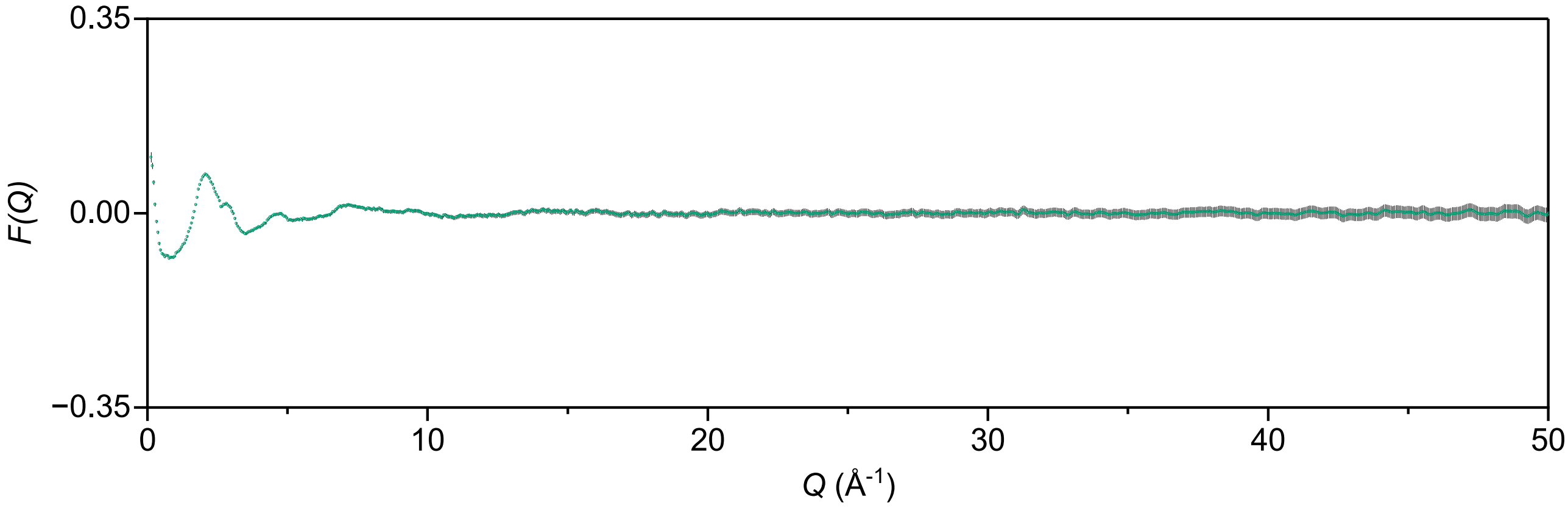

Supplementary Figure 15 Full range neutron data and uncertainties. Neutron total scattering pattern for D_5 P-nitrophenol in HDO (green circles) plotted with experimental uncertainties (black vertical bars).

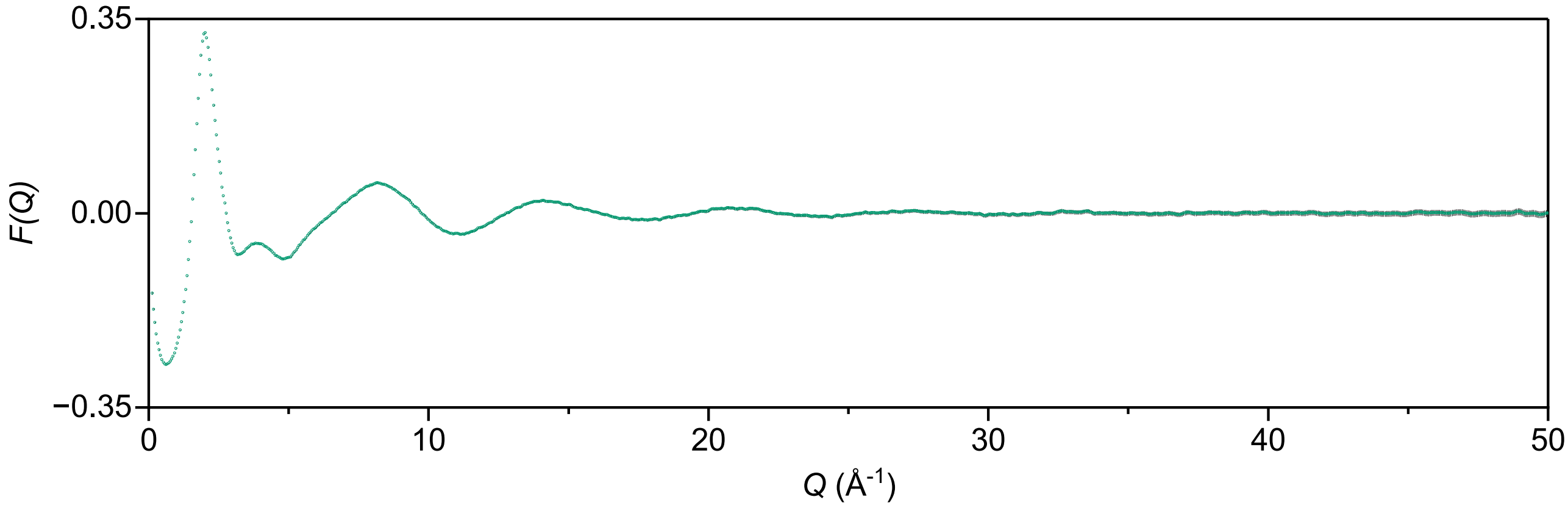

Supplementary Figure 16 Full range neutron data and uncertainties. Neutron total scattering pattern for H_5 P-nitrophenol in D_2O (green circles) plotted with experimental uncertainties (black vertical bars).

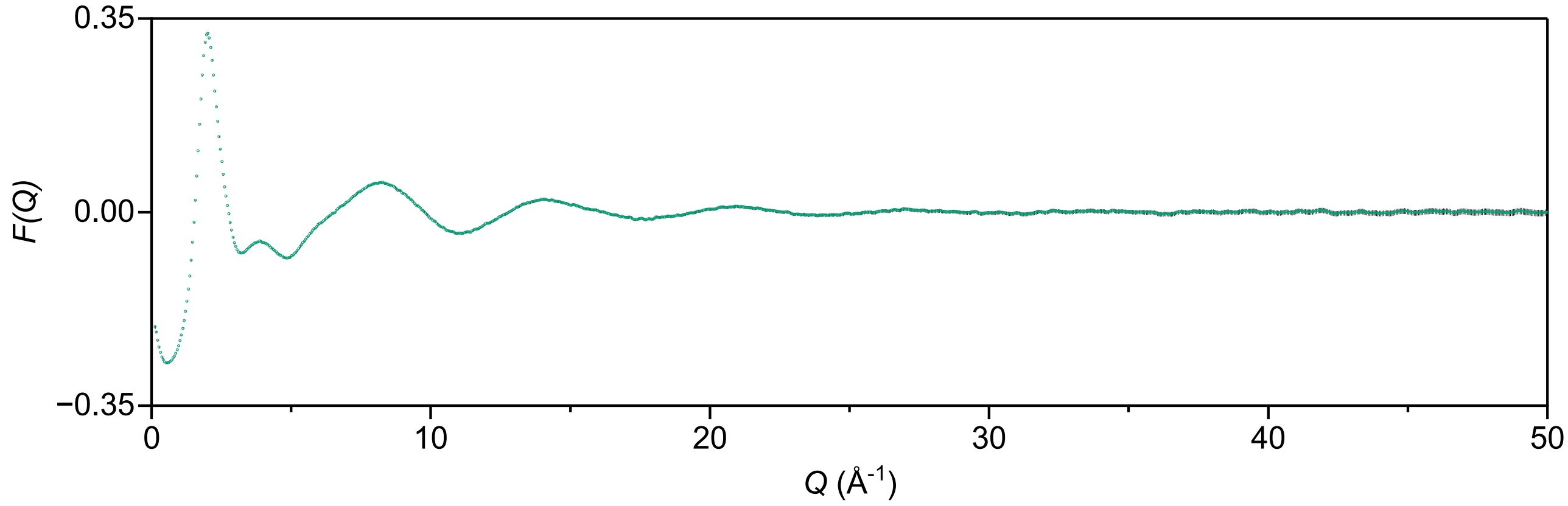

Supplementary Figure 17 Full range neutron data and uncertainties. Neutron total scattering pattern for (HD)$_5$ P-nitrophenol in D_2O (green circles) plotted with experimental uncertainties (black vertical bars).

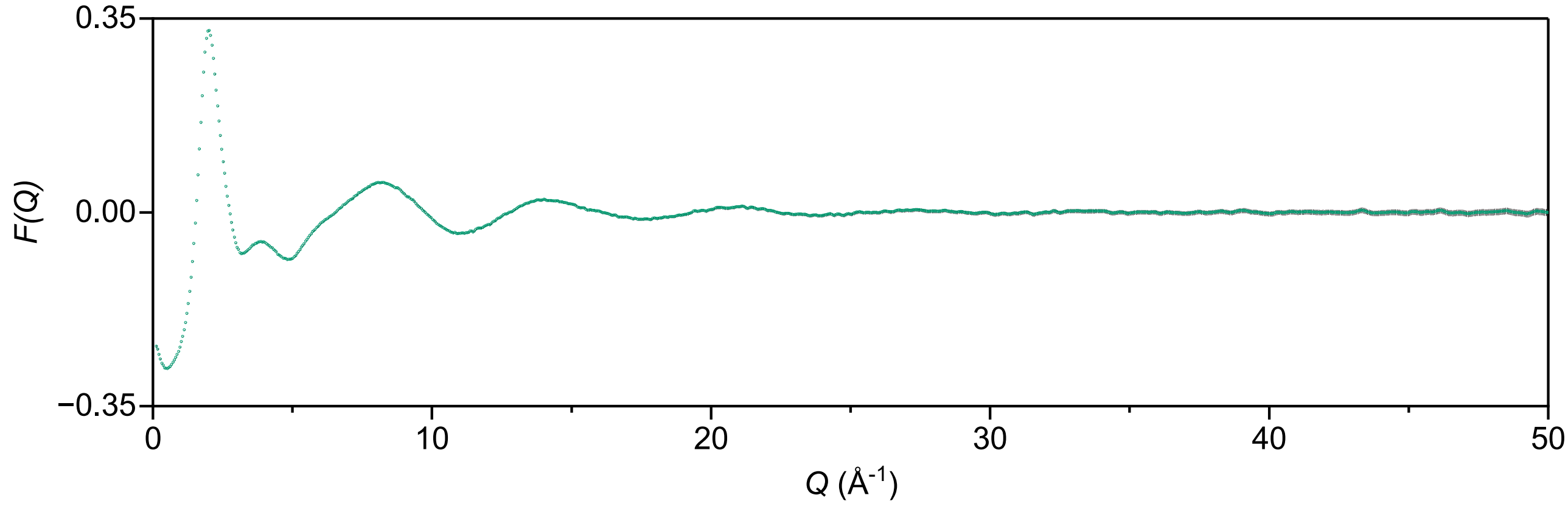

Supplementary Figure 18 Full range neutron data and uncertainties. Neutron total scattering pattern for D_5 P-nitrophenol in D_2O (green circles) plotted with experimental uncertainties (black vertical bars).

Supplementary Note 3: Coordination Numbers

Supplementary Table 5 Average coordination numbers relative to molecule overall solvation. Centre-of-Geometry CoG-CoG coordination numbers for aromatic-aromatic and aromatic-water. Definition of CoG in Figure 1. Theoretical coordination numbers were evaluated considering the number of water molecules (spheres of radius 0.95 Å) within a sphere of radius 2.82, 2.91, and 3.27 Å for phenol, aniline, and p-nitrophenol. Radii were evaluated as half of the distance between the two furthest atoms in the plane of the aromatic in the Avogadro software.[1] Note the theoretical values increase for p-nitrophenol that is the opposite trend we observe from our analysis.

	First Peak Position (Å)	Second Peak Position (Å)	Integration Limits (Å)	Coordination Number	Theoretical Coordination Number
**Phenol – Phenol**	5.60	9.45	8.00	0.45	
**Aniline – Aniline**	5.70	9.60	8.00	0.30	
**p-nitrophenol– p-nitrophenol**	3.90	7.20	6.40	0.73	
**Phenol – Water**	3.00	4.95	3.5/6.5	1.43 / 33.1	23.4
**Aniline – Water**	3.20	5.10	3.5/6.5	1.45 / 33.2	25.8
**p-nitrophenol– Water**	-	5.06	3.5*/6.5	0.54 / 29.4	36.6

Supplementary Table 6 Average coordination numbers relative to molecule single sites solvation. Site-site coordination numbers for hydroxyl, amine, and nitro groups in solute-solute contacts. Note that the coordination number depends on the number of sites, $n_{\alpha\beta} \neq n_{\beta\alpha}$

Solute Functional Group – Solvent	First Peak Position (Å)	Second Peak Position (Å)	Integration Limits (Å)	Coordination Number
**Phenol O – Phenol H**	2.05	3.50	2.50*	0.003
**Aniline N – Aniline H**	2.10	4.60	2.50*	0.001
**Aniline H – Aniline N**	2.10	4.60	2.50*	0.001
**p-nitrophenol O(H) – p-nitrophenol H(O)**	2.05	4.40	2.50*	0.002
**p-nitrophenol H(O) – p-nitrophenol O(N)**	1.80	4.10	2.50*	0.010
**p-nitrophenol O(N) – p-nitrophenol H(O)**	1.80	4.10	2.50*	0.010

Supplementary Table 7 Average coordination numbers relative to molecule single sites solvation. Site-site coordination numbers for hydroxyl, amine, and nitro group and water.

Solute Functional Group – Solvent	First Peak Position (Å)	Second Peak Position (Å)	Integration Limits (Å)	Coordination Number
**Phenol O – Water H**	2.00	3.38	2.50*	1.10
**Phenol H – Water O**	1.79	3.55	2.50*	0.98
**Aniline N – Water H**	1.95	3.50	2.50*	0.88
**Aniline H – Water O**	2.00	3.37	2.50*	0.66
**p-nitrophenol O (H) – Water H**	2.05	3.38	2.50*	0.76
**p-nitrophenol H – Water O**	1.77	3.63	2.50*	1.00
**p-nitrophenol O (N) – Water H**	1.85	3.90	2.50*	1.40

Supplementary Table 8 Average coordination numbers relative to molecule single sites solvation. Centre-of-the-ring CoR-H_w and CoR-O_w coordination numbers for aromatic-water. Definition of CoR in Figure 1. Integration limit is chosen as first minimum in the partial $g_{CoR\text{-}Hw}(r)$ and $g_{CoR\text{-}Ow}(r)$ and kept consistent for the three aromatics for comparison. Note that there is no pre-peak in the p-nitrophenol $g_{CoR\text{-}Ow}(r)$ and therefore separate fitting is done in Figure 4 and Supplementary Table 4.

Solute – Solvent Individual Sites	First Peak Position (Å)	Second Peak Position (Å)	Integration Limit (Å)	Coordination Number
**Phenol CoR – Water H**	2.29	4.90	2.80	1.20
**Phenol CoR – Water O**	3.22	4.85	3.80	1.80
**Aniline CoR – Water H**	2.37	5.02	2.80	0.97
**Aniline CoR – Water O**	3.32	4.90	3.80	1.45
**p-nitrophenol CoR – Water H**	2.50	5.17	2.80	0.24
**p-nitrophenol CoR – Water O**	-	4.77	3.80*	0.97

Supplementary Table 9 Occurrence of individual site contacts. Breakdown of different types of contacts. Note that the three aromatics are fully solvated by water, and that in p-nitrophenol the OH···CoR are remarkably reduced.

	0 contacts	1 contact	2 contacts	3 contacts
**Phenol – Phenol**	64%	33%	3%	–
**Aniline – Aniline**	71%	22%	6%	1%
**P-Nitrophenol – P-Nitrophenol**	52%	33%	12%	3%
**Phenol CoR – Hw**	17%	50%	33%	-
**Aniline CoR - Hw**	28%	48%	24%	-
**P-nitrophenol CoR-Hw**	77%	21%	2%	-

Supplementary Table 10 Breakdown of peak fitting. Peak positions and coordination numbers obtained by Gaussian fitting of the $n_{CoR\text{-}Hw}(r)$, $n_{CoR\text{-}Ow}(r)$, and $n_{N\text{-}Ow}(r)$. Note that the coordination numbers are comparable to the ones of Supplementary Table 3.

	1st Peak Position (Å)	Coordination Number	2nd Peak Position (Å)	Coordination Number	3rd Peak Position (Å)	Coordination Number
**p-nitrophenol CoR – Water H**	2.62	0.24	3.73	2.50	5.18	35.2
**p-nitrophenol CoR – Water O**	3.44	0.53	3.96	1.90	4.68	6.40
**p-nitrophenol N – Water O**	3.35	0.73	3.82	3.20	4.38	5.20

Supplementary Note 4: Solute-Solute Relative Orientation

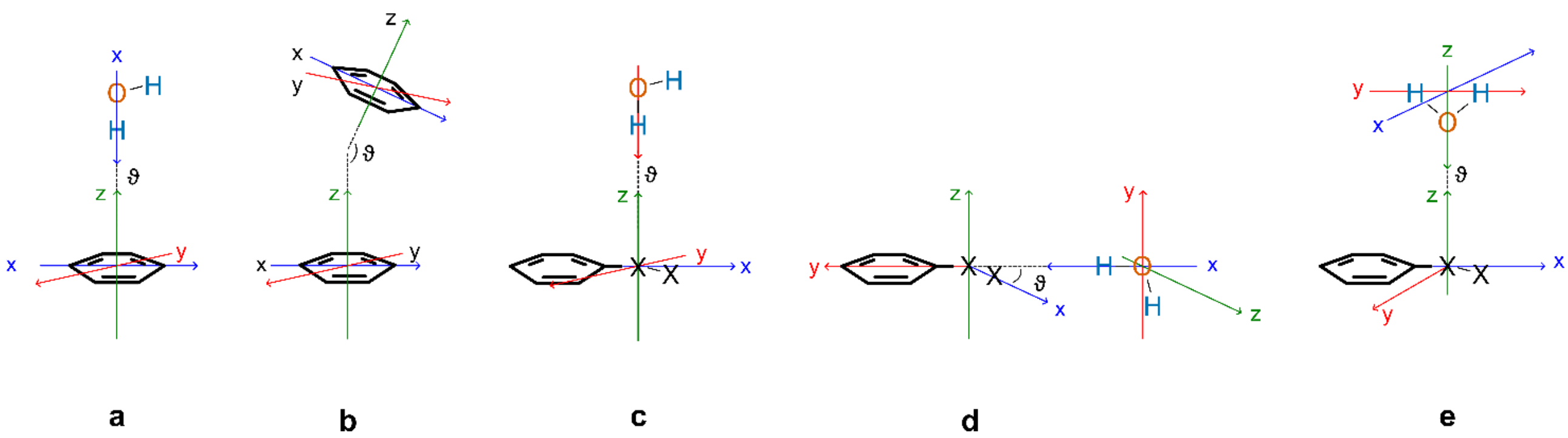

Supplementary Figure 19 Molecular orientations. Definition of possible molecular orientations between substituted aromatics and water. **a** Ring-water; **b** Ring-ring; **c,d,e** Substituents-water;

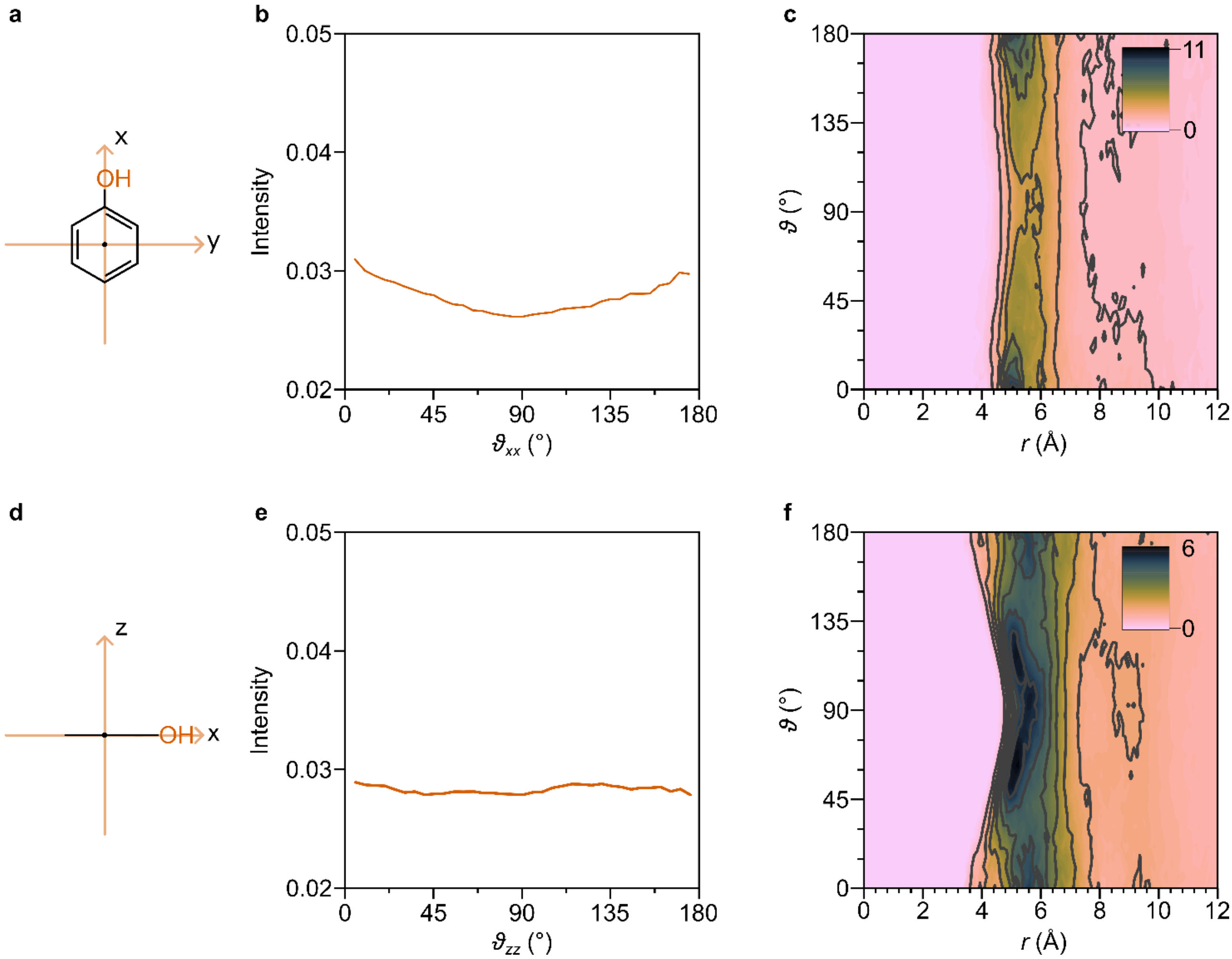

Supplementary Figure 20 Phenol-phenol relative orientation. a,d Phenol axes definition; **b,e** Phenol-phenol relative orientation as a function of the angle ϑ between the *xx* and *zz*; **c,f** Phenol-phenol relative orientation as a function of the angle ϑ between the *xx* and *zz*, as a function of the relative distance between the origins. Note the absence of sharp features as an indication of loose, non-directional association.

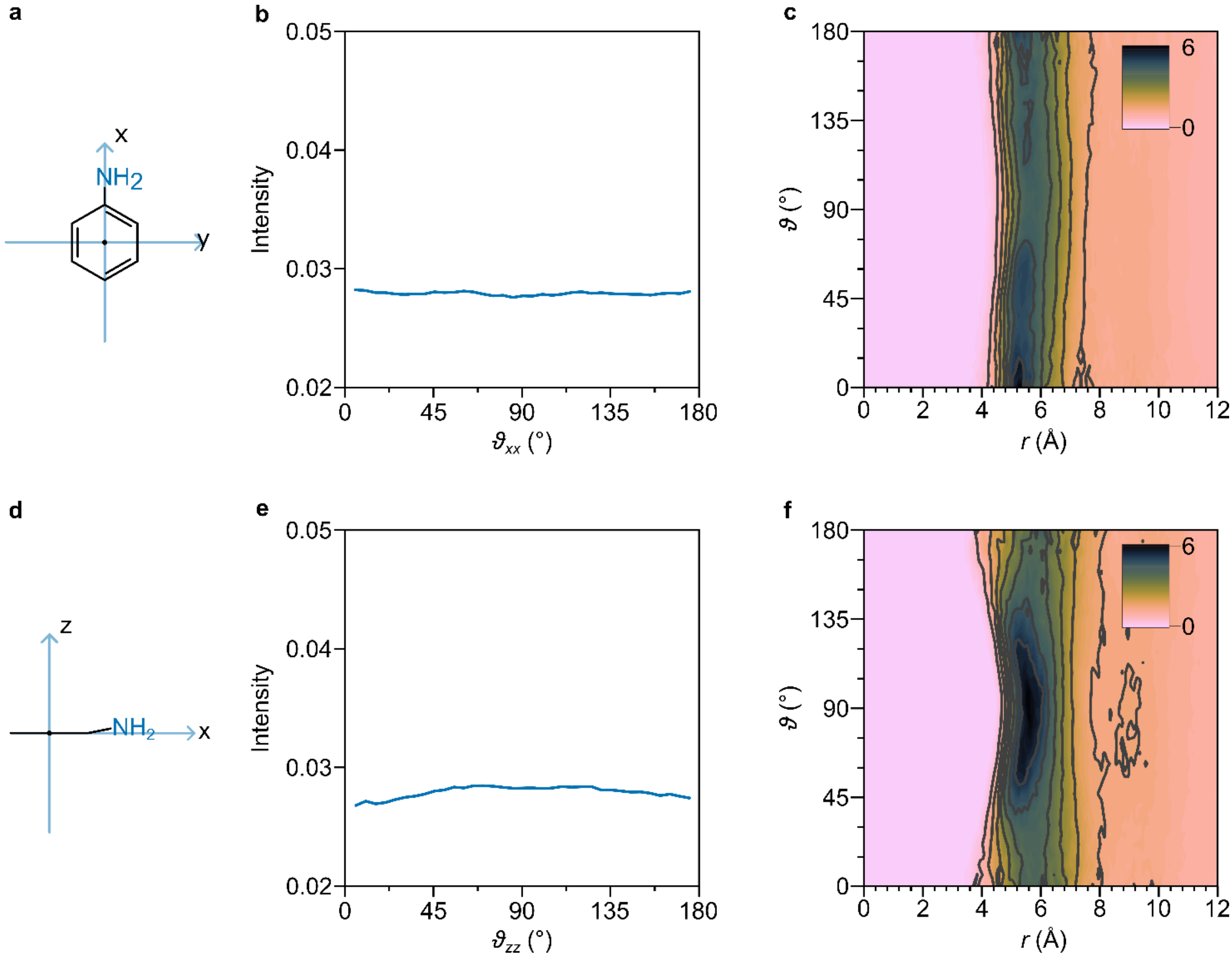

Supplementary Figure 21 Aniline-aniline relative orientation. a,d Aniline axes definition; **b,e** Aniline-aniline relative orientation as a function of the angle ϑ between the *xx* and *zz*; **c,f** Aniline-aniline relative orientation as a function of the angle ϑ between the *xx* and *zz*, as a function of the relative distance between the origins. Note the absence of sharp features as an indication of loose, non-directional association.

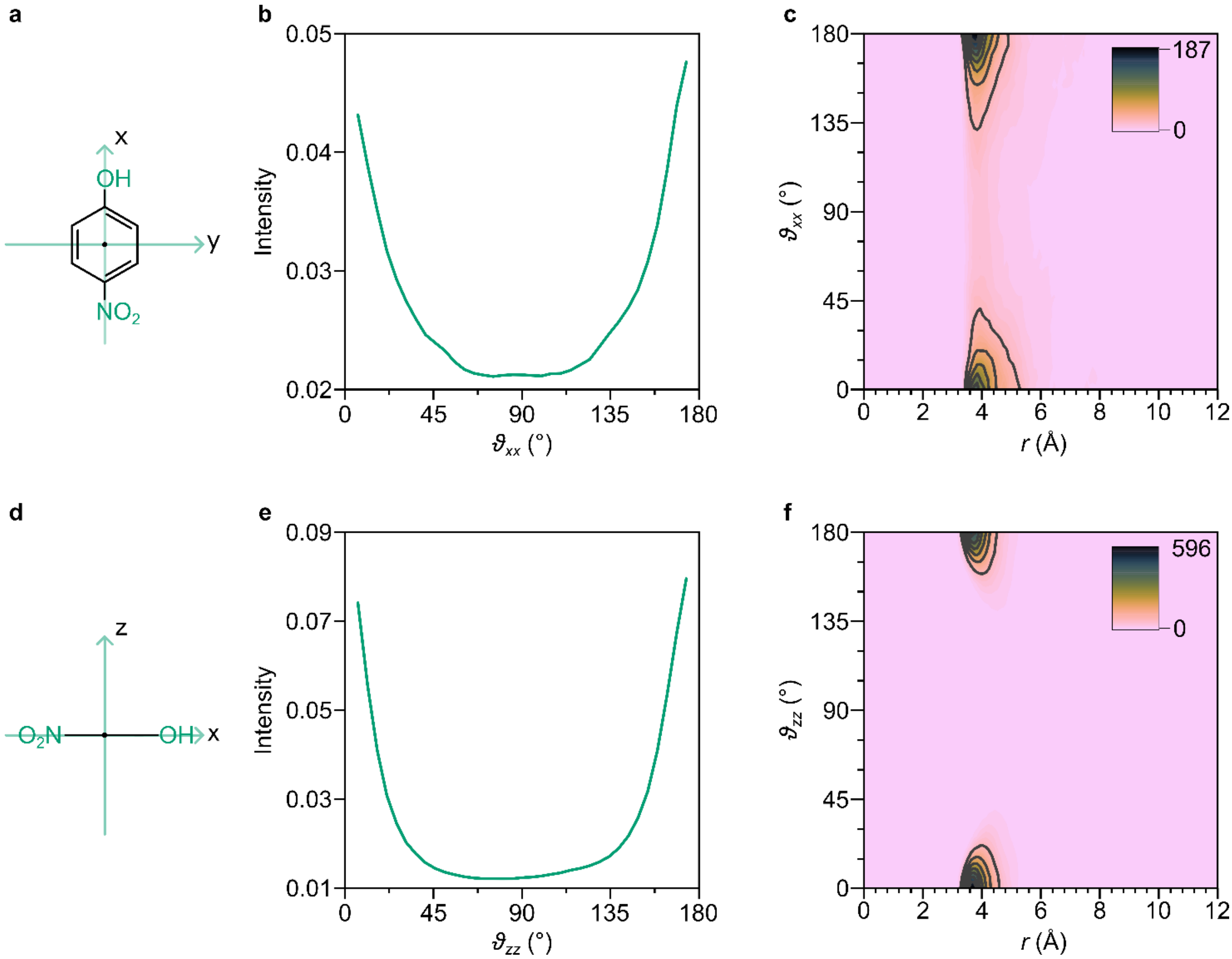

Supplementary Figure 22 a,d p-nitrophenol axes definition; **b,e** p-nitrophenol-p-nitrophenol relative orientation as a function of the angle ϑ between the *xx* and *zz*; **c,f** p-nitrophenol-p-nitrophenol relative orientation as a function of the angle ϑ between the *xx* and *zz*, as a function of the relative distance between the origins. Note the remarkably ordered structure driven by dipole-dipole interactions.

Supplementary Note 5: Additional Structural Information

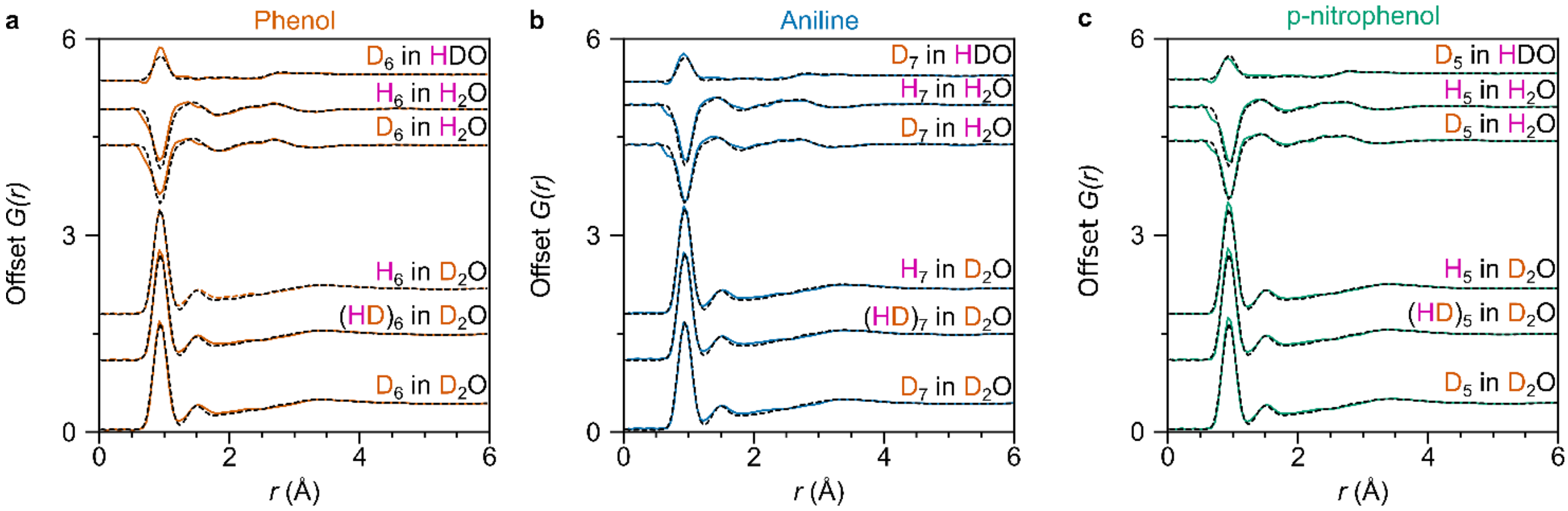

Supplementary Figure 23 Total pair distribution functions *G(r)*. Fourier transformation of the the total structure factors *F(Q)* of Eq. 3 for **a** phenol, **b** aniline, and **c** p-nitrophenol.

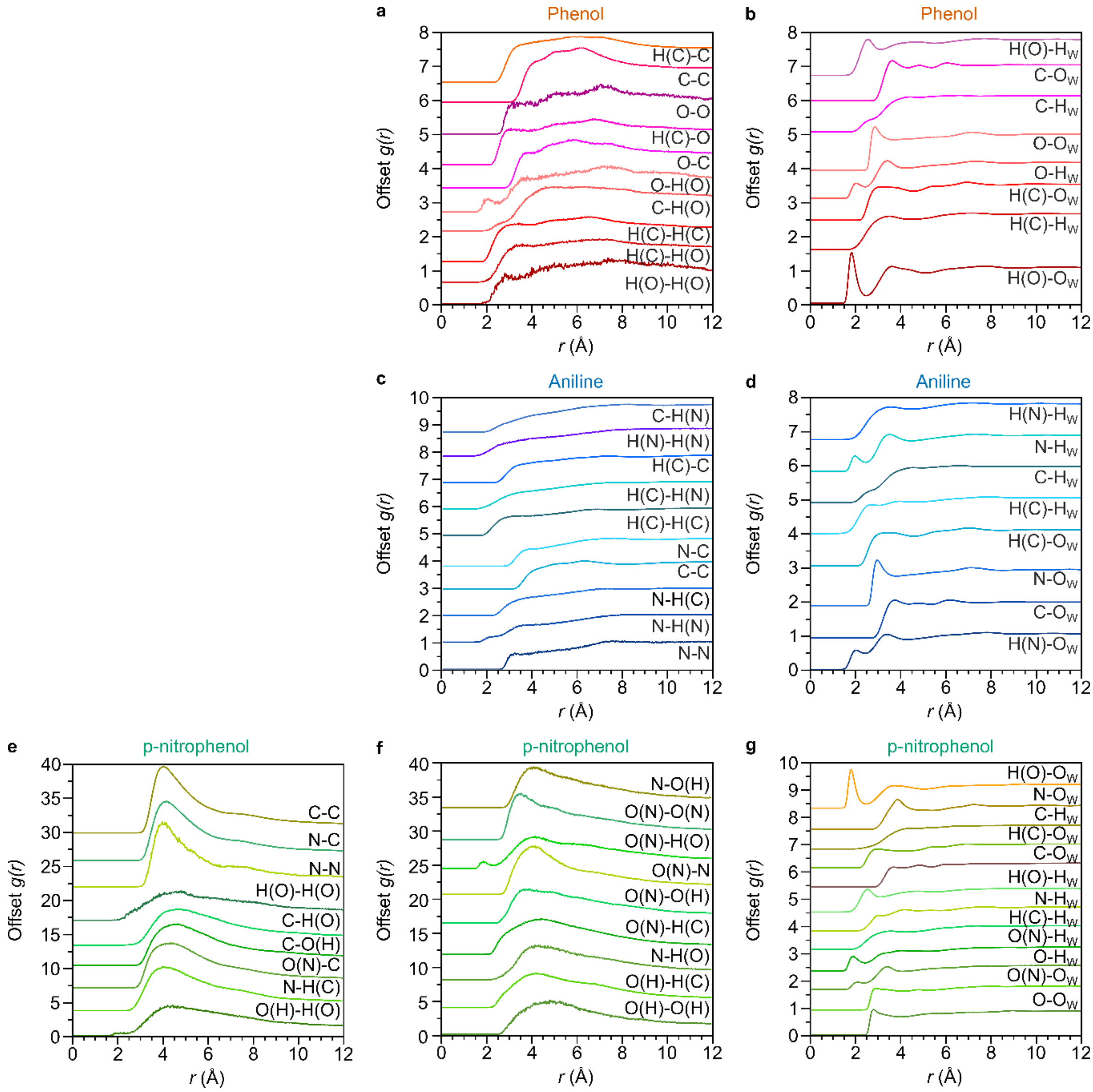

Supplementary Figure 24 Solute-solute and solute-solvent site-site partial distribution functions $g_{\alpha\beta}(r)$. a,b Supplementary phenol-phenol and phenol-water site-site $g_{\alpha\beta}(r)$; **c,d** Aniline-aniline and aniline-water site-site $g_{\alpha\beta}(r)$; **e,f** p-nitrophenol-p-nitrophenol, and **g** p-nitrophenol-water site-site $g_{\alpha\beta}(r)$.

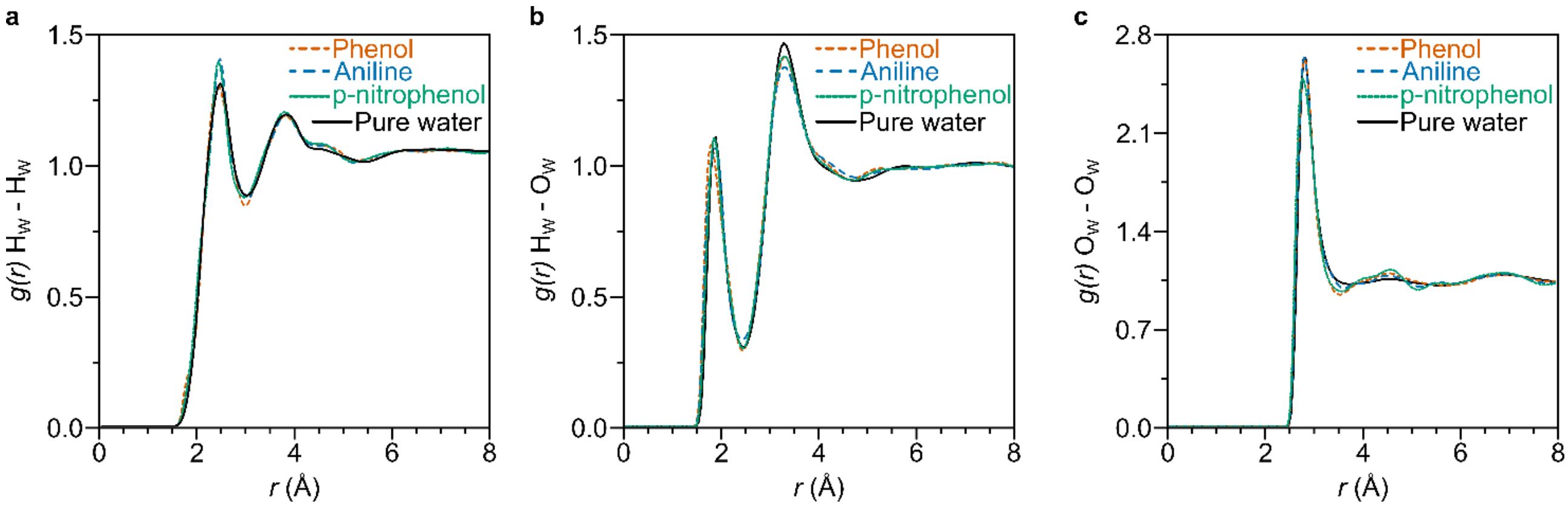

Supplementary Figure 25 Solvent-solvent site-site partial distribution functions *$g_{\alpha\beta}(r)$*. Water-Water Partial Radial Distribution Functions $g_{\alpha\beta}(r)$ relative to **a** H_W-H_W; **b** H_W-O_W and **c** O_W-O_W for phenol–water (orange), aniline–water (blue) and p-nitrophenol-water (green) mixtures.

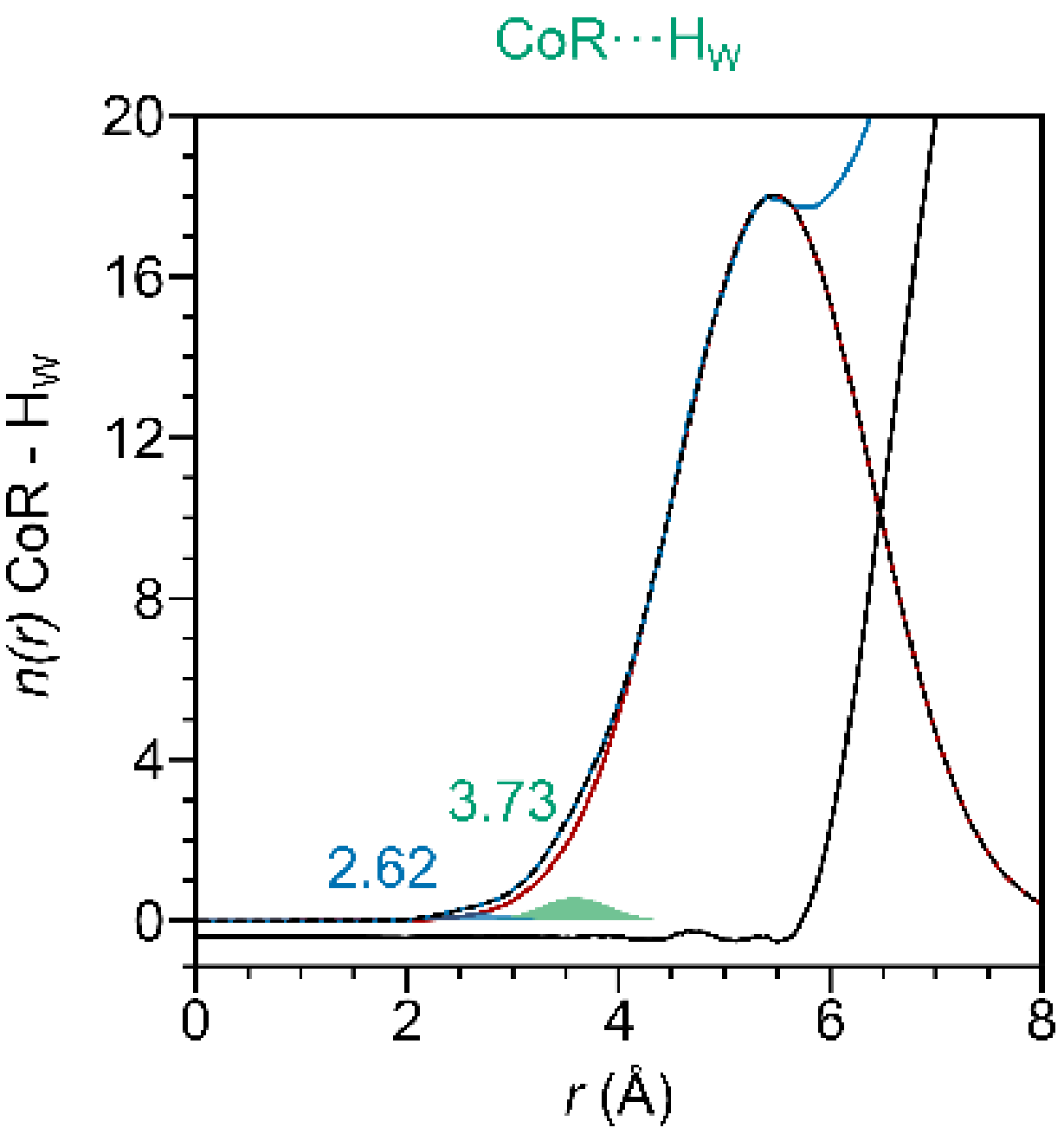

Supplementary Figure 26 OH···π in p-nitrophenol-water. Gaussian fitting of the $n_{CoR\text{-}Hw}(r)$. Note that the first peak is further and broader than the peak determined from the angle distribution of Figure 4h. The peak positions determined from here and Figure 5a do not add to be the O-H distance of 0.95 Å, confirming low directionality.

Supplementary Note 6: Computational Details

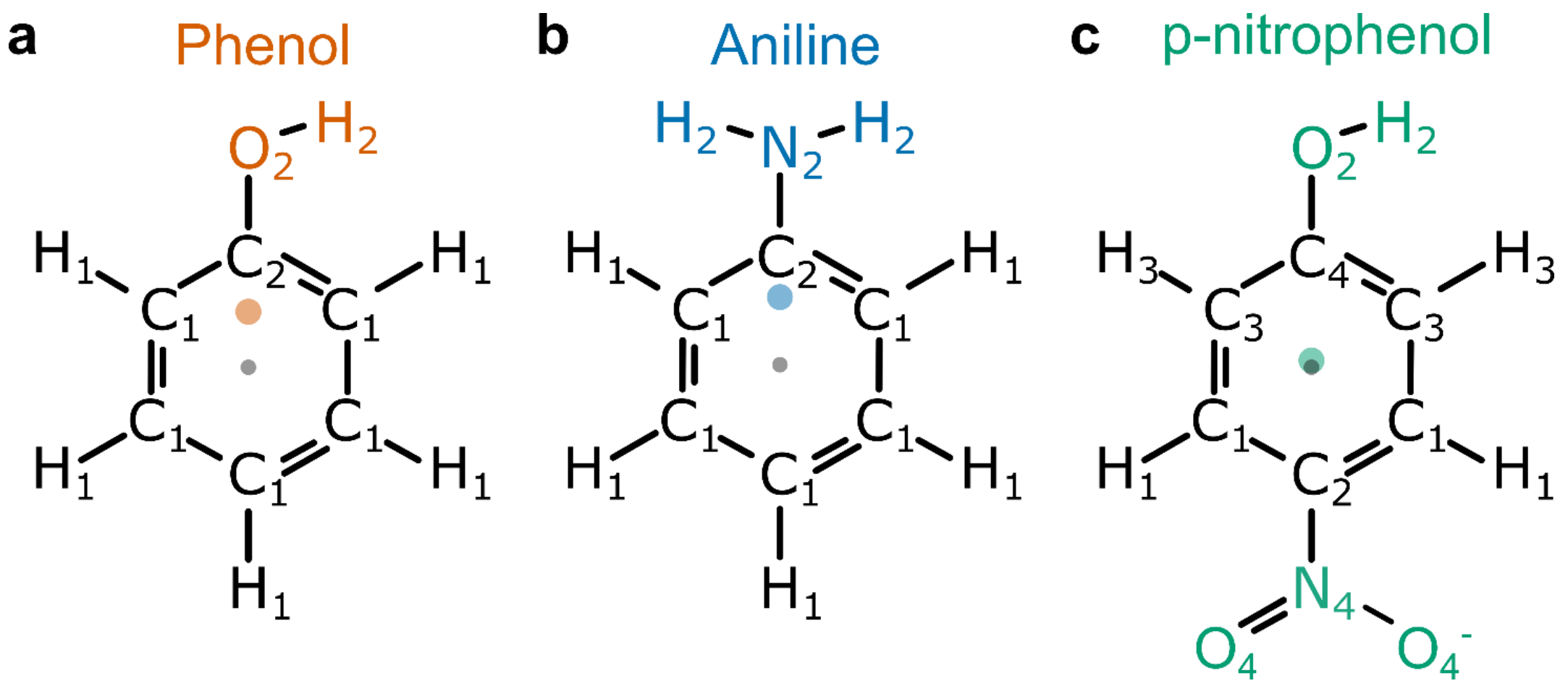

Supplementary Figure 27 Atom labelling. Atomic labelling as per Dissolve refinement related to **a** phenol, **b** aniline, and **c** p-nitrophenol.

Supplementary Table 11 Classical Lennard-Jones parameters and charges. 'Seed' Lennard-Jones parameters and charges from the 1.14*CM1A force field as downloaded from LigParGen for phenol, aniline, and p-nitrophenol. SPC/F_W potential parameters and charges for water.[1-4]

	Atom Type	*σ* (Å)	*ε* (kJ mol^{-1})	*q* (e)
**Phenol**	**C1**	3.55	0.29288	-0.16654
	**C2**	3.55	0.29288	0.134
	**O2**	3.12	0.71128	-0.49883
	**H1**	2.42	0.12552	0.15406
	**H2**	-	-	0.4275
**Aniline**	**C1**	3.55	0.29288	-0.16352
	**C2**	3.55	0.29288	0.20130
	**N2**	3.30	0.71128	-0.9609
	**H1**	2.42	0.12552	0.14724
	**H2**	-	-	0.4205
**p-nitrophenol**	**C1**	3.55	0.29288	-0.0265
	**C2**	3.55	0.29288	0.1881
	**C3**	3.55	0.29288	-0.272
	**C4**	3.55	0.29288	-0.2678
	**O2**	2.96	0.71128	-0.524
	**O4**	3.12	0.71128	-0.4827
	**N2**	3.25	0.50208	0.8368
	**H1**	2.42	0.12552	0.18793
	**H4**	-	-	0.4386
**H_2O**	**H_W**	-	-	0.41
	**O_W**	3.166	0.65030	-0.82

Supplementary Table 12 Molecular geometries. Bond lengths, angles and dihedrals for phenol, aniline, p-nitrophenol, and water. Note torsional parameters and impropers were downloaded from LigParGen and uploaded in dissolve without further modification. *Note that the angle is 113.24° in the SPC/F_W force field. In Dissolve this angle has been reduced to match the experimental *G(r)* of Supplementary Figure 23.

	Bond Length (Å)		Bond Angle (º)		Dihedral Angle (º)	
Phenol	C1 - C1	1.40	C1 – C1 – C1	120.1	H2 – O2 – C2 – C1	0.0
	C1 - C2	1.39	C1 – C2 – C1	120.9	O2 – C2 – C1 – H1	0.0
	C2 - O2	1.36	C1 – C2 – O2	121.4	C2 – C1 – C1 – C1	0.0
	C1 - H1	0.97	C1 – O2 – H2	108.5	C2 – C1 – C1 – H1	-180.0
	H2 - O2	1.09	C2 – C1 – H1	121.3	C1 – C1 – C1 – H1	-180.0
			C1 – C1 – H1	120.0	C1 – C1 – C1 – C1	0.0
Aniline	C1 - C1	1.40	C1 – C1 – C1	120.0	H2 – N2 – C2 – C1	-30.7
	C1 - C2	1.40	C1 – C2 – C1	118.6	N2 – C2 – C1 – H1	6.0
	C2 - N2	1.40	C1 – C2 – N2	120.2	C2 – C1 – C1 – C1	2.1
	H1- C1	1.02	C1 – N2 – H2	113.5	C2 – C1 – C1 – H1	-178.8
	H2 - N2	1.09	C2 – C1 – H1	120.1	C1 – C1 – C1 – C1	0.0
			C1 – C1 – H1	119.9	C1 – C1 – C1 – H1	180.0
			H2 – N2 – H2	112.2		
p-nitrophenol	C1 - C2	1.40	C1 – C2 – C1	121.1	H2 – O2 – C2 – C1	0.0
	C2 - O2	1.36	C1 – C2 – O2	117.5	O2 – C2 – C1 – H1	0.0
	H2 - O2	0.97	C2 – O2 – H2	109.1	C1 – C2 – C1 – H1	180.0
	C1 – H1	1.09	C2 – C1 – H1	121.2	C1 – C2 – C1 – C3	0.0
	C2 – C3	1.39	O4 – N4 – O4	124.5	C3 – C4 – N4 – O4	0.0
	C3 – C4	1.40	C2 – C1 – C3	119.5		
	C4 – N4	1.47	C1 – C3 – H3	119.2		
	O4 – N4	1.24	C1 – C3 – C4	119.6		
	C3 – H3	1.09	C3 – C4 – C3	120.6		
			C3 – C4 – N4	119.7		
			C4 – N4 – O4	117.7		
H_2O	H_w – O_w	0.95	H_w – O_w – H_w	107.13*		

Supplementary Note 7: Torsions and impropers

Torsions and improper torsions are treated using a N-term cosine potential CosN of the form

$$E_{ijkl} = \sum_{n=1}^{N} C_n \left(1 + \cos\left(n\phi_{ijkl}\right)\right) \quad (1)$$

$$F_{ijkl} = -\sum_{n=1}^{N} -nC_n \sin\left(n\phi_{ijkl}\right)\frac{\mathrm{d}\phi}{\mathrm{d}\cos\phi} \quad (2)$$

where *E* is the energy and *F* is the force of the torsions; *i,j,k*, and l are any 4 adjacent sites, *N* is the number of cosine functions; ϕ_{ijkl} is the angle between the *i-j-k* and *j-k-l* planes. Improper torsions are required to enforce planarity of the aromatics, and the functional groups are coplanar with the aromatic free to rotate.

Supplementary Table 13 Proper and improper torsions. Torsions and improper torsions as defined within the OPLS force field from LigParGen. OH, NH_2, and NO_2 groups are set to freely rotate by simply not defining any torsional barrier.

	Torsions			Improper Torsions		
		Form	Parameters		Form	Parameters
Phenol	C-C-O-H	CosN	k_1=0 k_2=-3.5200 k_3=0	C-C-C-O	CosN	k_1=0 k_2=-10.46 k_3=0
	C-C-C-O	CosN	k_1=0 k_2=-15.167 k_3=0	C-C-C-H	CosN	k_1=0 k_2=-10.46 k_3=0
	H-C-C-O	CosN	k_1=0 k_2=-15.167 k_3=0			
	C-C-C-C	CosN	k_1=0 k_2=-15.167 k_3=0			
	C-C-C-H	CosN	k_1=0 k_2=-15.167 k_3=0			
	H-C-C-H	CosN	k_1=0 k_2=-15.167 k_3=0			
Aniline	C-C-N-H	CosN	k_1=0 k_2=-4.2500 k_3=0	N-C-H-H	CosN	k_1=0 k_2=0 k_3=0
	C-C-C-N	CosN	k_1=0 k_2=-15.167 k_3=0	C-C-C-O	CosN	k_1=0 k_2=-10.46 k_3=0
	H-C-C-N	CosN	k_1=0 k_2=-15.167 k_3=0	C-C-C-H	CosN	k_1=0 k_2=-10.46 k_3=0
	C-C-C-C	CosN	k_1=0 k_2=-15.167 k_3=0			
	C-C-C-H	CosN	k_1=0 k_2=-15.167 k_3=0			
	H-C-C-H	CosN	k_1=0 k_2=-15.167 k_3=0			
p-nitrophenol	C-C-O-H	CosN	k_1=0 k_2=-3.5200 k_3=0	N-C-O-O	CosN	k_1=0 k_2=-10.46 k_3=0
	C-C-C-O	CosN	k_1=0 k_2=-15.167 k_3=0	C-C-C-N	CosN	k_1=0 k_2=-10.46 k_3=0
	H-C-C-O	CosN	k_1=0 k_2=-15.167 k_3=0	C-C-C-H	CosN	k_1=0 k_2=-10.46 k_3=0
	C-C-C-C	CosN	k_1=0 k_2=-15.167 k_3=0	C-C-C-O	CosN	k_1=0 k_2=-10.46 k_3=0
	C-C-C-H	CosN	k_1=0 k_2=-15.167 k_3=0			
	H-C-C-H	CosN	k_1=0 k_2=-15.167 k_3=0			
	H-C-C-N	CosN	k_1=0 k_2=-15.167 k_3=0			
	C-C-C-N	CosN	k_1=0 k_2=-15.167 k_3=0			

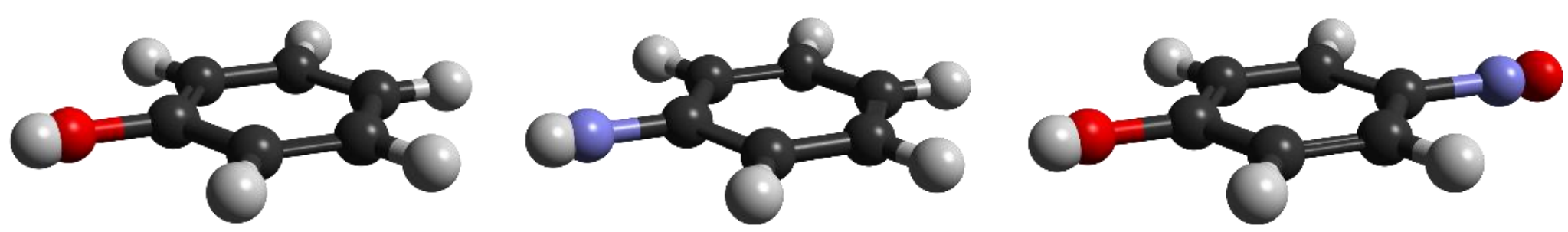

Supplementary Figure 28 Average molecular geometries. Three substituted aromatics average geometries from the SDFs of Figures 2 – 5 before modification by replotting the rotating groups as static. The hydrogen atoms in the OH and NH_2, and the oxygens of NO_2 are collapsed as the result of rotation around the C-O and C-N axes.

Supplementary References

1 Dodda, L. S., Cabeza de Vaca, I., Tirado-Rives, J. & Jorgensen, W. L. LigParGen web server: an automatic OPLS-AA parameter generator for organic ligands. *Nucleic Acids Res.* **45**, W331–W336 (2017). https://doi.org/10.1093/nar/gkx312

2 Jorgensen, W. L., Maxwell, D. S. & Tirado-Rives, J. Development and Testing of the OPLS All-Atom Force Field on Conformational Energetics and Properties of Organic Liquids. *J. Am. Chem. Soc.* **118**, 11225–11236 (1996). https://doi.org/10.1021/ja9621760

3 Jorgensen, W. L. & Tirado-Rives, J. Potential energy functions for atomic-level simulations of water and organic and biomolecular systems. *Proc. Natl. Acad. Sci.* **102**, 6665–6670 (2005). https://doi.org/doi:10.1073/pnas.0408037102

4 Wu, Y., Tepper, H. L. & Voth, G. A. Flexible simple point-charge water model with improved liquid-state properties. *J. Chem. Phys.* **124** (2006). https://doi.org/10.1063/1.2136877
