# Supplementary material for: Weak hydrogen bonding as the driver of aromatic hydration
Source: Nat Commun. 2026 Aug 11;17:9654. doi: 10.1038/s41467-026-76528-x (PMC13558607; doi:10.1038/s41467-026-76528-x)
Supplement: Supplementary file 3 [file 41467_2026_76528_MOESM3_ESM.pdf]

nature portfolio

Peer Review File

Weak hydrogen bonding as the driver of aromatic hydration

Corresponding Author: Dr Camilla Di Mino

This file contains all reviewer reports in order by version, followed by all author rebuttals in order by version.

Version 0:

Reviewer comments:

Reviewer #1

(Remarks to the Author)
In this contribution, the authors studied the solvation of the aromatics phenol, aniline and p-nitrophenol in water. They employed total neutron scattering measurements and fitted the data using modelling-based refinements of a virtual box containing 40.000 molecules with a 1:200 aromatic:water ratio, in line with the concentration of the solutions measures (5 mM).

From their results summarised in Figures 2-4, the authors deduced structural details of the solvation shells around the aromatic molecules in terms of probability distributions of water H and O atoms.

These distributions are consistent with current understanding about non-covalent interactions known from solid state, gas-phase and computational information sources:
1) Typical hydrogen bonding patterns are observed involving the hydroxyl and the amine of the aromatics, as well as the nitro O-atoms.
2) Weaker hydrogen bonding is also observed in patterns involving the aromatics CH`s as donors and the pi-clouds as acceptors.
3) An additional pi-hole type interaction is observed for p-nitrophenol nitro group and water O (Figure 4).

Based on their findings, the authors attempt to rationalize the differences in solubility between phenol (0,895 M), aniline (0,39 M) and p-nitrophenol (0,115 M) by comparing the relative occupancies of the different interaction and note that a marked difference lies in the participation of H-pi type interactions. Indeed, from their data it appears that the H-pi interactions seen for phenol and aniline (H towards the pi clouds) are less pronounced for p-nitrophenol and present a different structure with O towards the pi cloud and the nitro group.

It is very interesting and relevant to learn that such non-covalent forces behave similar in solution as they do the solid state and in the gas phase (and as often computed). Moreover, I agree with the authirs that their technique and findings can shed light on the solubility behaviour or aromatics in water. As such, the paper is a very welcome and important contribution and publication in Nature communications is fully supported.

The authors might want to consider the following comments:
a) The data suggests an overall `coordination number` of about 33 for phenol and aniline, but 29 for p-nitrophenol, despite the latter being larger (Table S1). This distinct difference of `coordination number` requires a more detailed explanation.

When a molecule is fully dissolved, as one might anticipate in a 1:200 ratio system, this implies almost per definition that the molecule is fully surrounded by solvent molecules; whether or not the energetics of each position is favourable. Thus, one really would anticipate that the `coordination number` of water around an aromatic molecule correlates directly with molecular size of the aromatic and that it is more or less a theoretical given (perhaps the authors could list these values as well in the SI?). A deviation from this correlation might indicate the presence of another phenomenon.

The authors rightly considered that there is a proportion of molecules involved in self-association (Table S1 and page 3 of the results and discussion). The `coordination number` of the self-associations from Table S1 appear to be 0,45 for phenol, 0,30 for aniline and 0,73 for p-nitrophenol. It is unclear to me however, how this self-association has been taken into account and what impact this might have on the `coordination number` of water around the aromatic. Might it be the case that a linear

H-bonded dimer diminishes the `coordination number` (only replacing the H-bond) less than a pi-pi stacked dimer (cancelling two OH-pi surfaces)? If so, this might offer a rationale because the proportions between the modes of self-association might well be different, i.e.: similar for phenol and aniline but different for p-nitrophenol.

Phenol and aniline can form hydrogen bonding dimers, co-planar pi-stacked dimers and CH-pi anti-parallel dimers. Such phenomena are likely more pronounced for p-nitrophenol, hence rationalising the higher `coordination number` of the dimer and the lower `coordination number` of water around p-nitrophenol. Indeed, the structure of p-nitrophenol is much more self-complementary from a supramolecular point of view than phenol and aniline; p-nitrophenol can self-associate also by OH-ON hydrogen bonding and the pi-pi stacking geometry is self-complementary (possible concurrent OH-ON and HO-NO interactions in the stacking geometry). See for example some solid state data in:
https://onlinelibrary.wiley.com/iucr/doi/10.1107/S010876810503418X and CSD refcodes NITPOL04-NITPOL09 (available at https://www.ccdc.cam.ac.uk/structures/). If the pi-pi stacking geometry is indeed more pronounced for p-nitrophenol, this might offer a rationale of the lower water `coordination number`.

b) Have the authors considered studying nitrobenzene (solubility about 16 mM and thus feasible)? This would largely eliminate the size-issue and the possible self-association issues (a `coordination number` closer to that found for phenol and aniline would be likely) and will present a good point for comparison purposes of the interaction mode with water. Such a comparison could really complete the picture and strengthen the paper.

c) The barrier of rotation around the C-N bond in nitrobenzene (likely similar in p-nitrophenol) is about 4.1 ± 1.0 kcal/mol (https://link.springer.com/article/10.1007/s11224-007-9186-6). This means that at room temperatures the rotation is relatively free. At the measuring temperature of 70 °C, this rotation will certainly be significant. Could the authors comment on the possible relevance of this rotation for their measurements and data-fitting?

d) To the best of my knowledge, this is the first study of its kind, and it provides very useful and convincing structural data for solution-phase structural characteristics of supramolecular structures. Another technique that is often used to obtain information in the solution phase is NMR: have the authors considered if/how NMR spectroscopy might provide similar information and did they review the literature to check this?

e) The authors speculate that the difference in pi-interactions can be the origin of the difference in solubility, which seems like a very reasonable observation for the lower solubility of p-nitrophenol (0,115 M). Could the authors also speculate about the large difference in solubility between phenol (0,895 M) and aniline (0,39 M)?

f) The graphical representation of the water distributions around the aromatics is strikingly similar to the data presentation method offered by ISOSTAR, which is a tool to assess intermolecular interactions in the solid state (https://www.ccdc.cam.ac.uk/solutions/software/isostar/; https://link.springer.com/article/10.1023/A:1007934413448; https://pubs.acs.org/doi/10.1021/ed200139t;). Interestingly, the distributions around the nitro groups are also consistent with that found in the solid state (https://www.nature.com/articles/ncomms14522; https://chemistry-europe.onlinelibrary.wiley.com/doi/epdf/10.1002/cphc.202000927). Perhaps the authors are interested in comparing their data to such solid-state data.

g) It is stated in the abstract that a subtle interplay of charge distributions determine the outcome of intermolecular processes. While this certainly is a large factor, steric considerations as well as entropy considerations can also play a dominant role (e.g. some ligand-protein processes are even entropically driven). I would suggest the authors adjust this statement in the introduction.

Reviewer #2

(Remarks to the Author)
This manuscript presents a detailed neutron-scattering and EPSR-based study of the hydration of three substituted aromatic molecules, phenol, aniline, and p-nitrophenol. By combining extensive H/D isotopic substitution with total neutron scattering and simulation-based refinement, the authors provide a convincing structural picture of both classical and non-classical solute–water interactions in the liquid state. The experimental approach is demanding and well executed, and the overall agreement between experiment and modelling is good.

A central message of the work is that weak, non-classical interactions between water and the aromatic π system influence the hydration structure, in particular out-of-plane OH···π contacts for phenol and aniline, and interactions consistent with lone pair–π-hole motifs in p-nitrophenol. The comparative approach is a clear strength, as it allows trends to be discussed in relation to the electron-donating or electron-withdrawing nature of the substituents. The finding that p-nitrophenol shows a strongly altered hydration pattern, together with reduced solubility and enhanced solute–solute association, is particularly interesting and well supported by the data.

That said, some aspects of the interpretation would benefit from clarification and slightly more cautious wording. In particular, the term “weak hydrogen bonding” is sometimes used in a way that may overstate the energetic importance of the OH···π interactions. While the structural evidence for these contacts is convincing, it would be helpful to emphasise more clearly their cooperative role within the hydrogen-bonded water network, rather than implying that they act as dominant stabilising interactions on their own. This distinction becomes important when linking local interaction motifs to macroscopic properties such as solubility.

The interpretation of the out-of-plane water–aromatic contacts in p-nitrophenol as lone pair–π-hole interactions is one of the more original aspects of the manuscript and is supported by clear geometrical and orientational features in the EPSR results. However, given the model-dependent nature of EPSR, it would be helpful to state more explicitly that this assignment is based on structural arguments rather than a direct probe of the electron density. A short discussion of alternative or complementary interpretations, such as water structuring driven mainly by strong nitro–water hydrogen bonding, would further strengthen this part of the discussion.

Related to this, the discussion could potentially be strengthened by some complementary input from an electronic structure or electron density perspective. Since much of the interpretation relies on changes in electron distribution, π donation, and the presence of π-holes, even a qualitative theoretical analysis, for example electrostatic potential maps or simple density-based descriptors for the isolated molecules or small solute–water clusters, could help to rationalise the observed trends without the need to model the full liquid environment.

The role of solute–solute interactions in p-nitrophenol also deserves a slightly more explicit discussion. The enhanced antiparallel association driven by the large molecular dipole is clearly demonstrated and likely reduces the accessibility of the aromatic ring for out-of-plane hydration. While this does not contradict the proposed interpretation of disrupted π-related interactions, briefly disentangling solute–solute and solute–solvent effects would make the overall picture clearer.

Finally, the manuscript contains a large amount of quantitative information, including peak positions, coordination numbers, probabilities, and angular distributions, which are carefully analysed but not always easy to compare between the three systems. This is partly due to the way figures and tables are cited, as they are not always introduced in a chronological order (particularly from SI file), making the discussion harder to follow. I also find the extensive use of subfigure labels such as ai, aii, etc., slightly off. It might be worth considering small changes in figure citation and presentation to improve overall readability.
In summary, this is a strong and carefully executed study that provides useful insight into the interplay of classical and non-classical interactions in aromatic hydration. The conclusions are largely supported by the data, and the manuscript should be of interest to a broad readership. I recommend publication after minor to moderate revision, mainly aimed at clarifying the interpretation of weak interactions and improving the overall clarity of the presentation.

Reviewer #3

(Remarks to the Author)
Key results
The authors investigate the hydration of phenol, aniline, and p-nitrophenol in solution using total neutron scattering combined with isotope substitution and structure refinement. Beyond classical hydrogen bonding, the study identifies non-classical interactions, including OH···π contacts, and provides evidence for π-hole lone pair interactions in aqueous solutions. The authors suggest that substituent-induced changes in π electron density modulate water structure and contribute to differences in solubility. The work addresses an important question, namely how subtle electronic effects influence solvation at the molecular level.

Validity
The experimental approach is appropriate and technically sound. The combination of neutron scattering and modeling provides detailed structural insights into interactions involving hydrogen.
However, the interpretation relies heavily on the refinement model, and the manuscript would benefit from a clearer discussion of the following points:
• the uniqueness of the structural solutions,
• the robustness of the assignment of π-hole interactions.
In particular, the identification of π-hole lone pair interactions, while plausible, remains somewhat indirect and should be discussed with more caution. The proposed relationship between microscopic structure and solubility is interesting but not fully proven and may also involve competing effects such as dipole-driven aggregation.

Significance
The study provides valuable experimental evidence for nonclassical interactions between aromatics and water in the liquid state and shows that electronic modulation of the π system influences the structuring of water beyond classical hydrogen bonding. This is particularly relevant for systems in which solvation and aggregation compete with each other, such as π-conjugated arrangements and functional, especially soft, materials.
The manuscript would benefit from a stronger connection to previous work on substituent-dependent electron density distributions in small aromatic systems. In particular, differences or new insights compared to the corresponding solid-state X-ray crystallography and DFT-based and atoms-in-molecules analyses should be discussed.
https://pubs.acs.org/doi/10.1021/jp809601a
https://pubs.acs.org/doi/abs/10.1021/jp105348d
In addition, complementary computational approaches (e.g., CREST/xTB sampling combined with DFT refinement) could be applied or discussed to clarify how efficiently OH···π and π hole interactions are currently captured with standard behavior and considered “good enough.”
https://pubs.acs.org/doi/10.1021/acs.jcim.9b01165
https://crest-lab.github.io/crest-docs/page/examples/qcg/example_5.html

In principle, a brief discussion of relevance beyond biological systems would be valuable, for example, regarding ion or

transport through aromatic molecular layers by means of templated water networks. Together with a corresponding extension of the discussion to interface systems, the general relevance would be strengthened, since water structuring at interfaces is inherently anisotropic, where such electronic effects are likely to be even more pronounced.

Data, methodology and analytical approach
The data are of high quality and the methodology is very appropriate. The use of isotope substitution is an inherent strength. The manuscript would benefit from the following points:
• clearer presentation of uncertainties in important structural parameters,
• more detailed discussion of the sensitivity of the refinement to model assumptions.
The analytical tools (radial distribution functions, coordination numbers, spatial density functions) are appropriate. A more careful distinction between directly observable quantities and model-dependent interpretations is recommended.

Suggested improvements
The manuscript could be improved as follows:
1. Clarification of evidence for π-hole interactions and discussion of alternative interpretations.
2. Discussion of the model dependency and robustness of the refinement results.
3. Specification of uncertainties for important structural variables.
4. Expansion of the discussion on alternative x-ray-based and DFT-based work.
5. Expansion of the discussion on relevance in various applications and material systems.
These points should be able to be addressed briefly within the current scope.

Clarity and context
The manuscript is clear and well structured. The discussion is insightful; a clearer distinction between experimental evidence and conceptual conclusions would be helpful.

References
The literature is cited appropriately for the most part. A somewhat stronger connection to theoretical work and complementary X-ray-based studies (albeit on solids) would be desirable.

Your expertise
My expertise covers intermolecular interactions, solvation phenomena, and supramolecular systems composed of smaller and larger pi-conjugated molecules, particularly at aqueous interfaces. I am less specialized in neutron scattering experiments, but I am confident in assessing their chemical interpretation.
Summary
This is a well-founded and technically sophisticated study that provides rare experimental insights into nonclassical interactions between aromatics and water in solution. While the identification of π-hole effects is fascinating and potentially important, the interpretation would benefit from a more cautious and general formulation.
With moderate revisions, the manuscript could make a valuable contribution to this field of research, particularly in areas where the interaction between electronic structure, solvation, and aggregation determines function.

Reviewer #4

(Remarks to the Author)
I co-reviewed this manuscript with one of the reviewers who provided the listed reports. This is part of the Nature Communications initiative to facilitate training in peer review and to provide appropriate recognition for Early Career Researchers who co-review manuscripts.

Version 1:

Reviewer comments:

Reviewer #1

(Remarks to the Author)
Well done.

Reviewer #2

(Remarks to the Author)
I have carefully read both the revised manuscript and the rebuttal letter and I am pleased to see that the manuscript has been improved substantially.

In particular, I appreciate the more balanced discussion of the role of weak OH···π interactions in aromatic hydration. The revised version now more clearly emphasizes the cooperative nature of these interactions within the broader hydrogen-bonding network of water and avoids overstating their individual energetic contribution to solubility. I also welcome the expanded discussion of solute–solute interactions in p-nitrophenol and their possible influence on hydration and solubility trends.

I further appreciate the clarification regarding the interpretation of the proposed lone pair–π-hole interactions. The authors now explicitly acknowledge the structural nature of the evidence obtained from neutron scattering and EPSR modelling and avoid presenting these interactions as a direct observation of the underlying electron density. In my view, this significantly strengthens the scientific rigor of the discussion.

The additional analyses included in the revised manuscript and Supporting Information, particularly those related to coordination numbers, self-association, and hydration structure, help to clarify several points that were previously insufficiently discussed. The manuscript is also easier to follow, and the overall presentation has improved.

While some aspects of the interpretation inevitably remain model-dependent, as is common for this type of study, I believe that the authors have addressed my concerns satisfactorily and that the conclusions are now presented in an appropriately balanced and cautious manner.

I therefore support publication of the manuscript in its present form.

Reviewer #3

(Remarks to the Author)
The authors have addressed all comments thoroughly and specifically. I recommend the manuscript for publication without further changes.

Open Access This Peer Review File is licensed under a Creative Commons Attribution 4.0 International License, which permits use, sharing, adaptation, distribution and reproduction in any medium or format, as long as you give appropriate credit to the original author(s) and the source, provide a link to the Creative Commons license, and indicate if changes were made.
In cases where reviewers are anonymous, credit should be given to 'Anonymous Referee' and the source.
The images or other third party material in this Peer Review File are included in the article's Creative Commons license, unless indicated otherwise in a credit line to the material. If material is not included in the article's Creative Commons license and your intended use is not permitted by statutory regulation or exceeds the permitted use, you will need to obtain permission directly from the copyright holder.
To view a copy of this license, visit https://creativecommons.org/licenses/by/4.0/

Reviewer #1 (Remarks to the Author):

In this contribution, the authors studied the solvation of the aromatics phenol, aniline and p-nitrophenol in water. They employed total neutron scattering measurements and fitted the data using modelling-based refinements of a virtual box containing 40.000 molecules with a 1:200 aromatic:water ratio, in line with the concentration of the solutions measures (5 mM).

Authors Response:

- *We thank the Reviewer for the clear summary of our work. We note that the concentration of the solutions is 275mM and not 5mM, as the molarity of pure water is 55 M and not 1M. We realise that the way we presented the concentrations in the original manuscript lacked clarity, we therefore amended the method section to include the concentration in molarity for consistency with the saturation values presented in the introduction. We also note that 275mM is higher than the saturation limit for p-nitrophenol at room temperature, but at 70° the solubility increases to 540mM (~1:100 molecular ratio) (N. V. Sidgwick et al., J. Chem. Soc. Trans. 1915, 107, 1202).*

Changes to the Manuscript:

- Page 9 Line 19: “(~0.275 M)”
- Added References
    - [33] N. V. Sidgwick, P. Pickford, and B. H. Wilsdon, *J. Chem. Soc., Trans.* **1911**, 99, 1122.
    - [34] A. E. Hill and W. M. Malisoff, *J. Am. Chem. Soc.* **1926**, 48, 4, 918.
    - [36] N. V. Sidgwick, W. J. Spurrell, and T. E. Davies, *J. Chem. Soc. Trans.* **1915**, 107, 1202.

From their results summarised in Figures 2-4, the authors deduced structural details of the solvation shells around the aromatic molecules in terms of probability distributions of water H and O atoms.

These distributions are consistent with current understanding about non-covalent interactions known from solid state, gas-phase and computational information sources:
1) Typical hydrogen bonding patterns are observed involving the hydroxyl and the amine of the aromatics, as well as the nitro O-atoms.
2) Weaker hydrogen bonding is also observed in patterns involving the aromatics CH`s as donors and the pi-clouds as acceptors.
3) An additional pi-hole type interaction is observed for p-nitrophenol nitro group and water O (Figure 4).

Based on their findings, the authors attempt to rationalize the differences in solubility between phenol (0,895 M), aniline (0,39 M) and p-nitrophenol (0,115 M) by comparing the relative occupancies of the different interaction and note that a marked difference lies in the participation of H-pi type interactions. Indeed, from their data it appears that the H-pi interactions seen for phenol and aniline (H towards the pi clouds) are less pronounced for p-nitrophenol and present a different structure with O towards the pi cloud and the nitro group.

It is very interesting and relevant to learn that such non-covalent forces behave similar in solution as they do the solid state and in the gas phase (and as often computed). Moreover, I agree with the authors that their technique and findings can shed light on the solubility behaviour or aromatics in water. As such, the paper is a very welcome and important contribution and publication in Nature communications is fully supported.

Authors Response:

- *We thank the Reviewer for their clear summary, appreciation of our work, and recommendation for publication.*

The authors might want to consider the following comments:
a) The data suggests an overall `coordination number` of about 33 for phenol and aniline, but 29 for p-nitrophenol, despite the latter being larger (Table S1). This distinct difference of `coordination number` requires a more detailed explanation.

Authors Response:

- *We agree that discussing all the possible scenarios for the decrease in coordination number is important. We therefore modified the text accordingly to include 1) the lack of cooperation as suggested later by the Reviewer, 2) the less coordinated water structuring arising from the "linear" hydrogen bonded dimers of water above and below the ring, and 3) a higher degree of self-association driven by dipole-dipole interactions that concurrently decreases solubility. We have computed the coordination number for the solute-solute hydrogen bonds, and we believe they are too low and too short range to be considered significant for self-association (0.002 for OH···O(H) hydrogen bonding and 0.006 for OH···O(N)), however we have included them in the Supplementary Information.*

Changes to Manuscript:

- *Main Manuscript Page 7 Line 15: "These non-conventional contacts have a clear effect on the overall hydration of p-nitrophenol, decreasing the overall water coordination from the theoretical value (from 36 to 29, Supplementary Table 5). The decrease can be ascribed to the concurrent lack of cooperation that does not facilitate water close approaches, with the consequent looser water structuring arising from the "linear" water dimers above and below the ring. In addition, there is a higher degree of self-association driven by dipole-dipole interactions that contribute to decrease solubility."*

When a molecule is fully dissolved, as one might anticipate in a 1:200 ratio system, this implies almost per definition that the molecule is fully surrounded by solvent molecules; whether or not the energetics of each position is favourable. Thus, one really would anticipate that the `coordination number` of water around an aromatic molecule correlates directly with molecular size of the aromatic and that it is more or less a theoretical given (perhaps the authors could list these values as well in the SI?). A deviation from this correlation might indicate the presence of another phenomenon.

Authors Response:

- *The decrease of the number of water molecules around the aromatics is of particular significance as the minima of the aromatic-water centre-of-geometry partial radial distribution functions are at the same distance (Figure 1cii). To follow the Reviewer suggestion, we can consider the aromatic molecules like disks contained in a sphere of radius 2.82 Å, 2.91 Å, and 3.27 Å for phenol, aniline, and p-nitrophenol respectively. The radius was determined by halving the distance between the two furthest points within the molecules. Through close packing of spheres, we can fit 23.4, 25.8, and 36.6 water molecules respectively, which is interesting as the trend we measure is the opposite of the prediction from these theoretical values, indicating a disrupted water network. We have added these values to Supplementary Table 5 in the Supplementary Information file.*

Changes to Manuscript:

- *Supplementary Information Page 11, Supplementary Table 5 Caption: "Theoretical coordination numbers were evaluated considering the number of water molecules (spheres of radius 0.95 Å) within a sphere of radius 2.82 Å, 2.91 Å, and 3.27 Å for phenol, aniline, and p-nitrophenol. Radii were evaluated as half of the distance between the two furthest atoms in the plane of the aromatic in the Avogadro software.[1] Note the theoretical values increase for p-nitrophenol that is the opposite trend we observe from our analysis."*
- *Supplementary Table 5: Added Column 5 rows 4 – 6: 23.4, 25.8, 36.6*
- *Added Supplementary Reference:*
    - [1] M. D. Hanwell *et al*., *J. Cheminform.* **2012**, 4, 17.

The authors rightly considered that there is a proportion of molecules involved in self-association (Table S1 and page 3 of the results and discussion). The `coordination number` of the self-associations from Table S1 appear to be 0,45 for phenol, 0,30 for aniline and 0,73 for p-nitrophenol. It is unclear to me however, how this self-association has been taken into account and what impact this might have on the `coordination number` of water around the aromatic. Might it be the case that a linear H-bonded dimer diminishes the `coordination number` (only replacing the H-bond) less than a pi-pi stacked dimer (cancelling two OH-pi surfaces)? If so, this might offer a rationale because the proportions between the modes of self-association might well be different, i.e.: similar for phenol and aniline but different for p-nitrophenol.

Authors Response:

- *We thank the Reviewer for the comment, and we have now clarified this point in the manuscript. Self-association is implicitly considered in the solute-solute coordination numbers. These are defined as the number of molecules present in a sphere of radius extracted from the first minimum of the Centre-of-Geometry (CoG)-CoG g(r). In our case this minimum is at 6.5 Å from the centre of the aromatic geometry, averaged over all the configurations. We note that our calculation includes self-association but also solvent mediated pairs within the cut-off distance. In both cases, the approach is very directional, where two p-*

nitrophenol molecules approach preferentially in an antiparallel fashion (Supplementary Figure 22).

Changes to Manuscript:

- *Main Manuscript Page 4 Line 8: "These solute-solute coordination numbers implicitly contain information about the average self-association of these substituted aromatics in water."*

Phenol and aniline can form hydrogen bonding dimers, co-planar pi-stacked dimers and CH-pi anti-parallel dimers. Such phenomena are likely more pronounced for p-nitrophenol, hence rationalising the higher `coordination number` of the dimer and the lower `coordination number` of water around p-nitrophenol. Indeed, the structure of p-nitrophenol is much more self-complementary from a supramolecular point of view than phenol and aniline; p-nitrophenol can self-associate also by OH-ON hydrogen bonding and the pi-pi stacking geometry is self-complementary (possible concurrent OH-ON and HO-NO interactions in the stacking geometry). See for example some solid state data in: https://onlinelibrary.wiley.com/iucr/doi/10.1107/S010876810503418X and CSD refcodes NITPOL04-NITPOL09 (available at https://www.ccdc.cam.ac.uk/structures/). If the pi-pi stacking geometry is indeed more pronounced for p-nitrophenol, this might offer a rationale of the lower water `coordination number`.

Authors Response:

- *The coordination number for OH···O(H) and OH···O(N) hydrogen bonds is 0.01 (Supplementary Table 6). We believe the solution association is driven almost entirely by dipole-dipole interaction. Considering how low these coordination numbers are, and that a shoulder is present in the CoG-CoG p-nitrophenol g(r) (Figure 1ci) at ~5Å, we can deduce that p-nitrophenol contacts are in part water mediated. Interestingly, in solution p-nitrophenol out-of-plane contacts are antiparallel (Supplementary Figure 22), while in the solid state are parallel [42]. We have added this note to the main text.*

Changes to Manuscript:

- *Supplementary Information Page 11. Added Supplementary Table 6: "Site-site coordination numbers for hydroxyl, amine, and nitro groups in solute-solute contacts. Note that the coordination number depends on the number of sites, $n_{\alpha\beta} \neq n_{\beta\alpha}$"*
- *Main Manuscript Page 4 line 2: "Solute – solute hydrogen bonding is not thought to have a strong effect on these contacts, given the almost negligible coordination numbers (Supplementary Table 6)."*
- *Added Reference:*
    - [45] G. Wójcik and I. Mossakowska, *Acta Cryst.* **2006**, B62, 143.

b) Have the authors considered studying nitrobenzene (solubility about 16 mM and thus feasible)? This would largely eliminate the size-issue and the possible self-association issues (a `coordination number` closer to that found for phenol and aniline would be likely)

and will present a good point for comparison purposes of the interaction mode with water. Such a comparison could really complete the picture and strengthen the paper.

Authors Response:

- *We agree with the Reviewer that nitrobenzene would be an ideal comparison. However, we need to note that the concentration of the solutions of this study is 0.275M. Nitrobenzene is an order of magnitude less soluble than p-nitrophenol (1:3600 at 20 °C), therefore we would be in a dilution regime below the resolution of the experimental technique. Even at 60 °C, nitrobenzene would reach 25mM (1:2200) [R1,R2] which is still unfortunately too low being over an order of magnitude more diluted than our current work. New instrumentation available to users at the end of 2027 will allow us to work on more diluted systems.*

  [R1] H. S. Davis, "Supersaturated Solutions of Liquids in Liquids." *J. Am. Chem. Soc.* **1916**, 1166.

  [R2] H. E. Vermillion, "Solubility Studies. VI. The Solubility of Nitrobenzene in Deuterium Water and in Ordinary Water" *J. Am. Chem. Soc.* **1941**, 63, 1346.

Changes to Manuscript:

- *Main Manuscript Page 7 Line 47: "While understanding the effects of electron withdrawing groups in isolation is highly desirable, such as in the case of nitrobenzene, p-nitrophenol is at the edge of solubility to make these measurements accessible. A related study would be possible for example by growing amorphous ice around the hydrophobic species. [54]"*
- *Added Reference:*
  - [54] S. K. Talewar, *et al., Faraday Discuss.* **2024**, 249, 69.

c) The barrier of rotation around the C-N bond in nitrobenzene (likely similar in p-nitrophenol) is about 4.1 ± 1.0 kcal/mol (https://link.springer.com/article/10.1007/s11224-007-9186-6). This means that at room temperatures the rotation is relatively free. At the measuring temperature of 70 °C, this rotation will certainly be significant. Could the authors comment on the possible relevance of this rotation for their measurements and data-fitting?

Authors Response:

- *We thank the Reviewer for the comment. OH, NH_2 and NO_2 groups are set to freely rotate around the C-O and C-N axes via the definition of torsional parameters. When the torsional energy is low, they freely rotate around the torsional axis. Another way to allow the group to rotate is to leave the torsion unconstrained. We have added a supplementary note where we list the torsional parameters in Supplementary Table 12. We have also added a note in the Method Section for clarity.*

Changes to Manuscript:

- *Main Manuscript Page 10 line 19: "The OH groups in phenol and p-nitrophenol, as well as the nitro group NO_2 and the amino NH_2 in p-nitrophenol and aniline are*

allowed to freely rotate along the C-O and the C-N axes. Torsional parameters are listed in Supplementary Table 12."

- *Added "Supplementary Note 5: Torsions and Impropers"*
- *Supplementary Information Page 21: Added Supplementary Table 13*
- *Added Supplementary Figure 28: "Average molecular geometries of the three substituted aromatics of the SDFs of Figures 2 – 5 before replotting the rotating groups. The hydrogen atoms in the OH and NH_2, and the oxygens of NO_2 are collapsed as the result of rotation around the C-O and C-N axes."*

d) To the best of my knowledge, this is the first study of its kind, and it provides very useful and convincing structural data for solution-phase structural characteristics of supramolecular structures. Another technique that is often used to obtain information in the solution phase is NMR: have the authors considered if/how NMR spectroscopy might provide similar information and did they review the literature to check this?

Authors Response:

- *We agree with the Reviewer that solution-phase NMR is a widely used and valuable tool. However, in our case, its applicability is limited. We would primarily be restricted to monitoring the proton shift of water, as the centre of the aromatic ring does not provide a specific probeable site. For the aromatic system ^{13}C NMR would not be able to distinguish between the influence on water molecules surrounding the ring and the structuring above and below it, particularly given the limited concentration range accessible due to poor solubility that hinders the possibility of investigating a concentration series.*

  Additionally, the NMR spectra would be strongly influenced by the substituents, which differ significantly in their chemical nature, electron-donating/withdrawing properties, and geometry. Furthermore, the rapid proton exchange between water and OH/NH_2 would lead to substantial peak broadening, especially at elevated temperatures (e.g., 70 °C). As the effects under investigation are subtle, such broadening would likely obscure any measurable shifts within the instrument's resolution.

  This challenge is complicated by the absence of a suitable absolute reference standard for comparison. To address this, we have added a paragraph in the Introduction discussing complementary NMR approaches.

Changes to Manuscript:

- *Main Manuscript Page 2 Line 35: "Nuclear magnetic resonance is a powerful tool to identify and quantify the effects of non-covalent interactions both in the solid and in the liquid state. These include intra and intermolecular classical hydrogen bonding, X-H···π interactions, and non-conventional lone pair-π*. [27-30]"*
- *Added references:*
  - [27] F. E. Hanna *et al., Chem. Sci*. **2025**, 16, 7894.
  - [28] L. Trevisan *et al.*, *Chem. Sci.* **2025**, 16, 7418.
  - [29] L. Trevisan, *et al.*, *J. Am. Chem. Soc.* **2022**, 144, 42, 19499.
  - [30] L. Russo, *et al., Nat. Commun.* **2025**, 16, 10231.

e) The authors speculate that the difference in pi-interactions can be the origin of the difference in solubility, which seems like a very reasonable observation for the lower solubility of p-nitrophenol (0,115 M). Could the authors also speculate about the large difference in solubility between phenol (0,895 M) and aniline (0,39 M)?

Authors Response:

- *We believe that the difference in solubility can be ascribed to the lower electron donating power of the NH_2 to the aromatic due to the partial (and not total) delocalisation of the N lone pair over the ring given the tilted geometry, and to the greater disruption of the water network around the NH_2 group vs the OH. The OH group lies in the plane of the ring while the NH_2 group in aniline is tetrahedral with a low flipping barrier that hinders a water close approach (as seen from the lowered hydrogen bonding ability).*

Changes to the Manuscript

- *Main Manuscript Page 6 Line 17: "A similar argument can be made for aniline, whose solubility is half the one of phenol. The partial delocalisation of the nitrogen lone pair over the ring fails to enforce the planarity of the NH_2 group (and vice versa) while reducing the ring's electron richness compared to phenol. Consequently, the water structure around aniline will be further disrupted than in the case of phenol by the low flipping barrier that hinders the close approach of a water molecule, and concurrently reduces the number of hydrogen bonds (Supplementary Table 6)."*

f) The graphical representation of the water distributions around the aromatics is strikingly similar to the data presentation method offered by ISOSTAR, which is a tool to assess intermolecular interactions in the solid state (https://www.ccdc.cam.ac.uk/solutions/software/isostar/; https://link.springer.com/article/10.1023/A:1007934413448; https://pubs.acs.org/doi/10.1021/ed200139t;). Interestingly, the distributions around the nitro groups are also consistent with that found in the solid state (https://www.nature.com/articles/ncomms14522; https://chemistry-europe.onlinelibrary.wiley.com/doi/epdf/10.1002/cphc.202000927). Perhaps the authors are interested in comparing their data to such solid-state data.

Authors Response:

- *We agree that comparing to solid-state data is highly relevant. We have therefore added a paragraph in the* Results and Discussion *where we note the similarities.*

Changes to Manuscript:

- *Main Manuscript Page 5 Line 15: "It is interesting to note that the water structuring around the nitro group in solution is similar to what previously observed in the solid state.[50, 51]"*
- *Added References:*
  - [50] A. Báuza *et al.*, *Nat. Commun.* **2017**, 8, 14522.
  - [51] T. J. Mooibroek *et al.*, *ChemPhysChem* **2021**, 22, 141.

g) It is stated in the abstract that a subtle interplay of charge distributions determine the outcome of intermolecular processes. While this certainly is a large factor, steric considerations as well as entropy considerations can also play a dominant role (e.g. some ligand-protein processes are even entropically driven). I would suggest the authors adjust this statement in the introduction.

Authors Response:

- *We agree with the reviewer that intermolecular processes are more than just charge distributions. We have therefore amended the introduction to include configurational entropies and sterics.*

Changes to Manuscript

- *Main Manuscript Page 1 Line 19: "From liquid miscibility to the formation of organic crystals and biological structures, the subtle interplays of charge distribution, entropy, and sterics determine the outcome."*

Reviewer #2 (Remarks to the Author):

This manuscript presents a detailed neutron-scattering and EPSR-based study of the hydration of three substituted aromatic molecules, phenol, aniline, and p-nitrophenol. By combining extensive H/D isotopic substitution with total neutron scattering and simulation-based refinement, the authors provide a convincing structural picture of both classical and non-classical solute–water interactions in the liquid state. The experimental approach is demanding and well executed, and the overall agreement between experiment and modelling is good.

A central message of the work is that weak, non-classical interactions between water and the aromatic π system influence the hydration structure, in particular out-of-plane OH···π contacts for phenol and aniline, and interactions consistent with lone pair–π-hole motifs in p-nitrophenol. The comparative approach is a clear strength, as it allows trends to be discussed in relation to the electron-donating or electron-withdrawing nature of the substituents. The finding that p-nitrophenol shows a strongly altered hydration pattern, together with reduced solubility and enhanced solute–solute association, is particularly interesting and well supported by the data.

Authors Response:

- *We thank the reviewer for the clear summary and appreciation of our work.*

That said, some aspects of the interpretation would benefit from clarification and slightly more cautious wording. In particular, the term "weak hydrogen bonding" is sometimes used in a way that may overstate the energetic importance of the OH···π interactions. While the structural evidence for these contacts is convincing, it would be helpful to emphasise more clearly their cooperative role within the hydrogen-bonded water network, rather than implying that they act as dominant stabilising interactions on their own. This distinction becomes important when linking local interaction motifs to macroscopic properties such as solubility.

Authors Response:

- *We have included the computed energy of interaction in the introduction to further emphasise that the OH···π interaction is weak and that any strong structuring arises from cooperation. We believe it is an important point to highlight to further understand the difference in solubility between phenol and aniline, as raised by Reviewer 1.*

Changes to Manuscript:

- *Main Manuscript Page 3 Line 12:* ***"****…the estimated OH···π interaction is weak (10 and 2.5 kJ/mol) [41, 42]****"***
- *Added References:*
    - [41] H. H. Y. Tsui *et al,. Chem. Phys. Lett.* **2001**, 350, 5, 565.
    - [42] G. Piani *et al., Chem. Phys.* **2006**, 330, 1, 138.

The interpretation of the out-of-plane water–aromatic contacts in p-nitrophenol as lone pair–π-hole interactions is one of the more original aspects of the manuscript and is supported by

clear geometrical and orientational features in the EPSR results. However, given the model-dependent nature of EPSR, it would be helpful to state more explicitly that this assignment is based on structural arguments rather than a direct probe of the electron density. A short discussion of alternative or complementary interpretations, such as water structuring driven mainly by strong nitro–water hydrogen bonding, would further strengthen this part of the discussion.

Authors Response:

- *We are glad that the referee supports our data analysis and interpretation. We agree with the Reviewer that our interpretation of the lone pair–π-hole comes exclusively from structural data. While our characterisation approach is only sensitive to the atomic positions, these positions are related to the local electronic structure. We do note that our findings are corroborated by previous DFT studies which show the LUMO density aligns with the offset position of the water-oxygen density. The possibility of the water-aromatic structure being driven by nitro-water interactions was present in the original manuscript, but we have expanded the discussion to further clarify this point.*

Changes to Manuscript:

- *Main Manuscript Page 6 Lines 39: "Although this motif might arise simply from the structuring of the hydrogen bonded network of water around the nitro group, the distance and the directionality support the notion of an actual close contact between the non-bonding electrons of the oxygen and the region of positive charge (π-hole) that is present in the electron density distribution of this highly polarised molecule. In fact, the oxygen SDF sits slightly off-set towards the OH group, with the oxygen lone-pair in the direction of the LUMO.[16] Although we do not probe electron densities, our analysis is sensitive to atom positions that are related to electron distributions, therefore we can take our data as structural evidence for π-hole - Ow bonding in the liquid state, where the geometry of such an interaction is best described by the oxygen lone-pair pointing towards the aromatic ring"*

Related to this, the discussion could potentially be strengthened by some complementary input from an electronic structure or electron density perspective. Since much of the interpretation relies on changes in electron distribution, π donation, and the presence of π-holes, even a qualitative theoretical analysis, for example electrostatic potential maps or simple density-based descriptors for the isolated molecules or small solute–water clusters, could help to rationalise the observed trends without the need to model the full liquid environment.

Authors Response:

- *We agree that a clearer explanation of the electronic distribution would help contextualise the structures we see. We have now expanded the discussion to include the IsoStar database as suggested by Reviewer 2.*

Changes to Manuscript:

- *Main Manuscript Page 5 Line 15: "It is interesting to note that the water structuring around the nitro group in solution is similar to that previously observed in the solid state."*
- *Added References:*
    - [50] A. Báuza *et al.*, *Nat. Commun.* **2017**, 8, 14522.
    - [51] T. J. Mooibroek *et al.*, *ChemPhysChem* **2021**, 22, 141.

The role of solute–solute interactions in p-nitrophenol also deserves a slightly more explicit discussion. The enhanced antiparallel association driven by the large molecular dipole is clearly demonstrated and likely reduces the accessibility of the aromatic ring for out-of-plane hydration. While this does not contradict the proposed interpretation of disrupted π-related interactions, briefly disentangling solute–solute and solute–solvent effects would make the overall picture clearer.

Authors Response:

- *We agree that solute-solute interactions are important. We have therefore expanded the discussion, including the hindering of one face of the aromatic that impacts the average coordination number, linear-waters that do not approach the ring as closely due to lack of cooperative OH···π, and molecular recognition between 2 p-nitrophenol molecules that is remarkable.*

Changes to Manuscript:

- *Main Manuscript Page 7 Line 15: "These non-conventional contacts have a clear effect on the overall hydration of p-nitrophenol, decreasing the overall water coordination from the theoretical value (from 36 to 29, Supplementary Table 5). The decrease can be ascribed to the concurrent lack of cooperation that does not facilitate water close approaches, with the consequent looser water structuring arising from the "linear" water dimers above and below the ring. In addition, there is a higher degree of self-association driven by dipole-dipole interactions that contribute to decrease solubility."*

Finally, the manuscript contains a large amount of quantitative information, including peak positions, coordination numbers, probabilities, and angular distributions, which are carefully analysed but not always easy to compare between the three systems. This is partly due to the way figures and tables are cited, as they are not always introduced in a chronological order (particularly from SI file), making the discussion harder to follow. I also find the extensive use of subfigure labels such as ai, aii, etc., slightly off. It might be worth considering small changes in figure citation and presentation to improve overall readability.

Authors Response:

- *We agree with the Reviewer that not citing Figures in chronological order can be hard to follow. We have therefore restructured the Supplementary Information File. We have also split Figure 4 Hydration of the NO_2 group and analysis of the O···π* contacts into a) Figure 4 Analysis of the O···π* contacts and b) Figure 5 Hydration*

of the NO_2 group. We have also relabelled the subfigures so that we refer with the same letter to sub-figures that show the same structural information within the same panel (e.g., ai, aii, … partial distribution functions; d spatial density functions) to further ease the readability.

Changes to Manuscript:

- *Original Manuscript Figure 4 is now Main Manuscript Figure 3 Hydration of the NO_2 group and Figure 5 Analysis of the O⋯π* contacts.*
- *Subfigures of Figure 2, 3, 4, 5:*
    - Original Manuscript Figure 2bi is now Manuscript Figure 2aii
    - Original Manuscript Figure 2ci is now Manuscript Figure 2aiii
    - Original Manuscript Figure 2aii is now Manuscript Figure 2bi
    - Original Manuscript Figure 2aiii is now Manuscript Figure 2ci
    - Original Manuscript Figure 2aiv is now Manuscript Figure 2di
    - Original Manuscript Figure 2biii is now Manuscript Figure 2cii
    - Original Manuscript Figure 2biv is now Manuscript Figure 2dii
    - Original Manuscript Figure 2cii is now Manuscript Figure 2biii
    - Original Manuscript Figure 2civ is now Manuscript Figure diii
    - Original Manuscript Figure 3aii is now Manuscript Figure 4bi
    - Original Manuscript Figure 3aiii is now Manuscript Figure 4ci
    - Original Manuscript Figure 3bi is now Manuscript Figure 4aii
    - Original Manuscript Figure 3biii is now Manuscript Figure 4cii
    - Original Manuscript Figure 3ci is now Manuscript Figure 4aiii
    - Original Manuscript Figure 3cii is now Manuscript Figure 4biii
    - Original Manuscript Figure 4aiii is now Manuscript Figure 3b
    - Original Manuscript Figure 4bi is now Manuscript Figure 3c
    - Original Manuscript Figure 4bii is now Manuscript Figure 3d
    - Original Manuscript Figure 4ci is now Manuscript Figure 5a
    - Original Manuscript Figure 4cii is now Manuscript Figure 5bi
    - Original Manuscript Figure 4ciii is now Manuscript Figure 5bii

Supplementary Information File:

- Supplementary Note 1 is now Supplementary Note 3
- Supplementary Note 2 is now Supplementary Note 5
- Supplementary Note 3 is now Supplementary Note 4
- Supplementary Note 4 is now Supplementary Note 6
- Supplementary Note 5 is now Supplementary Note 1
- Supplementary Table 1 is now Supplementary Table 5
- Supplementary Table 2 is now Supplementary Table 7
- Supplementary Table 3 is now Supplementary Table 8
- Supplementary Table 4 is now Supplementary Table 9
- Supplementary Table 5 is now Supplementary Table 10
- Supplementary Table 6 is now Supplementary Table 11
- Supplementary Table 7 is now Supplementary Table 12
- Supplementary Table 8 is now Supplementary Table 1
- Supplementary Table 9 is now Supplementary Table 2
- Supplementary Table 10 is now Supplementary Table 3

- Supplementary Table 11 is now Supplementary Table 4
- Supplementary Figure 1 is now Supplementary Figure 23
- Supplementary Figure 2 is now Supplementary Figure 24
- Supplementary Figure 3 is now Supplementary Figure 25
- Supplementary Figure 4 is now Supplementary Figure 26
- Supplementary Figure 5 is now Supplementary Figure 19
- Supplementary Figure 6 is now Supplementary Figure 20
- Supplementary Figure 7 is now Supplementary Figure 21
- Supplementary Figure 8 is now Supplementary Figure 22

In summary, this is a strong and carefully executed study that provides useful insight into the interplay of classical and non-classical interactions in aromatic hydration. The conclusions are largely supported by the data, and the manuscript should be of interest to a broad readership. I recommend publication after minor to moderate revision, mainly aimed at clarifying the interpretation of weak interactions and improving the overall clarity of the presentation.

Authors Response:

- *We thank the reviewer for the appreciation of our work and recommendation.*

Reviewer #3 (Remarks to the Author):

Key results
The authors investigate the hydration of phenol, aniline, and p-nitrophenol in solution using total neutron scattering combined with isotope substitution and structure refinement. Beyond classical hydrogen bonding, the study identifies non-classical interactions, including OH⋯π contacts, and provides evidence for π-hole lone pair interactions in aqueous solutions. The authors suggest that substituent-induced changes in π electron density modulate water structure and contribute to differences in solubility. The work addresses an important question, namely how subtle electronic effects influence solvation at the molecular level.

Validity
The experimental approach is appropriate and technically sound. The combination of neutron scattering and modeling provides detailed structural insights into interactions involving hydrogen.
However, the interpretation relies heavily on the refinement model, and the manuscript would benefit from a clearer discussion of the following points:
• the uniqueness of the structural solutions,

Authors Response:

- *The Monte Carlo simulation is the maximum entropy solution for the selected reference potential. In the case of the refined simulation, in which the contribution of the empirical potential is a relatively small perturbation to the reference "seed" potential, we obtain the least structured ensemble in agreement with the experimental data. Our analysis is averaged over >100,000 configurations, and therefore it is the most likely configuration in agreement with the experimental data.*
  Given that the model is constrained by 6 independent isotopic substitutions per system, input molecular geometry, and density, we are confident that any structuring that arises is the most likely configuration in agreement with the experimental data.

Changes to manuscript

- *Main Manuscript Page 9 Line 35: "As a traditional Monte Carlo method, it is a maximum entropy approach that avoids "over-structuring" of the liquids and solutions.[69]"*

• the robustness of the assignment of π-hole interactions.

Authors Response:

- *Our interpretation of the lone pair–π-hole comes exclusively from structural data, however it is corroborated by the position of the LUMO in the aromatic and the fact that the oxygen density above the ring is not perfectly centred but slightly off-set towards the OH group. We have added a paragraph to further clarify this point. Our interpretation agrees with solid state studies of similar systems.*

Changes to manuscript

- *Main Manuscript Page 6 Line 43: **"**In fact, the oxygen SDF sits slightly off-set towards the OH group, with the oxygen lone-pair in the direction of the LUMO.[16] Although we are not sensitive directly to electron distributions in our analysis, we can take our data as structural evidence for π-hole - Ow bonding in the liquid state, where the geometry of such an interaction is best described by the oxygen lone pair pointing towards the aromatic ring.[52]"*
- *Added Reference*
  - [52] A. Báuza *et al*, *Cryst. Growth Des*. **2016**, 16, 9, 5520.

In particular, the identification of π-hole lone pair interactions, while plausible, remains somewhat indirect and should be discussed with more caution. The proposed relationship between microscopic structure and solubility is interesting but not fully proven and may also involve competing effects such as dipole-driven aggregation.

Authors Response:

- *We agree that our argument about the microscopic structure and solubility needs to be more carefully addressed. We have therefore discussed self-association in more depth.*

Changes to Manuscript:

- *Main Manuscript Page 7 Line 15 "These non-conventional contacts have a clear effect on the overall hydration of p-nitrophenol, decreasing the overall water coordination from the theoretical value (from 36 to 29, Supplementary Table 5). The decrease can be ascribed to the concurrent lack of cooperation that does not facilitate water close approaches, with the consequent looser water structuring arising from the "linear" waters above and below the ring. In addition, there is a higher degree of self-association driven by dipole-dipole interactions that contribute to decrease solubility."*

Significance
The study provides valuable experimental evidence for nonclassical interactions between aromatics and water in the liquid state and shows that electronic modulation of the π system influences the structuring of water beyond classical hydrogen bonding. This is particularly relevant for systems in which solvation and aggregation compete with each other, such as π-conjugated arrangements and functional, especially soft, materials.

Authors Response:

- *We thank the Reviewer for the appreciation of our work.*

The manuscript would benefit from a stronger connection to previous work on substituent-dependent electron density distributions in small aromatic systems. In particular, differences or new insights compared to the corresponding solid-state X-ray crystallography and DFT-based and atoms-in-molecules analyses should be discussed.
https://pubs.acs.org/doi/10.1021/jp809601a
https://pubs.acs.org/doi/abs/10.1021/jp105348d
In addition, complementary computational approaches (e.g., CREST/xTB sampling

combined with DFT refinement) could be applied or discussed to clarify how efficiently OH···π and π hole interactions are currently captured with standard behavior and considered “good enough.”
https://pubs.acs.org/doi/10.1021/acs.jcim.9b01165
https://crest-lab.github.io/crest-docs/page/examples/qcg/example_5.html

Authors Response:

- *We agree that a link to solid state structures, electronic distributions, and computational understanding of solvation is valuable. We have therefore added the suggested references.*

Changes to Manuscript:

- *Main Manuscript Page 5 Line 15“It is interesting to note that the water structuring around the nitro group in solution is similar to that previously observed in the solid state. [50, 51]”*
- *Added References:*
    - [50] A. Báuza *et al.*, *Nat. Commun.* **2017**, 8, 14522.
    - [51] T. J. Mooibroek *et al.*, *ChemPhysChem* **2021**, 22, 141.
- *Main Manuscript Page 2 Line 25: “Electron distributions and molecular reactivities can be evaluated using a combination of theoretical methods and spectroscopic techniques. [14-16]”*
- *Added References:*
    - [14] M. Presselt *et al.*, *J. Phys. Chem. A* **2009**, 113, 13, 3210.
    - [15] M. Presselt *et al., J. Phys. Chem. A* **2010**, 114, 37, 10287.
- *Main Manuscript Page 8, Line 3: “Our analysis clearly shows the importance of solvation mechanisms in our understanding of weak competitive interactions, with the aim of informing the modelling of their effects on more complex behaviours, such as protein-ligand binding. [14-16, 55]”*
- *Added References:*
    - [14] M. Presselt *et al.*, *J. Phys. Chem. A* **2009**, 113, 13, 3210.
    - [15] M. Presselt *et al., J. Phys. Chem. A* **2010**, 114, 37, 10287.
    - [55] J. R. Loeffler *et al.*, *J. Chem. Inf. Model.* **2020**, 60, 4, 2304.

In principle, a brief discussion of relevance beyond biological systems would be valuable, for example, regarding ion or transport through aromatic molecular layers by means of templated water networks. Together with a corresponding extension of the discussion to interface systems, the general relevance would be strengthened, since water structuring at interfaces is inherently anisotropic, where such electronic effects are likely to be even more pronounced.

Authors Response:

- *We agree that these mechanisms are important beyond biological systems and we have broadened the discussion accordingly.*

Changes to Manuscript:

- *Main Manuscript Page 2 Line 4: "Beyond biological systems, water aromatic interactions dictate the structure of interfacial and nanoconfined water with impact on transport phenomena, interfacial self-assembly, and molecular adsorption. [8-10]"*
- *Added References:*
  - [8] M. L. Liriano *et al.*, *J. Am. Chem. Soc.* **2017**, 139, 6403.
  - [9] N. Li and M. D. Guiver, *Macromolecules* **2014**, 47, 7, 2175.
  - [10] Y. Wang *et al., Nature* **2021**, 600, 81.

Data, methodology and analytical approach
The data are of high quality and the methodology is very appropriate. The use of isotope substitution is an inherent strength.
The manuscript would benefit from the following points:
• clearer presentation of uncertainties in important structural parameters,

Authors Response:

- *We have added full sets of data with experimental uncertainties in the Supplementary Information file (Supplementary Figures 1-18). We have also clarified how the uncertainties are evaluated on structural information such as radial distribution functions and coordination numbers.*

Changes to Manuscript:

- *Added Supplementary Note 2: Neutron Scattering Data and Uncertainties*
- *Added Supplementary Figure 1 to 18 to the Supplementary Information File*
- *Main Manuscript Page 10 Line 23: "…over n>100,000 configurations, with uncertainties decreasing as* $\sim\sqrt{\frac{1}{n}}$*. "*

• more detailed discussion of the sensitivity of the refinement to model assumptions.
The analytical tools (radial distribution functions, coordination numbers, spatial density functions) are appropriate. A more careful distinction between directly observable quantities and model-dependent interpretations is recommended.

Authors Response:

- *The Monte Carlo simulation is the maximum entropy solution for the selected reference potential. In the case of the refined simulation, in which the contribution of the empirical potential is a relatively small perturbation to the reference "seed" potential, we obtain the least structured ensemble in agreement with the experimental data. Our analysis is averaged over >100,000 configurations, and therefore it is the most likely configuration in agreement with the experimental data.*
  Given that the model is constrained by 6 independent isotopic substitutions per system, input molecular geometry, and density, we are confident that any structuring that arises is the most likely configuration in agreement with the experimental data.

- *We note that the EPSR method is the evolution of the direct inversion methodology, from which partial distribution functions were obtained directly from the experimental data. In our systems the number of correlations is* $N = \frac{i(i+1)}{2} = 21$ *for phenol/water and aniline/water, and N=32 for p-nitrophenol/water. Practically, the inversion methodology does not allow a solutions uniquely constrained by the data due to the large number of isotopic substitutions. However, we note that these correlations are not independent, and by using the EPSR methodology we constrain molecular geometry, concentration, and density to experimental values. As a consequence, the degrees of freedom are reduced to molecule-molecule interactions (i.e. solute-solute, solute-solvent, solvent-solvent), and we capture those by extensively using isotopic substitution. We would also like to note that the molecular geometry is captured very well as seen by the excellent agreement between the measured and modelled total pair distribution functions of Supplementary Figure 23.*

Changes to Manuscript:

- *Main Manuscript Page 10 Line 2: "In principle, the partial radial distribution functions could be extracted directly from the neutron data using a numerical Fourier transform inversion methodology. However, given that the number of independent pair correlations for a system of i non-equivalent atomic sites is* $N = \frac{i(i+1)}{2}$*, the inversion methodology is not uniquely constrained by the experimental data. Here, we further reduce the degrees of freedom by inputting molecular geometries and physicochemical properties of the systems, such as concentrations and densities."*

Suggested improvements
The manuscript could be improved as follows:
1. Clarification of evidence for π-hole interactions and discussion of alternative interpretations.

Authors Response:

- *Our interpretation of the lone pair-π-hole comes exclusively from structural data, however it is corroborated by the position of the LUMO in the aromatic and the fact that the oxygen density above the ring is not perfectly centred but slightly off-set to the OH group. We have added a paragraph to further clarify this point. Our interpretation agrees with solid state studies of similar systems.*

Changes to Manuscript:

- *Main Manuscript Page 6 Line 43:* **"***In fact, the oxygen SDF sits slightly off-set towards the OH group, with the oxygen lone-pair in the direction of the LUMO. Although we are not sensitive directly to electron distributions in our analysis, we can take our data as structural evidence for π-hole - Ow bonding in the liquid state, where the geometry of such an interaction is best described by the oxygen lone-pair pointing towards the aromatic ring.[52]"*
- *Added Reference:*

- [52] A. Báuza *et al.*, *Cryst. Growth Des.* **2016**, 16, 9, 5520.

2. Discussion of the model dependency and robustness of the refinement results.

Authors Response:

- *The Monte Carlo simulation is the maximum entropy solution for the selected reference potential. In the case of the refined simulation, in which the contribution of the empirical potential is a relatively small perturbation to the reference "seed" potential, we obtain the least structured ensemble in agreement with the experimental data. Our analysis is averaged over >100,000 configurations, and therefore it is the most likely configuration in agreement with the experimental data.*
  Given that the model is constrained by 6 independent isotopic substitutions per system, input molecular geometry, and density, we are confident that any structuring that arises is the most likely configuration in agreement with the experimental data.
- *We note that the EPSR method is the evolution of the direct inversion methodology, from which partial distribution functions were obtained directly from the experimental data. In our systems the number of correlations is N=i(i+1)/2=21 for phenol/water and aniline/water, and N=32 for p-nitrophenol/water. The inversion methodology does not allow to be uniquely constrained by the data due to the large number of isotopic substitutions. However, we note that these correlations are not independent, and by using the EPSR methodology we constrain molecular geometry, concentration, and density. As a consequence, the degrees of freedom are reduced to molecule-molecule interactions (i.e. solute-solute, solute-solvent, solvent-solvent), and we capture those by extensively using isotopic substitution. We would also like to note that the molecular geometry is captured very well as seen by the excellent agreement between the measured and modelled total pair distribution functions of Supplementary Figure 23.*

Changes to Manuscript:

- *Main Manuscript Page 10 Line 2: "In principle, the partial radial distribution functions could be extracted directly from the neutron data using a numerical Fourier transform inversion methodology. However, given that the number of independent pair correlations for a system of i non-equivalent atomic sites is* $N = \frac{i(i+1)}{2}$*, the inversion methodology is not uniquely constrained by the experimental data. Here, we further reduce the degrees of freedom by inputting molecular geometries and physicochemical properties of the systems, such as concentrations and densities."*

3. Specification of uncertainties for important structural variables.

Authors Response:

- *We have added full sets of data with experimental uncertainties in the Supplementary Information file (Supplementary Figures 1-18). We have also*

clarified how the uncertainties are evaluated on structural information such as radial distribution functions and coordination numbers.

Changes to Manuscript:

- *Added Supplementary Figure 1 to 18 to the Supplementary Information File*
- *Main Manuscript Page 10 Line 23: "…over n >100,000 configurations, with uncertainties decreasing as ~*$\sqrt{\frac{1}{n}}$*. "*

4. Expansion of the discussion on alternative x-ray-based and DFT-based work.

Authors Response:

- *We agree that a link to solid state structures, electronic distributions, and computational understanding of solvation is valuable. We have therefore added the suggested references.*

Changes to Manuscript:

- *Main Manuscript Page 5 Line 15: "It is interesting to note that the water structuring around the nitro group in solution is similar to what previously observed in the solid state."*
- *Added Ref 50-51:*
  - [50] A. Báuza *et al.*, *Nat. Commun.* **2017**, 8, 14522.
  - [51] T. J. Mooibroek *et al.*, *ChemPhysChem* **2021**, 22, 141.
- *Main Manuscript Page 8 Line 1: "Our analysis clearly shows the importance of solvation mechanisms in our understanding of weak competitive interactions, with the aim of informing the modelling of their effects on more complex behaviours, such as protein-ligand binding.[14-16, 55]"*
- *Added References:*
  - [14] M. Presselt *et al.*, *J. Phys. Chem. A* **2009**, 113, 13, 3210.
  - [15] M. Presselt *et al., J. Phys. Chem. A* **2010**, 114, 37, 10287.
  - [55] J. R. Loeffler *et al.*, *J. Chem. Inf. Model.* **2020**, 60, 4, 2304.

5. Expansion of the discussion on relevance in various applications and material systems.

Authors Response:

- *We agree with the Reviewer that it is valuable to put our results in a broader context. We have modified the text accordingly.*

Changes to Manuscript:

- *Main Manuscript Page 2 Line 4: "Beyond biologically related systems, water aromatic interactions dictate the structure of interfacial and nanoconfined water with impact on transport phenomena, interfacial self-assembly, and molecular adsorption. [8-10]"*
- *Added references*
  - [8] M. L. Liriano *et al.*, *J. Am. Chem. Soc.* **2017**, 139, 6403

- [9] N. Li and M. D. Guiver, *Macromolecules* **2014**, 47, 7, 2175.
- [10] Y. Wang *et al., Nature* **2021**, 600, 81.

These points should be able to be addressed briefly within the current scope.

Authors Response:

- *We thank the Reviewer for their time and appreciation of our work. We believe that our manuscript has now improved in clarity and completeness, and we hope it is now suitable for publication.*

Clarity and context

The manuscript is clear and well structured. The discussion is insightful; a clearer distinction between experimental evidence and conceptual conclusions would be helpful.

Authors Response:

- *We thank the Reviewer for their comments and praise of our work.*

References

The literature is cited appropriately for the most part. A somewhat stronger connection to theoretical work and complementary X-ray-based studies (albeit on solids) would be desirable.

Authors Response:

- *We have added to the manuscript References 8, 9, 10, 14, 15, 16, 50, 51, 55. We believe that we now cover complementary theoretical and experimental work, as well as broader implications of our work in areas beyond biology.*

Changes to manuscript:

- *Main Manuscript Page 2 Line 4: "Beyond biologically related systems, water aromatic interactions dictate the structure of interfacial and nanoconfined water with impact on transport phenomena, interfacial self-assembly, and molecular adsorption."*
- *Added references:*
    - [8] M. L. Liriano *et al.*, *J. Am. Chem. Soc.* **2017**, 139, 6403
    - [9] N. Li and M. D. Guiver, *Macromolecules* **2014**, 47, 7, 2175.
    - [10] Y. Wang *et al., Nature* **2021**, 600, 81.

Your expertise

My expertise covers intermolecular interactions, solvation phenomena, and supramolecular systems composed of smaller and larger pi-conjugated molecules, particularly at aqueous interfaces. I am less specialized in neutron scattering experiments, but I am confident in assessing their chemical interpretation

Summary
This is a well-founded and technically sophisticated study that provides rare experimental insights into nonclassical interactions between aromatics and water in solution. While the identification of π-hole effects is fascinating and potentially important, the interpretation would benefit from a more cautious and general formulation.
With moderate revisions, the manuscript could make a valuable contribution to this field of research, particularly in areas where the interaction between electronic structure, solvation, and aggregation determines function.

Authors Response:

- *We thank the Reviewer for their time and appreciation of our work. We believe that our manuscript has now improved in clarity and completeness, and we hope it is now suitable for publication.*
